# Comprehensive analysis and accurate quantification of unintended large gene modifications induced by CRISPR-Cas9 gene editing

**DOI:** 10.1126/sciadv.abo7676

**Published:** 2022-10-21

**Authors:** So Hyun Park, Mingming Cao, Yidan Pan, Timothy H. Davis, Lavanya Saxena, Harshavardhan Deshmukh, Yilei Fu, Todd Treangen, Vivien A. Sheehan, Gang Bao

**Affiliations:** ^1^ Department of Bioengineering, Rice University, Houston, TX 77030, USA.; ^2^ Department of Computer Science, Rice University, Houston, TX 77005, USA.; ^3^ Emory University School of Medicine, Atlanta, GA 30322, USA.

## Abstract

Most genome editing analyses to date are based on quantifying small insertions and deletions. Here, we show that CRISPR-Cas9 genome editing can induce large gene modifications, such as deletions, insertions, and complex local rearrangements in different primary cells and cell lines. We analyzed large deletion events in hematopoietic stem and progenitor cells (HSPCs) using different methods, including clonal genotyping, droplet digital polymerase chain reaction, single-molecule real-time sequencing with unique molecular identifier, and long-amplicon sequencing assay. Our results show that large deletions of up to several thousand bases occur with high frequencies at the Cas9 on-target cut sites on the *HBB* (11.7 to 35.4%), *HBG* (14.3%), and *BCL11A* (13.2%) genes in HSPCs and the *PD-1* (15.2%) gene in T cells. Our findings have important implications to advancing genome editing technologies for treating human diseases, because unintended large gene modifications may persist, thus altering the biological functions and reducing the available therapeutic alleles.

## Teaser

CRISPR-Cas9–based genome editing can have a high level of unintended gene modifications including large deletions and insertions.

## INTRODUCTION

Over the past 10 years or so, clustered regularly interspaced short palindromic repeats (CRISPR)/CRISPR-associated protein 9 (Cas9) (CRISPR-Cas9) systems and their derivatives have emerged as a powerful tool for site-specific and permanent alterations to the genomes of a wide variety of organisms (*1*–*3*). Most of the CRISPR-Cas9 systems function by creating a DNA double-strand break (DSB) at the intended target locus in a cell, which is subsequently repaired by nonhomologous end joining (NHEJ), homology-directed repair (HDR), or microhomology-mediated end joining (MMEJ) pathway, resulting in targeted gene disruption, deletion, insertion, or correction (*3*). DSBs repaired by NHEJ result in small insertions and deletions (INDELs) of <50 base pairs (bp) at the Cas9 cut sites, which can be quantified accurately by targeted amplicon sequencing using short-range (S-R) polymerase chain reaction (PCR) followed by next-generation sequencing (S-R NGS).

Almost all gene editing applications require highly efficient cutting at the on-target site (*4*–*7*). However, high Cas9 cutting rates may result in detrimental off-target effects, including large chromosomal rearrangements such as chromosomal deletions, translocations, and inversions between the on- and off-target cut sites (*7*–*9*). While the off-target effects of CRISPR-Cas9 have received extensive studies and approaches have been developed to reduce off-target effects (*10*, *11*), the gene editing outcomes at the on-target cut sites have not been systematically studied, which are more complex than previously anticipated and thus merit further investigation. Recent reports indicated that, in addition to small INDELs, Cas9 cutting could induce large deletions (LDs; defined in most published reports as those larger than 200 bp) and large insertions (≥50 bp) at the on-target cut-site (*12*–*18*). However, it is very challenging to accurately quantify LDs, large insertions, and complex gene modifications because of Cas9 cutting. Although S-R NGS can quantify small INDELs with a low error rate (~0.1%), it cannot be used to quantify LDs and large insertions, because the standard paired-end library for S-R NGS is based on amplicons of up to 300 bp; thus, the maximum sizes of deletions and insertions that can be accurately quantified using S-R NGS are typically ∼100 bp for deletions and ~50 bp for insertions. With a few exceptions, almost all studies on genome editing to date only report small INDEL quantification (*4*, *5*, *7*). The extent and consequences of unintended large gene modifications at or near Cas9 on-target cut sites are largely unknown. Therefore, a comprehensive characterization and accurate quantification of the diverse gene editing outcomes, including LDs and large insertions, will facilitate the design, functional analysis, and application of CRISPR-Cas9–based genome editing.

Long-read sequencing technologies have the ability to generate reads of tens to thousands of kilobases in length, thus enabling the detection of large structural variations in the genome. For example, the Pacific Biosciences (PacBio) single-molecule real-time sequencing technology (SMRT-seq) and the Oxford Nanopore Technologies (ONT) Nanopore sequencing technology have been used for quantifying gene modifications induced by CRISPR-Cas9 (*12*, *19*–*21*). In particular, SMRT-seq uses a topologically circular DNA template for circular consensus sequencing (CCS) to improve the accuracy and generate long high-fidelity (HiFi) reads (*22*, *23*). Although PCR-based target sequence enrichment and long-read sequencing have been used to analyze CRISPR-Cas9–induced LDs (*12*, *16*, *21*), these approaches are limited by artifacts and PCR biases in multitemplate long-range PCR (L-R PCR) because of the high complexity of gene-edited alleles.

There is an unmet need to develop unbiased and quantitative methods to characterize the large genomic changes because of CRISPR-Cas9–induced DSBs, especially for therapeutic applications. This study provides a comprehensive analysis of large gene modifications at the Cas9 cut sites of different guide RNAs (gRNAs) in both cell lines and primary cells. We first performed clonal genotyping to quantify Cas9-induced large gene modifications and their zygosity in individual clones derived from sickle human umbilical cord–derived erythroid progenitor (S-HUDEP2) cells and hematopoietic stem and progenitor cells (HSPCs), respectively, edited by HiFi *Streptococcus pyogenes* Cas9 (*Sp*Cas9) complexed with the R-66S gRNA targeting the sickle mutation locus in the first exon of β-globin (*HBB*) gene [here referred to as R-66S ribonucleoprotein (RNP) complex] (*7*). The high frequency of LDs was confirmed using an L-R PCR gel shift assay and droplet digital PCR (ddPCR)–based allelic drop-off assay.

To provide detailed information on the sizes and distribution of LDs at the Cas9 on-target cut site, we performed L-R PCR–based sequencing assays, including PacBio HiFi SMRT-seq (*24*), ONT Nanopore sequencing (*25*), and Illumina NGS. Because L-R PCR amplification of genomic DNA (gDNA) could give rise to erroneous chimeras and heteroduplexes, it is challenging to accurately preserve the abundance and diversity of alleles in CRISPR-Cas9 gene-edited cells. Recently, Karst *et al.* (*26*) reported the use of L-R PCR with dual unique molecular identifier (UMI) in an attempt to mitigate the issues with PCR chimeras and amplicon length–dependent biases for long-read (>10 kb) gene sequencing of microbial communities. In this study, we combined the SMRT-seq with dual UMI and developed a bioinformatics pipeline to accurately quantify CRISPR-Cas9–induced small and large gene modifications in the bulk population of gene-edited cells. We constructed a DNA library with artificial LDs as the “standard” with predetermined allele frequencies to benchmark SMRT-seq with UMI. Quantitative analysis based on SMRT-seq with UMI revealed a high frequency and broad spectrum of LDs at the Cas9 cut sites in the *HBB* (*4*, *7*), γ-globin (*HBG*) (*27*), and B cell lymphoma/leukemia 11A (*BCL11A)* (*28*) genes in HSPCs and the *PD-1* gene in primary T cells, respectively. We found that LDs in gene-edited HSPCs persisted after in vitro erythroid differentiation, necessitating further investigation of the functional consequences of LD-carrying HSPCs.

SMRT-seq requires specialized sample preparation and often is only available at core facilities. To enable high-throughput determination of both small INDELs and LDs at the Cas9 cut sites, we developed the long-amplicon sequencing (LongAmp-seq) assay based on Illumina NGS of the fragmented L-R PCR products, which can be easily implemented by a laboratory experienced with S-R NGS. We demonstrated that LongAmp-seq could provide both small INDEL and LD profiles, detailed sequence information, and fairly accurate quantification of LD alleles in one assay. Using the LongAmp-seq assay, we performed a preliminary study on LD repair mechanism and kinetics. Together, our study provided a comprehensive analysis of gene editing outcomes by five Cas9/gRNA RNPs in cell lines, HSPCs, and T cells; revealed high levels of unintended gene modifications; and demonstrated the need for more careful evaluation of gene editing outcomes, especially for therapeutic genome editing using CRISPR-Cas9.

## RESULTS

### Clonal genotyping of gene-edited S-HUDEP2 cells and HSPCs reveals a high rate of LDs at the *HBB* on-target cut site

**Fig. 1.**
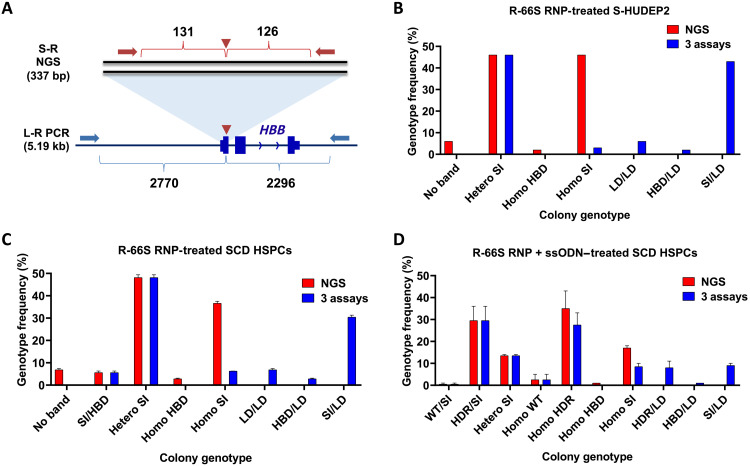
The high proportion of LD alleles and genotypes in R-66S RNP-treated S-HUDEP2 and SCD HSPCs. The genotype of each colony was identified by S-R NGS, L-R PCR, and ddPCR to account for the dropout of LD alleles. (**A**) S-R and L-R PCR primer designs to amplify the region around the R-66S cut site on *HBB*. (**B**) Genotype results based on S-R NGS with the combination of the three assays for 100 clones derived from S-HUDEP2 treated with R-66S RNP. (**C**) Genotype results based on S-R NGS with the combination of the three assays for 72.5 ± 12.0 erythroid colonies derived from SCD HSPCs treated with R-66S RNP. The use of S-R NGS significantly overestimated the percentage of small INDEL (SI) alleles compared with that identified using the combination of three assays. 23.4 ± 0% LD alleles occurred in 40.1 ± 0.8% colonies, which caused a significant reduction of *HBB* copy numbers in RNP-treated SCD HSPCs. (**D**) Genotype results for 79 ± 7 erythroid colonies derived from SCD HSPCs treated with both R-66S RNP and the corrective ssODN donor. A total of 11.8 ± 0.8% of alleles had LD in 18.5 ± 2.9% of colonies. S-R NGS overestimated the percentage of homozygous HDR colonies (35.4 ± 11.3%) compared to that obtained by the combination of three assays (27.2 ± 7.5%).

To determine the frequency of LDs at the on-target cut site and how CRISPR-Cas9 editing frequencies in the bulk population are related to the zygosity in individual cells, we first performed clonal genotyping of gene-edited S-HUDEP2 cells (*29*), which was created by introducing the sickle mutation in *HBB* of HUDEP2, an immortalized CD34^+^ hematopoietic stem cell (HSC)–derived erythroid precursor cell line (fig. S1) (*29*). S-HUDEP2 cells were electroporated to deliver RNP formed by Integrated DNA Technologies (IDT) Alt-R HiFi *Sp*Cas9 (*7*, *10*) and the R-66S gRNA targeting the sickle cell disease (SCD) mutation site in *HBB* (table S1) (*7*). Unless stated otherwise, all gene editing experiments were performed using RNPs formed by this HiFi *Sp*Cas9 and different gRNAs. S-R NGS identified the genotype of each single cell–derived clone to detect small INDELs together with two complementary methods to account for the dropout of LD alleles: (i) L-R PCR followed by gel shift assay and (ii) ddPCR-based *HBB* allelic drop-off assay (fig. S2). The L-R PCR primers amplified the 5.19-kb region containing the R-66S gRNA-defined on-target cut site at the center, and the alleles containing an LD between the L-R PCR primer binding sites generated PCR products with smaller sizes (Fig. 1A). L-R PCR amplification resulted in smearing and downward size-shifted bands on an agarose gel in R-66S RNP-treated samples (bulk cell culture) compared to the untreated sample, indicating LDs in *HBB* (fig. S3). A representative agarose gel image showing the S-R and L-R PCR products of representative eight single-cell clones from R-66S RNP-edited S-HUDEP2 cells is displayed in fig. S4.

Figure S5 summarized how the genotype of 100 S-HUDEP2 clones derived from R-66S RNP-edited cells were determined. With S-R NGS, 46 clones were found to have heterozygous small INDELs (i.e., two alleles have different small INDELs) and 46 clones with homozygous small INDELs (i.e., both alleles have the same small INDEL) (table S2). We previously showed that Cas9 cutting–induced DSBs in *HBB* could be repaired using the homologous sequences from the δ-globin gene (*HBD*) as an endogenous template, resulting in SCD mutation correction (*7*). We found that two clones had homozygous SCD mutation correction mediated by *HBD* gene conversion, and six clones failed to produce S-R PCR products. As expected, no genotype with LD could be identified by S-R PCR. Because clones with an LD in one allele could be falsely identified as homozygotes in S-R NGS, we attempted to identify false-positive homozygotes by L-R PCR gel shift assay. However, in some cases, alleles with LD that removed the L-R PCR primer binding site(s) or with chromosomal rearrangement could not be amplified. The clonal genotype of eight S-HUDEP2 clones determined by the combination of three assays is shown in fig. S6.

To determine the frequency of LD alleles, we quantified the copy number of *HBB* relative to a reference gene (*CACNA1C*) using the ddPCR-based allelic drop-off assay in which one drop-off (*HBB*) primer pair and one reference primer pair were used. The *HBB* primers span the Cas9 on-target cut site with a forward primer binding site 68 bp upstream and a reverse primer binding site 108 bp downstream of the on-target cut site (fig. S2A), whereas the reference primers bind to regions in the reference gene. For an unmodified or small INDEL–containing allele, both the forward and reverse *HBB* primers could bind to their complementary regions, resulting in a positive count of the *HBB* allele in the ddPCR assay. When an LD occurs, at least one *HBB* primer binding site is lost, yielding no *HBB* allele count in the ddPCR assay. The ratio of the *HBB* allele number and that of the reference gene reveals the genotype, as illustrated by the examples in fig. S2D.

As shown in Fig. 1B, the combination of the three assays revealed multiple genotypes of the single-cell clones from R-66S RNP-edited S-HUDEP2 cells: Of the 46 clones identified as homozygous small INDEL genotype by S-R NGS, only 4 clones were homozygous while 42 clones carried LD. We found that the six clones that failed to amplify the S-R PCR product all had LD/LD genotype, and the two clones with HBD conversion carried LD (fig. S7A). Twenty-eight percent of LD alleles occurred in 50% of clones, which caused a significant reduction of *HBB* copy numbers in gene-edited S-HUDEP2 cells. The percentage of intact *HBB* alleles quantified by the ddPCR assay can be used to approximate the rate of unmodified and small INDEL alleles out of the total *HBB* alleles, including that containing LD with allelic drop-off. Therefore, we inferred the LD-adjusted allele frequency in the bulk population by adjusting the small INDEL rate quantified by S-R NGS on the basis of the percentage of the intact *HBB* alleles. We found that S-R NGS significantly overestimated the percentage of small INDEL alleles (97.8%) compared with the LD-adjusted small INDEL allele frequency (71%) (fig. S7B).

We used the same strategy to analyze the clonal cell populations from the erythroid colonies from gene-edited HSPCs from patients with SCD (SCD HSPCs) after colony formation assays. A total of 154 erythroid colonies derived from R-66S RNP-edited SCD HSPCs were analyzed to obtain their genotypes. Figure 1C compares genotyping results obtained using the S-R NGS and the combination of three assays (S-R NGS, L-R PCR, and ddPCR). The combination of three assays revealed different genotypes of the single-cell clones: Of the 36.7 ± 1.2% colonies identified as homozygous small INDEL genotype by S-R NGS, only 6.2 ± 0.1% were homozygous small INDELs, while 30.4 ± 1.1% of the colonies carried a LD with small INDEL/LD genotype. A total of 6.8 ± 0.8% colonies that failed to amplify S-R PCR product all had LD/LD genotype, and the 2.8 ± 0.5% colonies with *HBD* conversion carried LD (HBD/LD). The use of only S-R NGS overestimated the percentage of small INDEL alleles (87.6 ± 0.1%) compared with the LD-adjusted small INDEL alleles identified using the combination of three assays (72.4 ± 0.7%). More significantly, 40.1 ± 0.8% colonies had LD-containing alleles, which were missed by S-R NGS, resulting in a significant genotype miscall in SCD HSPCs (table S3).

One of the strategies in treating single-gene disorders is to use a DNA donor template to correct the gene defect via the HDR pathway, such as the correction of sickle mutation in *HBB* for curing SCD (*7*). To determine whether the presence of a corrective donor template will change the LD rates, we performed clonal genotyping in SCD HSPCs delivered with R-66S RNP and the corrective single-stranded oligodeoxynucleotide (ssODN) donor for gene correction (Fig. 1D) (*7*). We found that 18.5 ± 2.9% of the colonies had LDs, with genotypes of HDR/LD (8.2 ± 3.8%), small INDEL/LD (9.0 ± 1.4%), and HBD/LD (1.0 ± 0.0%), where “HDR” and “HBD” indicate the HDR-mediated gene correction using the ssODN donor delivered and with the endogenous sequence in *HBD*, respectively (Fig. 1D and table S4). The S-R NGS overestimated the percentage of homozygous colonies with HDR-mediated gene correction (35.4 ± 11.3%) compared with that obtained by the combination of three assays (27.2 ± 7.5%) (Fig. 1D and table S4). The high rate of LD in *HBB* in a subpopulation of gene-edited HSPCs may have significant implications to inducing β-thalassemia major or minor because of *HBB* knockout (KO). According to the colony genotype in SCD HSPCs, 42% of RNP-only and 20% of RNP + ssODN gene-edited colonies had total *HBB* KO from frameshift small INDEL or LD on both alleles (table S4). Our findings suggest that the previously reported therapeutic gene correction rates were overestimated (*4*, *5*, *7*), and the risk of inducing β-thalassemia from gene editing because of *HBB* KO needs to be carefully evaluated. The clonal genotype results of S-HUDEP2 cells gene-edited with both R-66S RNP and ssODN donor showed comparable results as in SCD HSPCs (figs. S8 and S9).

### SMRT-seq with dual UMI for analyzing large gene modifications

**Fig. 2.**
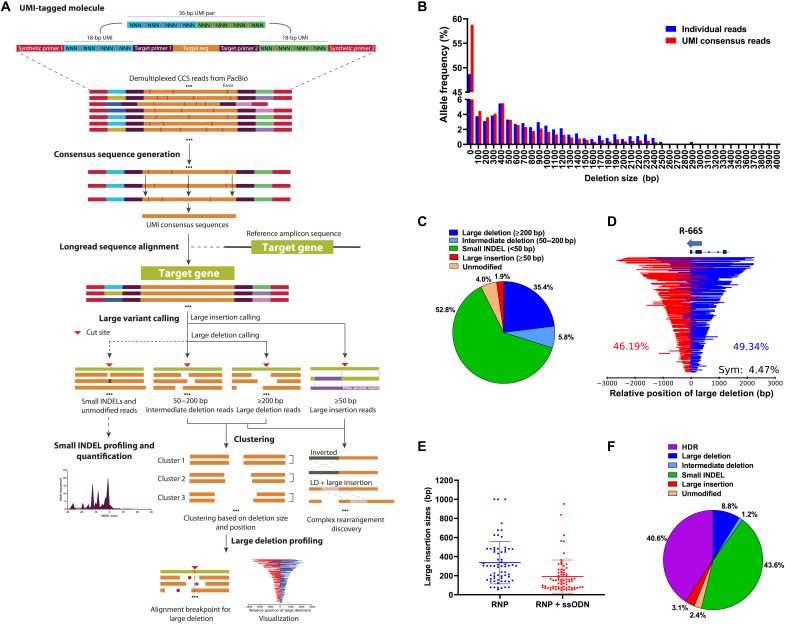
Comprehensive quantification of gene editing outcomes using SMRT-seq with UMI. (**A**) Schematics of SMRT-seq with UMI processing and variant calling pipeline. Each DNA molecule of 5- to 6-kb genomic region around the Cas9 cut site was tagged with dual UMIs. Demultiplexed HiFi CCS reads were processed by the longread_umi pipeline to generate UMI consensus reads. The LV_caller pipeline was developed to align the reads to the reference amplicon sequence for identification of gene modifications. (**B**) LD size histogram plotted using individual CCS reads and UMI consensus reads showing a reduced rate for LDs of >400 bp and increased rate for LDs of <400 bp as a result of UMI-based PCR duplicate removal. (**C**) Comprehensive allele frequencies in UMI consensus sequences showing high rates of diverse large gene modifications induced by R-66S RNP, including LDs of ≥200 bp, intermediate deletions of 50 to 200 bp, and large insertions of ≥50 bp. (**D**) LD patterns were mapped relative to the Cas9 cut site to show deletion size and location. Most LDs are unsymmetrically located, with the center of LD on the upstream (red) or downstream (blue) side of the cut site. Percentages of upstream, downstream, and symmetric LDs are shown. Arrow shows the 5′ to 3′ orientation of the R-66S gRNA. (**E**) Large insertion size distribution by R-66S RNP with and without the corrective ssODN. Mean large insertion size decreased with ssODN. (**F**) Comprehensive allele frequencies in the R-66S RNP + ssODN–treated sample. LD rate was reduced in the presence of ssODN. All results were from HSPCs of SCD patient Donor #1.

To accurately quantify CRISPR-Cas9–induced LDs, large insertions, and chromosomal rearrangements in gene-edited cells, we combined L-R PCR–based SMRT-seq with dual UMI tagging (Fig. 2A) (*26*). The PCR reaction with two amplification cycles (PCR1) was used to target 5- to 6-kb genomic region around the Cas9 cut site and simultaneously tag the 5′ and 3′ ends of each gDNA molecule with 18-bp terminal UMI using a tailed primer pair (table S5). The first section of both tailed primers is a synthetic priming site used for downstream amplification, followed by the 18-nucleotide “patterned” UMI (NNNYRNNNYRNNNYRNNN) and target-specific sequences (*26*). After UMI labeling, each strand of the DNA duplex is tagged with a unique combination of dual UMI. A second PCR (PCR2) was used to amplify the UMI-tagged DNA molecules. A third PCR (PCR3) was used to reamplify the PCR2 product for generating barcoded amplicons for multiplexed sequencing. The final barcoded PCR3 amplicon product contains the symmetric barcode sequences at both ends. Up to 24 UMI-tagged and barcoded amplicon samples from PCR3 were pooled, and a total of 1 μg of the pooled amplicons was used for SMRT-seq library preparation. The SMRTbell library was sequenced on a PacBio Sequel II 8M flow cell in CCS mode, and HiFi reads were produced. HiFi reads with >Q20 (quality score of 20) (99%) and an average of Q30 (99.9%) single-molecule read accuracy were demultiplexed and processed by the longread_umi pipeline to generate UMI consensus reads (*26*). We developed a bioinformatics toolkit called LV_caller to analyze the UMI consensus sequences and quantify the gene editing outcomes. The LV_caller pipeline (Fig. 2A) aligns the UMI consensus sequences to the reference amplicon sequence and identifies gene modification variants, which are categorized into four groups: (i) unmodified sequences or those with small INDELs of <50 bp, (ii) intermediate deletions with sizes between 50 and 200 bp, (iii) LDs of ≥200 bp, and (iv) large insertions of ≥50 bp (Fig. 2A and fig. S10). Because most of the published reports define LDs as >200 bp, but deletions with sizes between 50 and 200 bp do occur, which were largely overlooked by the previous studies, we define these deletions as “intermediate deletions” and quantified the rates. UMI consensus sequences containing unmodified and/or small INDEL alleles were converted to a bam format and analyzed by CRISPResso2 to quantify the small INDEL profile (*30*). LDs were analyzed on the basis of the split read breakpoint alignment, and the LDs sharing the same alignment pattern (defined as the combination of LD size and location) were clustered, and the clustered reads were used for LD profiling and visualization (Fig. 2A). Large insertion sequences were mapped to the Hg19 reference genome using BLAST-like alignment tool (BLAT) (*31*). For each UMI consensus read carrying large insertions, the chromosomal location of the insertion site, length of the matched or mismatched bases, strand orientation of the inserted sequences, and other gene modification variants accompanying large insertions were retrieved.

To benchmark the SMRT-seq with UMI using known mixtures of allelic variants, we constructed a synthetic DNA library as the standard, consisting of a wild-type (WT) *HBB* sequence of 5490 bp (template 9) and templates 1 to 8 with artificial LDs of eight different sizes (4416, 3872, 3408, 3079, 2415, 1926, 1408, and 921 bp, respectively; fig. S11A). Each DNA template was assigned a 6-bp allele-specific barcode at the 5′ end to verify the accuracy of LD variant calling. The nine plasmid templates were linearized and pooled with specific molar ratios, with 80% of template 9 and 20% of templates 1 to 8 combined. The relative percentages of templates 1 to 9 in the pooled plasmid sample were quantified by duplex probe-based ddPCR using template barcode–specific primer pairs and a reference primer pair binding to all templates. The synthetic DNA library was then used as templates for a three-step L-R PCR to generate UMI-tagged and barcoded PCR3 products, which were sequenced using SMRT-seq to quantify the percentages of templates 1 to 9. On the basis of the aligned CCS reads, template 9 in PCR3 product was 54.38%, significantly decreased from 79.9% in the original standard quantified by ddPCR, largely because of PCR errors and SMRT cell loading bias (fig. S11B). When using UMI consensus sequences (i.e., UMI pairs with three or more CCS reads) to quantify templates 1 to 9, we found that the percentages are in good agreement with the ddPCR results. In particular, the percentage of template 9 was 78.13%, very close to the allele frequency (79.9%) in the original template sample (fig. S11B). This is due to the removal of the PCR duplicates and false-positive LDs in the aligned CCS reads using the UMI consensus reads (fig. S11, C and D). Our benchmarking results suggest that SMRT-seq with UMI can accurately quantify the total percentage of LDs in gene-edited cells.

### Quantification of large gene modifications at *HBB* induced by R-66S RNP

We used SMRT-seq with UMI to quantify both small and large genetic variants introduced by R-66S gRNA/Cas9 RNP in SCD HSPCs. We obtained 34,055 demultiplexed HiFi CCS reads aligned to the reference *HBB* sequence. UMI pairs with three or more CCS reads were used for UMI consensus sequence generation to remove PCR errors based on the sequence information within each CCS read derived from the same SMRTbell template molecule. For example, from one R-66S RNP-treated sample, we identified 3473 UMI consensus sequences. Alignment of UMI consensus sequences to *HBB* showed read coverage depletion pattern around the R-66S cut site only in the RNP-treated sample, not in the control sample (fig. S12), showing the profile of LDs. In addition to the sickle mutation, 11 other single-nucleotide polymorphisms (SNPs) were found in the genome of the particular patient with SCD (Donor #1) compared to the reference genome (fig. S12A). We detected diverse LDs of up to 4350 bp and insertions of up to 1000 bp. The untreated control sample showed no evidence of sequence variation. By comparing the LD size histograms plotted using raw CCS reads and UMI consensus reads, we found that the UMI-based PCR duplicate removal led to a decreased rate of LDs larger than 400 bp and an increased rate of LDs less than 400 bp (Fig. 2B).

For the R-66S RNP-treated SCD HSPC sample, we found 35.4% of LDs (≥200 bp), 5.8% of intermediate deletions (50 to 200 bp), and 1.9% of large insertions (≥50 bp), in addition to 52.8% small INDELs (Fig. 2C). From the sample containing 3473 UMI consensus sequences, we identified 1229 LD-containing sequences that form 381 unique LD patterns, demonstrating a diverse range of LDs. LDs sharing the same alignment pattern (defined as the combination of LD size and location) were clustered, and the clustered reads were used for LD profiling and visualization. Each of the 381 unique LD patterns was mapped relative to the Cas9 cut site to show the distribution of LD size and location (Fig. 2D). Of the 381 unique LD patterns, 130 were captured by one UMI consensus sequence, 90 by two UMI consensus sequences, and 46 by three UMI consensus sequences (fig. S13). Note that 21 UMI consensus sequences have the LD of the same size (267 bp) and start position, accounting for 0.6% of the total UMI consensus sequences. LDs have a very broad distribution of sizes and locations. In particular, a high percentage of LDs could lead to the disruption of the *HBB* promoter, which is 100 bp preceding exon 1 of *HBB* (Fig. 2D). Most LDs spanned the Cas9 cut site, although not symmetric about it (Fig. 2D). To quantify the percentages of symmetric and asymmetric LDs, we define δ = *X*/*Y*, where *X* is the number of base pairs from the midpoint of LD to the cut site and *Y* is the “LD size” (bp). If δ is ≤0.05, then the LD is considered as symmetric; otherwise, it is asymmetric. There was a slightly higher percentage of LDs (~49.3%) occurring downstream of the Cas9 cut site in *HBB* compared to the upstream ones (~46.2%). While each LD can be a rare event; collectively, they account for a large fraction of editing outcomes. A small number of LDs occurred at least 20 bp away from the Cas9 cut site, and some alleles contained multiple deletions (Fig. 2D and fig. S14).

In addition to LDs, we found that 1.9% of UMI consensus reads contained large insertions ranging from 59 to 1000 bp (Fig. 2, C and E). All large insertions occurred at the R-66S RNP-induced cut site, indicating that they are due to DSB repair. All of the inserted sequences are mapped to specified locations in the human genome with either perfect match or minimal mismatches, thus ruling out artifacts from sequencing or alignment (table S6). In some cases, large deletions and large insertions occurred simultaneously, demonstrating complex local chromosomal rearrangements within the β-globin locus. Most of the inserted sequences are homologous to *HBB* sequences at or close to the cut site with forward or inverted orientations (fig. S15 and table S6). Some of the inserted sequences are mapped to other chromosomal locations in the human genome, but they are not associated with previously predicted or validated R-66S gRNA off-target sites, thus not due to off-target cutting (fig. S16 and table S6). Note that, in some cases, large insertions with the same size and location were captured by multiple UMI consensus sequences, indicating that they were derived from multiple input gDNA alleles (table S6). It is possible that some of the inserted sequences are those in close proximity to the Cas9 cut site at the time of DSB repair because of the three-dimensional structure of the chromosome. This hypothesis remains to be validated using DNA cross-linking and sequencing or sequence-specific fluorescent labeling of genomic loci.

Our SMRT-seq results revealed an unexpectedly broad spectrum of unintended intermediate deletions, LDs, and large insertions at or near the Cas9 cut site in *HBB*. Of the 3478 UMI consensus sequences, a total of 536 unique gene modification patterns were identified, including 67 small INDELs, 44 intermediate deletions, 381 LDs, and 44 large insertions. Note that each gene modification pattern may be represented by one or multiple UMI consensus sequences. The SMRT-seq identified allelic diversity (536 unique gene modification patterns including large modifications) is >8-fold higher than characterized based on small INDELs (67 small INDEL patterns) (fig. S17).

We further applied SMRT-seq with UMI to quantify gene modifications in the presence of a corrective ssODN donor template (Fig. 2F). Similar to what was observed by clonal genotyping shown in Fig. 1, compared with that of the RNP-only treated sample, in the sample treated with both R-66S RNP and ssODN, the LD rate decreased from 35.4 to 8.8%, and the intermediate deletion rate decreased from 5.8 to 1.2%, likely due to prompt repairing of DSB by the HDR pathway (*32*). On the other hand, the large insertion rate increased from 1.9 to 3.1%, suggesting more complex local rearrangement in the presence of ssODN. With ssODN, the size distributions of LDs and large insertions are different compared to RNP-treated sample, with lower rates of shorter LDs and higher rates of longer LDs (fig. S18), as well as decreased mean large insertion size (from 336 to 190 bp; Fig. 2E). Our results are consistent with the previous report that the LD rate was reduced in the presence of ssODN or AAV-packaged donor (*32*).

### Large gene modifications are common for gRNAs with different gene targets

In addition to R-66S gRNA, we applied SMRT-seq with UMI to quantify both small and large gene modifications in SCD HSPCs induced by three other CRISPR gRNAs, including R-02 (*33*), SD-02 (*27*), and BCL11A gRNAs designed for treating SCD or β-thalassemia (*28*). The R-02 gRNA generates a DSB on the first exon of *HBB* 16 bp away from the sickle mutation site (*33*). The SD-02 gRNA introduces a 13-bp HPFH (hereditary persistence of fetal hemoglobin) deletion in the *HBG1*/*HBG2* promoter region to activate fetal hemoglobin (HbF) (*27*). The BCL11A gRNA targets the GATA1 site in the *BCL11A* erythroid enhancer region to induce HbF expression (table S1) (*28*). All gRNAs showed high on-target small INDEL rates and the expected small INDEL profiles measured by S-R NGS, similar to that previously reported (figs. S19 and S20) (*4*–*7*, *27*, *28*).

**Fig. 3.**
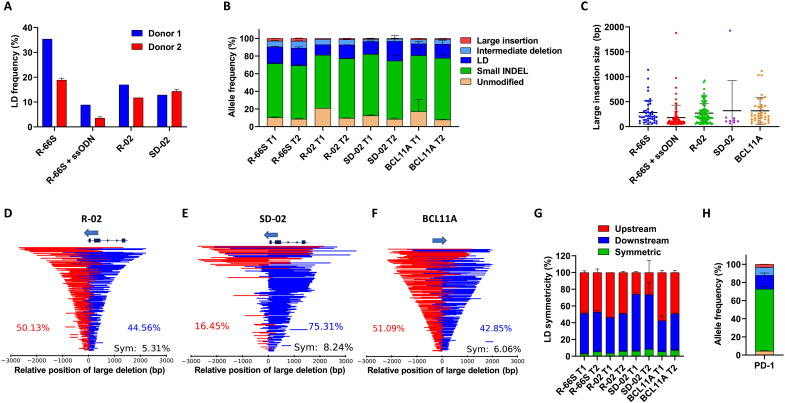
Unintended large gene modifications are common for CRISPR gRNAs. (**A**) Comparison of the LD rates in SCD HSPCs from two SCD patients (Donor #1 and Donor #2) analyzed by SMRT-seq for R-66S RNP with and without corrective ssODN, R-02 RNP, and SD-02 RNP. (**B**) SMRT-seq with UMI-based quantification of LD, intermediate deletion, large insertion, and small INDEL. gDNAs from SCD HSPCs edited by R-66S, R-02, SD-02, and BCL11A RNPs were collected on day 4 after delivery (T1) and from cells after 14 days of erythroid differentiation (on day 17 after delivery) (T2). High frequencies of diverse LDs of up to 4 kb and insertions of up to 1.9 kb were found at all loci tested. After 2 weeks of erythroid differentiation of SCD HSPCs, the LDs persisted and their rates increased. (**C**) Large insertion size distribution (50 bp to 1.9 kb) in RNP-treated samples from Donor #2. (**D**) LD profile (location and size) at *HBB* in the R-02 RNP-treated sample. (**E**) LD profile at *HBG1* in the SD-02 RNP-treated sample. (**F**) LD profile at *BCL11A* in the BCL11A RNP-treated sample. In (D) to (F), schematics of HBB and HBG1 genes are shown to scale; the schematic of BCL11A gene is not shown; arrows show the 5′ to 3′ orientation of the gRNAs. (**G**) Frequencies of LD positions relative to the Cas9 cut site (upstream, downstream, or symmetric) for four RNP-treated samples at T1 and T2. (**H**) SMRT-seq with UMI quantification of allele frequency in PD-1 RNP-treated primary human T cells. The color code is the same as in (B).

Because of the need to determine the persistence of large gene modifications, we performed SMRT-seq with UMI using HSPCs from a different patient with SCD (Donor #2). The gDNAs from SCD HSPCs edited respectively by R-66S, R-02, SD-02, and BCL11A RNPs were collected on day 4 after delivery and from cells after 14 days erythroid differentiation (started 3 days after delivery). Twenty-four samples were sequenced on a SMRT cell, generating an average of 85,847 HiFi CCS reads (average of >Q30) per sample. CCS reads contained high-confidence UMI pairs that were binned into an average of 949 UMI groups per sample, generating UMI consensus reads. Figure S21 shows the number of CCS reads and the UMI consensus sequences obtained for each sample. Although the SMRTbell library was pooled in equimolar ratios, the rate of UMI consolidation varied between samples showing the variations in UMI-tagging efficiency and PCR bias. UMI consensus reads were mapped to the expected amplicon sequences based on Hg19 for each locus. A comparison of the LD rates in SCD HSPCs from Donor #1 and Donor #2 revealed moderate differences (Fig. 3A), consistent with our previous results (*7*). Untreated samples from the particular patient with SCD had multiple SNPs compared to the Hg19 reference genome. We found that Donor #2 has 11 SNPs in *HBB*, 22 SNPs in *HBG1*, and 8 SNPs in *BCL11A*. Considering the diversity of LDs and sequence-dependent LD profiles, SNPs near the Cas9 cut site may alter the LD rate and profile, necessitating the use of a personalized genome for sequence mapping. In this work, the patient-specific genome sequences were used as the reference in LV_caller for sequence analysis.

High frequencies of diverse LDs of up to 4 kb and insertions of up to 1.9 kb were found in samples treated with R-66S, R-02, SD-02, and BCL11A RNPs (Fig. 3, B to F), while untreated samples did not show any evidence of specific sequence variation. The use of UMI consensus reads in quantifying the frequencies of LDs, intermediate deletions, and large insertions led to more accurate results than that from the CCS reads, because some of the PCR biases were corrected (fig. S22). As shown in Fig. 3B, R-02 RNP induced 11.7% LDs, 6.1% intermediate deletions, and 1.1% of large insertions. Although R-02 RNP had a similar total on-target editing rate as R-66S RNP, Cas9 cutting at the R-02 locus generated a significantly lower rate of LDs (Fig. 3B). Because both the R-66S and R-02 gRNAs target sequences in *HBB* near the sickle mutation site (*7*, *33*), this suggests that the generation of LDs is sensitive to the specific gRNA target sequence. Furthermore, R-02 RNP induced a 9-bp deletion as a primary small INDEL repaired by MMEJ using 5-bp proximal microhomologies (CTGCC), thus having a higher proportion of MMEJ-led small INDELs compared to that induced by R-66S RNP, which has a more diverse small INDEL profile and a lower proportion of MMEJ-led small INDELs (fig. S20, A and B). All small deletions greater than or equal to 3 bp were considered as MMEJ products (*34*). The R-02 RNP-induced LDs relative to the Cas9 on-target cut site are shown in Fig. 3D, with a slightly higher percentage of LDs (~54%) occurring upstream of the Cas9 cut site in *HBB* compared to the downstream ones (~46%).

Gene modifications introduced by SD-02 RNP were amplified using the *HBG1*-specific L-R PCR with a 6.4-kb amplicon size. As shown in Fig. 3B, SD-02 RNP induced 14.3 ± 0.7% LDs, 2.65 ± 0.6% intermediate deletions, and 1.05 ± 0.2% of large insertions. The SD-02 RNP-induced LDs relative to the Cas9 on-target cut site are shown in Fig. 3E, demonstrating that ~78.5% of LDs occurred downstream of the Cas9 cut site on *HBG1*. Because there is another on-target cut site in *HBG2*, located 4.9-kb upstream of *HBG1*, due to the *HBG1*-specific amplification, we were not able to detect the 4.9-kb intergenic deletions or rearrangements between simultaneous on-target cutting in *HBG1* and *HBG2*, which has been previously reported to occur at ~30% measured by ddPCR (*35*). To understand the types of large intergenic modification missed by *HBG1*-specific sequencing, we amplified and sequenced the 10-kb region, including *HBG1* and *HBG2*, and observed diverse intergenic LDs extending further upstream of the cut site on *HBG2* and/or downstream of the cut site on *HBG1*, removing *HBG1* and/or *HBG2* (fig. S23). Our results highlight the importance of examining larger genomic loci to comprehensively analyze gene editing outcomes.

We quantified the gene modifications in SCD HSPCs due to BCL11A RNP, including 13 ± 2.7% LDs, 4.7 ± 1.6% intermediate deletions, and 1.5 ± 0.0% large insertions (Fig. 3B). The BCL11A RNP-induced LDs relative to the Cas9 on-target cut site are shown in Fig. 3F. Site-specific disruption of the GATA1 motif in intron 2 of *BCL11A* eliminates the *BCL11A* expression in an erythroid-specific manner for HbF induction (*28*). The risk of inactivating *BCL11A* in nonerythroid cells or producing *BCL11A* isoforms due to LDs of several kilobase in size warrants further investigation, as an abnormal expression of *BCL11A* in HSCs has been implicated in impaired engraftment potential (*36*) and lymphoid development (*37*).

In addition to LDs, R-66S, R-02, SD-02, and BCL11A RNPs induced 3.0 ± 0.1%, 1.1 ± 0.0%, 1.05 ± 0.2%, and 1.5 ± 0.0% large insertions (50 bp to 1.9 kb), respectively, at the Cas9 cut site in SCD HSPCs (Fig. 3C). Most inserted sequences mapped to the targeted loci in either strand orientation, suggesting complex local chromosomal rearrangement. The rest of the inserted sequences are mapped to the other chromosomal locations in the human genome, warranting further investigation of the mechanism of the large insertions.

As shown in Fig. 3 (D to F), most LDs were asymmetric and extended to either side of the Cas9 cut site, consistent with the previous reports (*15*, *38*). For each of the four gRNAs, more than 90% of LDs are asymmetric (Fig. 3G). Unlike small INDELs, which typically have a few distinct peaks, we found that LDs have a very broad distribution of sizes and locations. The biological replicate showed a comparable allele frequency of LDs and their size distribution, but the unique LD patterns differ, further demonstrating the diverse range of LD generation in different cells. The distribution of LDs is dependent on the specific gRNA, suggesting a sequence-dependent repair process. While each LD can be a rare event, collectively, they account for a large fraction of edited alleles.

We found that, for the four gRNAs (R-66S, R-02, SD-02, and BCL11A) tested, after 2 weeks of erythroid differentiation of SCD HSPCs, the LDs persisted, and their rates increased (Fig. 3B), demonstrating a significantly reduced level of therapeutic allele compared to that determined using S-R NGS alone. The SMRT-seq–identified allelic diversity, including large gene modifications, is 4.4- to 9.8-fold higher than characterized based on small INDEL (fig. S24). Together, our results demonstrate unexpected and unintended gene editing outcomes by the four gRNAs targeting different genes in SCD HSPCs, including *HBB*, *HBG1*, and *BCL11A.*

### LDs and large insertions occur at the CRISPR-Cas9 off-target sites

It has been shown that, compared with WT Cas9, the use of HiFi Cas9 can significantly reduce the small INDEL rates at some of the off-target sites while having a comparable level of small INDEL at the *HBB* on-target cut site (*7*). For comparison, we delivered R-66S gRNA complexed with HiFi Cas9 and WT Cas9, measured the on- and off-target rates, respectively, and compared LD rates and profiles at the *HBB* on-target site and the known off-target site OT18 (*7*). HiFi Cas9- and WT Cas9-treated samples showed similar LD rates (30.3% versus 31.5%) and intermediate deletion rates (6.2% versus 6.3%) quantified by SMRT-seq with UMI (fig. S25), which were also confirmed by ddPCR-based copy number assay (fig. S26). WT Cas9 showed a higher rate of large insertion than HiFi Cas9 (2.2% versus 1.6%). In WT Cas9-treated sample, 53% (36 of 68) of UMI consensus sequences carrying large insertions (≥50 bp) was mapped to the β-globin locus, and 16% of the inserted sequences was mapped to the off-target site OT18, suggesting that large insertions could arise when the off-target DSBs are repaired. In contrast, in the HiFi Cas9-treated sample, none of the inserted sequences was mapped to the known off-target sites. We quantified the LD rate at the off-target site OT18 in the WT Cas9-treated sample and found 3.9% LDs, while the small INDEL frequency at OT18 is 27.7% (fig. S26). OT18 is located at the untranslated region of the olfactory receptor family 5 subfamily AN member 1 (*OR5AN1*). Because LDs at OT18 can be as large as 3760 bp, the implications of disrupting *OR5AN1* and/or nearby genes in HSPCs need to be further studied.

### LDs in PD-1 targeted primary T cells

To demonstrate that the generation of LDs is common in primary cells, we performed SMRT-seq with UMI for the gRNA targeting a PD-1 locus in T cells. The use of CRISPR-Cas9 gene editing to knock out PD-1 in T cells has been explored to enhance T cell functionality in cancer immunotherapy (*39*). We found 15.1 ± 2.6% LDs, 8.7 ± 0.2% intermediate deletions, and 3.4 ± 0.2% large insertions in PD-1 RNP-treated primary human T cells (Fig. 3H and fig. S27). Our results suggest the need to study the large gene modifications and their functional consequences for a wide range of gRNAs designed not only in engineering T cells but also in other gene editing applications.

### LongAmp-seq assay for detection and quantification of LDs

**Fig. 4.**
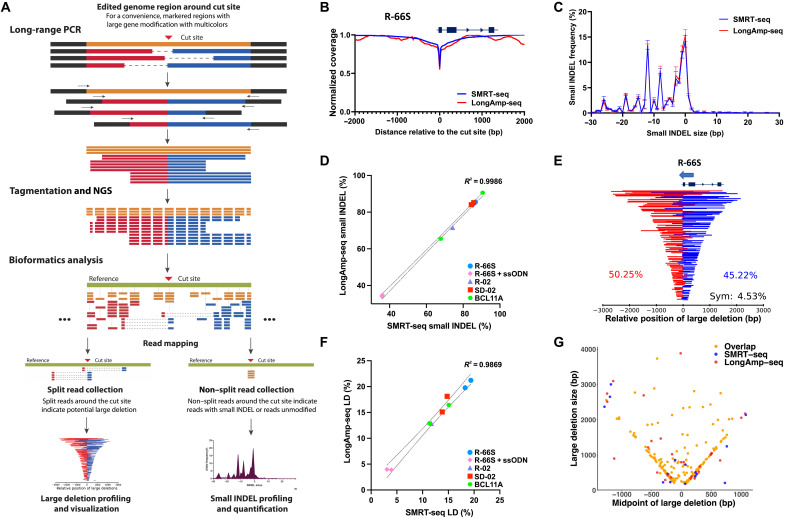
Development and validation of LongAmp-seq for high-throughput detection of LDs. (**A**) Schematics of the LongAmp-seq assay. The LongAmp-seq assay is based on L-R PCR amplification around the Cas9 cut site followed by tagmentation, adaptor extension, and Illumina paired-end deep sequencing. A bioinformatic pipeline was developed for sequence merging, alignment, filtering, and identification of repair outcomes. (**B**) The read coverage pattern of the R-66S RNP-treated SCD HSPCs, normalized by that of the control sample. LongAmp-seq (red) gave similar normalized depletion patterns surrounding the R-66S on-target cut site compared with that obtained by SMRT-seq (blue) (**C**) Small INDEL profile plot from R-66S RNP-treated samples, showing the overlap between SMRT-seq and LongAmp-seq results. (**D**) High correlation between the percentage of small INDEL in unsplit reads quantified by SMRT-seq and LongAmp-seq. (**E**) LongAmp-seq–identified LD patterns were mapped relative to the Cas9 cut site. Representative LongAmp-seq LD profile from R-66S RNP-treated SCD HSPCs from Donor #2. (**F**) High correlation between the percentage of LD quantified by SMRT-seq with UMI and the percentage of LD reads measured by LongAmp-seq. In (D) and (F), biological replicates for each sgRNA were indicated by symbols. *n* = 2 for R-66S RNP, R-66S RNP + ssODN, SD-02 RNP, and BCL11A RNP and *n* = 1 for R-02 RNP. (**G**) The LD patterns identified by SMRT-seq and LongAmp-seq for the R-66S RNP sample were plotted on the basis of the location of midpoint of LDs (*x* axis) and LD sizes (*y* axis). The LDs identified by LongAmp-seq had a high level of overlap (96%) with that by SMRT-seq.

SMRT-seq requires specialized sample preparation and is available only at a core facility, with weeks to months of turnaround time. To enable in-house high-throughput analysis of gene editing outcomes, we developed the LongAmp-seq assay based on L-R PCR amplification, followed by tagmentation, adaptor extension, and Illumina paired-end deep sequencing (Fig. 4A). We developed a bioinformatics pipeline for sequence merging, alignment, filtering, and identification of repair outcomes, including (i) unmodified or small INDELs and (ii) LDs (≥200 bp) (fig. S28). LongAmp-seq–generated reads containing unmodified or small INDEL were analyzed by CRISPResso2 to quantify the small INDEL profile (*30*). Similar to benchmarking SMRT-seq with UMI (fig. S11), we used the same synthetic DNA library containing nine templates with different artificial LDs and quantified the percentages of templates 1 to 9 in the pooled plasmid sample by LongAmp-seq. As shown in fig. S29A, LongAmp-seq gave 70.2% of template 9 in the PCR3 sample, decreased from that quantified by ddPCR (79.9%) in the original template sample, largely due to having more PCR duplicates of templates 1 to 8 in PCR3 sample compared to that of template 9. The LongAmp-seq correctively identified LD-containing templates 1 to 8 presented in the original DNA library without having false positives (fig. S29B).

To establish the ability of LongAmp-seq in accurately quantifying LDs, the same PCR3 products from edited SCD HSPCs analyzed using SMRT-seq were sequenced by LongAmp-seq, and the results were compared. LongAmp-seq sequencing depth and read numbers after each bioinformatics step are shown in table S7. As shown in Fig. 4B, LongAmp-seq gave similar normalized depletion patterns surrounding the R-66S on-target cut site compared with that obtained by SMRT-seq (Fig. 4B). Shown in Fig. 4C are the small INDEL profiles obtained by SMRT-seq and LongAmp-seq for R-66S RNP-treated samples, indicating a high level of agreement between the two assays. The small INDELs at the *HBB*, *HBG1,* and *BCL11A* targeting loci were also quantified by SMRT-seq and LongAmp-seq assays and showed overlapping small INDEL signatures (fig. S30) and excellent correlation of the small INDEL rates (with coefficient of determination *R*^2^ = 0.9968) (Fig. 4D).

The LongAmp-seq generated similar LD patterns surrounding the *R*-66S on-target cut site compared with that obtained by SMRT-seq (Fig. 4E). The percentage of LDs obtained using LongAmp-seq (quantified as the number of reads containing LDs divided by the total reads) was compared to the LD allele frequency quantified by SMRT-seq using UMI consensus reads and showed excellent correlation (R^2^ = 0.9869) (Fig. 4F); although without UMI-based correction of PCR bias and error, LongAmp-seq gave slightly higher LD rates (fig. S31). We further compared the unique LDs identified by SMRT-seq and LongAmp-seq for the same samples and found that the LD alleles identified by LongAmp-seq have a high level (83 to 92%) of overlap with that by SMRT-seq (Fig. 4G and fig. S32). Together, we have demonstrated that LongAmp-seq could accurately identify small INDEL and LD profiles compared to SMRT-seq with UMI despite the significant differences in library preparation, sequencing, and read processing method.

Although LongAmp-seq is much easier to perform than SMRT-seq and quite accurate in quantifying the rate of LDs, it has some limitations. Because of sequence fragmentation for S-R NGS, LongAmp-seq cannot provide correction to PCR bias using UMI, and it is difficult to distinguish complex local rearrangements within the primer binding site from large insertions. Nevertheless, LongAmp-seq could provide accurate measures of both small INDELs and LDs, thus serving as an in-house and high-throughput tool for the analysis of gene editing outcomes.

### HSCs have higher rate of LD and lower rate of HDR than HSPCs

**Fig. 5.**
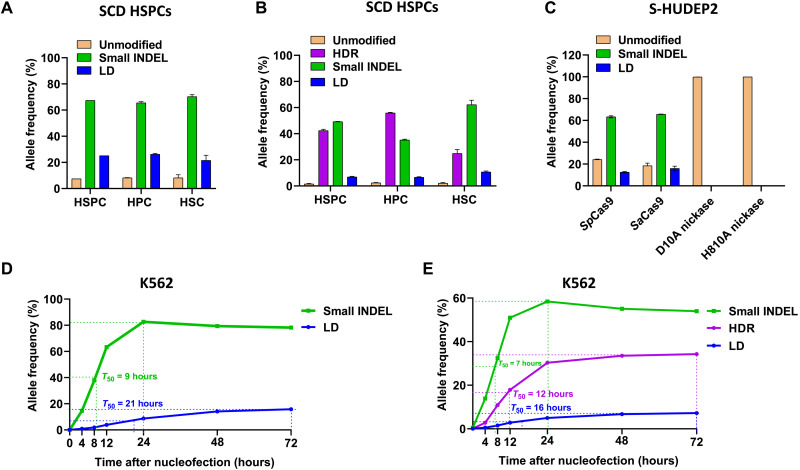
Longevity, editor dependence, and kinetics of LD generation quantified by LongAmp-seq. (**A**) Gene editing outcomes in HSPCs and FACS-sorted HPCs and HSCs. The LD rate induced by R-66S RNP was lower, and the small INDEL rate was higher in HSCs compared to HPCs. (**B**) With both R-66S RNP and ssODN, the sorted HSCs had higher levels of LDs and small INDELs and a lower HDR rate than that of HPCs. (**C**) Comparison of LD rates by different gene editors. RNPs consisting of R-66S gRNA complexed with WT *Sp*Cas9, D10A nickase and H810A nickase, and SA-12S gRNA with *Sa*Cas9 were electroporated into S-HUDEP2 cells. *Sp*Cas9- and *Sa*Cas9-induced DSBs led to high rates of LDs. D10A and H810A nickases did not lead to any LD. (**D** and **E**) Analysis of the dynamics and competition of NHEJ, HDR, and LD generation. (D) In R-66WT RNP-treated K562 cells, small INDELs saturated at 24 hours and LDs saturated at 72 hours after delivery with *T*_50_ (the time to reach half of the maximum modification rate) of 9 and 21 hours, respectively, showing faster repair kinetics by NHEJ than that of LD generation. (**E**) In K562 cells treated with both RNP and ssODN, small INDELs saturated at 24 hours, targeted ssODN insertion by HDR, and LDs saturated at 72 hours after delivery with *T*_50_ of 7, 12, and 16 hours, respectively. HDR effectively outcompetes the repair process that led to LDs, but not NHEJ-led small INDELs.

Because the efficacy of autologous hematopoietic cell therapy depends on the ability to modify HSCs, which have long-term engraftment capability, we applied LongAmp-seq to compare the editing outcome in the HSCs with that in hematopoietic progenitor cells (HPCs) and HSPCs. We quantified the editing outcomes by R-66S RNP with and without ssODN in HSCs (CD34^+^CD38^−^CD45RA^−^CD90^+^) compared to HPCs (CD34^+^CD38^+^) and HSPCs (CD34^+^) (fig. S33). After delivery of RNP or RNP + ssODN, treated SCD HSPCs were recovered in the expansion medium for 2 hours before staining using fluorescently labeled antibodies (CD34, CD38, CD45RA, and CD90) for HSC and HPC sorting via FACS. On the basis of the HSPC immunophenotyping, the percentage of HSCs was 0.5 ± 0.3% (fig. S33). In the RNP-treated sample, we found a lower LD rate in HSCs than HPCs (Fig. 5A). In RNP + ssODN–treated samples, HSCs had higher levels of LDs and small INDELs and a lower HDR rate than that of HPCs (Fig. 5B). We observed enrichment of 1-bp deletion produced by NHEJ accompanied by reduction of MMEJ-led small deletions (notably, −26-bp deletion with CCTGTG 5-bp microhomologies) in HSCs (fig. S34). This is consistent with the results previously reported for the BCL11A gRNA (*28*). Our results suggest that the efficacy in treating SCD using CRISPR-Cas9–based gene correction may be lower than previously reported (*4*, *5*, *7*, *40*) because of the reduced HDR rates as a result of unintended LDs in gene-edited HSCs.

### DNA DSB is required for LD generation

We compared LD rates by different types of editors using LongAmp-seq, including *Staphylococcus aureus* Cas9 (*Sa*Cas9) and Cas9 nickases used in base editing. We delivered into S-HUDEP2 cells with RNPs consisting of R-66S gRNA complexed with *Sp*Cas9, D10A nickase, H810A nickase, and RNP of SA-12S gRNA complexed with *Sa*Cas9. R-66S *Sp* gRNA and SA-12S *Sa* gRNA have mutually permissible Protospacer Adjacent Motif (PAM) sequence (NGGRRT) and generate DSB at the same position in *HBB*. We found that both *Sp*Cas9 and *Sa*Cas9, which generate DSBs, had high and comparable rates of LDs in S-HUDEP2 cells. D10A nickase and H810A nickase complexed with R-66S gRNA did not lead to measurable levels of LDs and small INDELs by LongAmp-seq (Fig. 5C), indicating that single-strand DNA break (nick) does not generate LDs.

### Dynamics and competition of NHEJ, HDR, and LD generation

Although the mechanism(s) of LD generation during DNA DSB repair is not well understood, MMEJ has been implicated as a possibility (*13*, *14*), and the competition of different repair mechanisms likely determines the rates of different gene modifications, including LDs, small INDELs, intermediate deletions, and large insertions. It has been reported recently that the kinetics of HDR falls between NHEJ and MMEJ (*41*). However, the repair kinetics of LDs has not been investigated. We delivered R-66WT RNP with and without the sickle ssODN into K562 cells (a human erythroleukemia cell line), harvested gDNAs at different time points over 3 days after delivery, and analyzed the rates of LDs, NHEJ-led small INDELs, and HDR-mediated ssODN insertion by LongAmp-seq. In the RNP-treated sample, small INDELs saturated at 24 hours and LDs saturated at 72 hours after delivery with *T*_50_ (the time to reach half of the maximum modification rate) of 9 and 21 hours, respectively, showing faster repair kinetics by NHEJ than that of LD generation (Fig. 5D and fig. S35A).

In the sample treated with both RNP and ssODN, small INDELs saturated at 24 hours, ssODN insertion by HDR, and LDs saturated at 72 hours after delivery with *T*_50_ of 7, 12, and 16 hours, respectively (Fig. 5E and fig. S35B). We compared the size distribution of LDs over time and found that the repair of longer LDs was slower than shorter LDs (fig. S36). We found that HDR effectively outcompetes the repair process that led to LDs, but not NHEJ-led small INDELs. Together, after DSB generation, NHEJ is the predominant repair pathway. It is possible that if the DSBs were not repaired promptly by NHEJ and HDR, then cells use the MMEJ pathway to repair the DSBs, resulting in LDs. However, the mechanism(s) responsible for LD generation requires further studies.

### Detection of LDs using Nanopore MinION long-read sequencing

As an alternative to SMRT-seq, Nanopore sequencing can provide large variant detection with long reads (10 to 100 kb) and low costs but has higher error rates than SMRT-seq (*42*). We performed MinION-based Nanopore sequencing with R-66S RNP-treated SCD HSPCs and developed a custom pipeline (fig. S37). Nanopore sequencing gave a read coverage depletion pattern similar to LongAmp-seq (fig. S38) and detected large insertions of up to 1 kb (fig. S39). However, the suboptimal alignments of Nanopore reads (*43*) limit the accuracy of UMI detection and variant calling (*44*). Therefore, despite the advantages of portable and real-time sequencing, Nanopore sequencing might not be the method of choice for detecting and quantifying CRISPR-Cas9 editing–induced large gene modifications.

## DISCUSSION

With recent advances in CRISPR-Cas9–based genome editing methods, high editing efficiencies and reduced off-target effects can be achieved. However, in most gene editing studies, only small INDELs due to the repair of DSBs are quantified using S-R PCR–based sequencing methods. Recent studies have revealed large gene modifications at the Cas9 on-target cut sites using long-read sequencing (*12*, *16*, *21*), ddPCR (*13*), or quantitative genotyping PCR (*17*). However, it remains challenging to quantify the unintended large gene modifications, such as LDs, insertions, and complex local chromosomal rearrangements, because the S-R PCR–based methods cannot detect these large modifications, and no well-established and easy-to-use method exists to serve as the “gold standard.” Therapeutic applications of CRISPR-Cas9–based gene editing necessitate the development of a simple, accurate, and reliable method to analyze gene editing outcomes.

This work provides the first comprehensive analysis of DSB repair outcomes due to Cas9 cutting with different gRNAs in cell lines and primary cells and using different methods, including clonal genotyping, ddPCR copy number quantification, SMRT-seq with UMI, and LongAmp-seq. In carrying out SMRT-seq with UMI, the optimized PCR conditions and library preparation enabled cost-effective sequencing using one SMRTbell template prep kit for up to 24 barcoded samples sequenced on one SMRT cell. We showed that SMRT-seq with UMI provides accurate profiling and quantitation of LDs and large insertions at the Cas9 cut sites by enabling PCR chimera filtering and removal of PCR duplicates based on the identification of UMI consensus sequences. However, SMRT-seq requires sophisticated library preparation and often can only be performed at a core facility. To analyze large gene modifications based on the broadly accessible Illumina short-read sequencing platforms, we developed the LongAmp-seq assay using a short-read sequencer (MiSeq) with high-sequencing coverage and accuracy. The LongAmp-seq assay identified a diverse array of DSB repair outcomes, including small INDELs and LDs, with fairly accurate results comparable to that by SMRT-seq with UMI. Although, in this work, only ex vivo gene-edited cells were analyzed, the same methods (SMRT-seq with UMI and LongAmp-seq) can be used to analyze large gene modifications resulting from in vivo genome editing by using gDNA sample extracted from in vivo–edited tissue and following the L-R PCR and library preparation protocols.

Two important issues remain to be addressed: the mechanism(s) responsible for, and functional consequences of, unintended large gene modifications, especially LDs. Our results suggest that, once a DSB occurs, end protection mechanisms favor a rapid ligation of broken ends via NHEJ that results in small INDELs. It is likely that if the DSB is not repaired quickly, then an end resection process starts at the DSB locus leading to LD. Most LDs were found to be asymmetric about the Cas9 cut site (Figs. 2D and 3, D to G). It has been reported that, upon a DSB generation, Cas9 remains bound to the DNA, leading to asymmetric processing of the exposed DNA ends, thus most of the resection events are asymmetric to the Cas9 cut site (*38*). It is also clear that, in the presence of a corrective DNA donor template such as ssODN, LD rates are significantly reduced (Figs. 2F and 3A), presumably because of the competition between different DNA repair mechanisms (Fig. 5E). However, it remains elusive how the LDs of up to a few thousand base pairs are generated because of DSB at the Cas9 cut site and why LDs can occur with high rates of 10 to 35%.

In general, there are at least five possibilities of having large LDs in the coding region of a gene: (i) disruption of the target gene, (ii) disruption of the target gene and the nearby gene(s), (iii) expression of the target gene resulting in a truncated protein, (iv) expression of the nearby gene(s) resulting in truncated protein(s), and (v) aberrant expression of a nearby gene by putting the otherwise unavailable promoter next to it. Furthermore, large insertions (likely up to a few hundred base pairs) at the cut site may result in (i) misfolding of the protein, (ii) folded protein with extra peptides or domains, and (iii) abolishing of protein folding. The extent and functional consequences of each of these need to be carefully studied. Because the efficacy of autologous hematopoietic cell transplantation depends on the ability to modify genes in HSCs, achieving a high level of therapeutic gene editing with minimal unintended gene modifications is critical. We found that HSCs had a higher LD rate and a lower gene correction rate compared with HPCs (Fig. 5B). Additional work is underway to determine the functional consequences of the unintended large gene modifications at the on-target cut site and their persistence after engraftment of gene-edited SCD HSPCs in the immunodeficient mice.

When performing gene correction of the sickle mutation in *HBB* using an ssODN donor template, without considering more complex gene editing outcomes, the previously reported gene correction rates were overestimated. Furthermore, the possibility of inducing *HBB* KO from intermediate deletions and/or LDs was omitted. It is unclear whether the cells with unintended LDs have equivalent or lower potency than those with the intended edits only. We previously reported significant induction of HbF in R-66S RNP-treated SCD HSPCs (*7*) and hypothesized that the loss of *HBB* alleles would induce compensatory *HBG* expression. A recent report showed that disrupting the *HBB* promoter alleviates promoter competition and activates *HBG* expression (*45*). We found that in R-66S RNP-treated SCD HSPCs, a high percentage of LDs disrupted the *HBB* promoter (Fig. 2D). Therefore, in addition to *HBB* KO or producing protein variants because of in-frame INDELs, LDs may induce HbF expression (*46*–*48*), similar to the naturally occurring HPFH. The 13-nt HPFH deletion in the *HBG1* promoter has been actively pursued as a treatment strategy for β-hemoglobinopathies (*27*). In SD-02 RNP-treated SCD HSPCs, most of the LDs at the *HBG* promoter region resulted in removing the promoter and coding region, which may reduce the number of functional β chains available to form hemoglobin tetramer with α chains, thus exacerbating globin chain imbalance in β-hemoglobinopathies. Furthermore, CRISPR-Cas9–based disruption of the *BCL11A* erythroid enhancer region has been used to induce HbF in human HSCs for treating SCD (*28*, *36*). Our results indicate a high level of diverse LDs of up to 3527 bp at the *BCL11A* enhancer region in SCD HSPCs (Fig. 4F), which could inactivate *BCL11A*, leading to an adverse effect on HSC function and significantly reducing the engraftment potential (*36*). Our findings highlight the importance of accurately quantifying CRISPR-Cas9–induced gene modifications and having a better understanding of the potential consequences of LDs/large insertions, especially in therapeutically relevant cells such as HSPCs and T cells. Furthermore, the current risk assessment of off-target effects is mainly based on small INDEL–induced sequence disruption. Therefore, the consequences of LDs at both on- and off-target sites need to be carefully studied.

Alternative genome editing approaches, such as base editing, may provide a means to avoid LDs/large insertions. We showed that DNA DSB is required for the generation of LDs, consistent with the previous report showing no LDs by base editors in rabbit cell lines (*49*). However, it has been shown that base editors and primer editors can introduce a low level of DSBs, suggesting the possibility of introducing LDs. Furthermore, paired Cas9 D10A nickases have been shown to generate LDs in mouse embryonic stem cells (*13*), and the double nicking strategy in primer editing led to the formation of undesired DSBs in mouse embryos (*50*). A better understanding of the unintended gene modifications by paired base editors and primer editors is needed before their widespread use in therapeutic applications.

## MATERIALS AND METHODS

Human SCD CD34^+^ HSPCs processing and culture were performed as described previously (*7*). Quantification of on-target and off-target activity by S-R NGS was performed as previously described (*7*). Institutional Review Board guidelines were followed with handling human SCD HSPCs.

### HUDEP2 culture

HUDEP2 cells were maintained with StemSpan SFEM medium (STEMCELL Technologies) supplemented with recombinant human stem cell factor (50 ng/ml; PeproTech, 300-07), recombinant human erythropoietin (20 ng/ml; PeproTech, 100-64), dexamethasone (1 μM; Sigma-Aldrich, D8893), and doxycycline hydrochloride (1 μg/ml; Sigma-Aldrich, D3072) (*29*).

### Generation of S-HUDEP2 model

Using nucleofection, we delivered HiFi *Sp*Cas9 protein complexed with R-66 gRNA (*7*) as an RNP complex in conjunction with an ssODN template to introduce the sickle mutation in *HBB* of WT HUDEP-2 cells. Edited HUDEP2 cells were single-cell sorted into multiple 96-well plates and cultured in expansion medium. The clonal genotype was screened using a probe-based ddPCR assay. Thousands of cells from each clone were resuspended in 10 μl of QuickExtract DNA extraction solution (Epicentre) for gDNA extraction, and 1 μl of lysate was used for ddPCR assay. The duplex probe-based ddPCR assay consists of a primer pair amplifying the region around the target site and two probes, a hexachloro-fluorescein-labeled reference (REF) probe binding distant from the target site but still within the amplicon, and a fluorescein amidites-labeled SCD probe binding to modified sickle alleles (GtG). Droplets containing signals from both REF and SCD probes represent sickle alleles, and droplets containing only the REF probe signal represent WT or NHEJ allele. Homozygosity of SCD clones was confirmed using EvaGreen-based ddPCR copy number assay. Sickle cell anemia clones were established and subjected to further analysis.

### Delivery of gene editing reagents to cell lines and primary cells

R-66S, R-02, SD-02, and BCL11A gRNAs sequences were adapted from the literature, and chemically synthesized sgRNAs were ordered from Synthego or IDT. *Sp*Cas9 proteins were purchased from IDT (Alt-R S.p. HiFi Cas9 Nuclease V3, Alt-R S.p. Cas9 Nuclease 3NLS, Alt-R S.p. Cas9 D10A Nickase V3, and Alt-R S.p. Cas9 H840A Nickase V3). *Sa*Cas9 protein was purchased from Synthego (SaCas9; 300 pmol). A total of 2 × 10^5^ to 1 × 10^6^ HUDEP-2, HSPCs (program CA-137, solution P3), T cells (EH115 program, solution P3), or K562 (program FF-120, solution SF) were electroporated on a Lonza 4D-Nucleofector according to the manufacturer’s instructions. A total of 30.5 pmol of HiFi Cas9 protein and 73 pmol of chemically synthesized sgRNAs with or without 100 pmol of ssODN were electroporated. Cells were allowed to recover in expansion medium for >72 hours until the editing is completed.

### Clonal genotyping of R-66S RNP- and ssODN-treated S-HUDEP2 and SCD HSPCs

For S-HUDEP2, edited cells were single-cell sorted into 96-well plates and expanded for 2 weeks. gDNA from each clone was extracted using QuickExtract DNA extraction solution. For SCD HSPCs, edited cells were cultured on semisolid methylcellulose-based medium [MethoCult H4435 Enriched (STEMCELL Technologies, 04445)] for 14 days before being assayed as previously described (*7*). Cells from each colony were resuspended in 20 μl of QuickExtract DNA extraction solution for gDNA extraction and processed for S-R NGS for colony genotyping (*7*). CRISPR-Cas9 genome editing outcomes were analyzed using CRISPResso2 (*30*). To identify clones carrying a LD, the 5.44-kb region containing the on-target cut site at the center was amplified using L-R PCR [LongAmp Hot Start Taq DNA Polymerase; New England Biolabs (NEB), M0534S]. L-R PCR amplicons were run on an agarose gel to check for the gel shift indicating LD. The presence of the LD allele was validated using ddPCR copy number analysis.

### ddPCR to quantify the copy number surrounding the cut site

EvaGreen-based ddPCR assay was used to quantify the copy number near the cut site relative to the nontargeted reference. A primer pair flanking the cut site or targeting at a varying distance away from the cut site and a primer pair targeting the nontargeted reference gene were used (table S1). The reaction mixes were prepared with 15 ng of gDNA templates, 1× ddPCR Supermix (Bio-Rad), 200 nM target primers, and 10 U of Hind III–HF restriction enzyme in each 20 μl of reaction mix. PCR was performed according to the manufacturer’s cycling protocol.

### Tagging gene-edited site with dual UMIs

The first PCR reaction (PCR1) with two amplification cycles was used to target 5- to 6-kb region around the Cas9 cut site and simultaneously tag each template molecule with terminal UMIs using with a tailed primer pair (table S5). The first section of both tailed primers is a synthetic priming site used for downstream amplification, followed by the UMI and target specific sequences. The PCR1 reaction contained 500 ng of gDNA, 200 nM of each tailed primer in 100 μl of reaction (LongAmp Hot Start Taq 2× Master Mix, NEB). The PCR1 program consisted of initial denaturation (2 min at 94°C) and two cycles of denaturation (30 s at 94°C), annealing (30 s at 60°C), and extension (6 min at 65°C). After completion of PCR1, 5 μl of thermolabile exonuclease I (NEB, M0568) was added to the PCR1 reaction and incubated at 37°C for 4 min followed by heat inactivation at 80°C for 1 min to degrade all single-stranded DNA present in the PCR1 mixture. The PCR1 product was purified using SPRIselect (Beckman Coulter, B23317) and eluted in 30 μl of water.

### Amplification and barcoding of UMI-tagged amplicons

PCR2 was used to amplify the UMI-tagged template molecules. The PCR2 reaction contained 5 to 10 μl of UMI-tagged template molecule from PCR1, 200 nM of each universal primers binding to the synthetic priming site (table S5) in 100 μl of reaction (LongAmp Hot Start Taq 2× Master Mix). The PCR2 program consisted of initial denaturation (2 min at 94°C) and 25 cycles of denaturation (15 s at 94°C), annealing (30 s at 60°C), and extension (6 min at 65°C) followed by final extension (5 min at 65°C). The PCR2 product was purified using SPRIselect and eluted in 30 μl of water. In PCR3, barcodes are incorporated by using universal sequences tailed with 16-bp PacBio barcode sequences (Sequal_RSII_96_barcodes_v1). The final barcoded PCR3 amplicon product contains the same barcode sequence on both ends. The PCR3 reaction contained 5 to 10 μl of UMI-tagged template molecule from PCR2, 200 nM of each barcoded universal primer in 100 μl of reaction (LongAmp Hot Start Taq 2× Master Mix). The PCR1 program consisted of initial denaturation (2 min at 94°C) and 5 to 10 cycles of denaturation (15 s at 94°C), annealing (30 s at 60°C), and extension (6 min at 65°C) followed by the final extension (5 min at 65°C). The minimum cycle number was used to obtain sufficient PCR product (>100 ng) for library preparation. The PCR3 product was purified using SPRIselect and eluted in 30 μl of water. For PCR1 to PCR3, a large reaction volume was used to minimize the risk of overamplification. The same PCR conditions described above were used for all genomic loci (*HBB*, *HBG1*, *BCL11A*, and *PD1*).

### Library preparation for SMRT-seq

One hundred nanograms of the UMI-tagged and barcoded amplicon from PCR3 was pooled, and a total of 1 μg of the pooled amplicons was used for PacBio library preparation, which consists of DNA damage repair, end repair/A-tail, SMRTbell adaptor ligation (SMRTbell Express Template Prep Kit 2.0), nuclease treatment (SMRTbell Enzyme Clean Up Kit), and AMPure bead purification following the standard protocol. The SMRTbell library was sequenced on a PacBio Sequel II 8M flow cell in CCS mode following the standard protocol with 1 hour of preextension and 30 hours of collection time (PacBio). The PacBio subreads were converted to HiFi reads, and Q20 CCS reads were used for analysis.

### Pipeline design for SMRT-seq data analysis

The longread_umi pipeline described by Karst *et al.* (*26*) was adapted to generate UMI consensus sequences from demultiplexed PacBio CCS reads. The consensus sequence for each UMI bin (clustered UMI pair) was generated by multiple rounds of polishing using the binned raw reads (fig. S10). The UMI consensus sequences were then used to call variants using a bioinformatics toolkit we developed called LV_caller. UMI consensus sequences were first aligned to the reference amplicon sequence of interest using Minimap2 (*51*) with spliced long read preset (minimap2 -ax splice). The unaligned sequences will be removed as invalidated ones. The mapped UMI consensus sequences were then processed and categorized into four groups on the basis of the alignment result (SAM format): (i) unmodified alleles and those with small INDELs, (ii) Intermediate deletion of 50 to 200 bp, (iii) LD ≥ 200 bp, and (iv) large insertion ≥ 50 bp, and the sequences that contain both large insertion and deletion were put in subgroup of (iv). To identify LD patterns, a clustering process with ±10 bp of deletion size tolerance and ±10 bp of deletion start position tolerance was applied to account for potential shifts in read mapping introduced by sequencing/alignment. UMI consensus sequences carrying LD within the tolerance window in sequence alignment were taken as UMI consensus sequences carrying the same LD pattern (the combination of size and location). Sequences containing large insertions (≥50 bp) were extracted and aligned against the hg19 genome using a pairwise alignment tool, BLAT (*31*).

### Library preparation of LongAmp-seq

One hundred nanograms of L-R PCR products were used for LongAmp-seq library preparation, which consists of on-bead tagmentation, posttagmentation clean up, 5-cycle PCR to add index adaptors, double-sided bead purification, library pooling, and quantification according to the Nextera DNA Flex Library Prep Reference Guide [Nextera DNA Flex Library Prep Kit (Illumina, 20018704) and Nextera DNA CD Indexes (Illumina, 20018707)]. Up to 24 dual-indexed samples were pooled and sequenced on MiSeq at 20 pM final loading concentration using the MiSeq Reagent Kit v3 (600 cycles), which generated an average of 611,836 raw reads per sample. After merging paired-end reads by FLASH (fast length adjustment of short reads), allowing a maximum of 600 bp of merged read length, average 307,881 reads were generated with 50% proportion of combined pairs. One fragmented short-read spanning the CRISPR-Cas9 cut site is expected from each L-R PCR product. After filtering out reads not spanning the cut site, we retained 7% reads for *HBB* amplicon (5189 bp), 5.5% reads for *HBG1* amplicon (6578 bp), and 8.6% reads from *BCL11A* amplicon (4351 bp). An average of 22,881 reads spanning the Cas9 cut site was used for LD_caller (table S7).

### Pipeline design for LongAmp-seq data analysis

The raw sequencing data from Illumina MiSeq were demultiplexed by bcl2fastq and merged using FLASH (*52*). Merged reads were aligned to the reference sequence using Burrows-Wheeler Alignment (BWA)-Maximal Exact Match (MEM) (*53*), and the read coverage patterns were extracted by igvtools (*54*). The reads that were not spanning the cut site were filtered out with SAMtools (*55*). The split reads were identified using BEDtools (*56*) and further processed to breakpoint-based variant calling, while the small INDEL patterns were generated by CRISPResso2 (*30*) using the unsplit reads.

### Construction and validation of synthetic standard with the predetermined allele frequency

HBB sequences were PCR-amplified from gDNA and cloned into the pUC19 backbone to generate a plasmid template with an unmodified HBB allele. LD of eight different sizes was introduced by site-directed mutagenesis into the unmodified HBB plasmid (Q5 Site-Directed Mutagenesis Kit, E0554S). Each synthetic DNA template was assigned 6-bp allele-specific barcode at the 5′ end, which was later used to verify the accuracy of LD variant calling. Sanger sequencing verified a total of nine plasmid templates with allele-specific barcodes. The nine plasmid templates were pooled at a predetermined molar ratio. The pooled plasmid was linearized by restriction enzyme digestion outside of HBB sequences. The relative percentages of templates 1 to 9 in the pooled plasmid standard were quantified by duplex probe-based ddPCR using template barcode–specific primer pairs and a reference primer pair binding to all templates. The linearized pooled synthetic plasmid was used as a template for the three-step L-R PCR (2× PCR1, 25× PCR2, and 10× PCR3) using a tailed M13 primer pair to generate UMI-tagged and barcoded PCR3 products. Barcoded PCR3 product (amplicon size of 1221 to 5672 bp) was library prepared and sequenced by SMRT-seq and LongAmp-seq.

### Nanopore MinION long-read sequencing

L-R amplification products generated from the LongAmp-Seq were library prepared using the ONT Ligation Sequencing Kit (SQK-LSK109) and Native Barcoding Expansion 1-12 (PCR-free) kit (EXP-NBD104) following standard protocols. One microgram of each sample was brought to a final volume of 49 μl with nuclease-free water. DNA ends were repaired using the NEBNext FFPE DNA Repair and ultra II end-prep kits according to ONT protocol. Samples were incubated at 20°C for 5 min and 65°C for 5 min, cleaned with 60 μl of AMPure bead, and eluted in 25 μl of nuclease-free water. A maximum of 500 ng of each cleaned sample was used for native barcode ligation according to the barcoding kit specifications. Adapter mix II ligation was performed according to standard ONT protocol using the NEBNext Quick Ligation Reaction Buffer (5×) and Quick T4 DNA Ligase followed by AMPure bead cleanup using the Long Fragment Buffer for washing and eluted in 15 μl of nuclease-free water. Samples were quantified by the high-sensitivity Qubit assay, normalized by molar concentration, and pooled. SpotON flow cell priming and loading were performed on the basis of the standard protocol. Fast5 sequence files were processed into Fastqs using Guppy Basecaller. We first used CoNvex Gap-cost alignMents for Long Reads (NGLMR) (*57*) to map all long reads to hg19, and the reads that mapped to the *HBB* region were analyzed for deletions and insertions calling. The reads that could not be mapped by NGMLR were further aligned by BWA-MEM (*53*) and filtered by SAMtools (*55*) to include the chimeric reads carrying the potential LD. The insertion profile was from NGMLR calling, and the LD profile included both NGMLR-called reads and BWA-MEM–identified reads.

## Notes

View/request a protocol for this paper from *Bio-protocol*.
