# Supplementary material for: Comprehensive analysis and accurate quantification of unintended large gene modifications induced by CRISPR-Cas9 gene editing
Source: Sci Adv. 2022 Oct 21;8(42):eabo7676. doi: 10.1126/sciadv.abo7676 (PMC9586483; doi:10.1126/sciadv.abo7676)
Supplement: Supplementary file 1 — Tables S1 to S7 Figs. S1 to S39 [file sciadv.abo7676_sm.pdf]

Science Advances
AAAS

Supplementary Materials for

Comprehensive analysis and accurate quantification of unintended large gene modifications induced by CRISPR-Cas9 gene editing

So Hyun Park *et al.*

Corresponding author: Gang Bao, gang.bao@rice.edu

Sci. Adv. **8**, eabo7676 (2022)
DOI: 10.1126/sciadv.abo7676

This PDF file includes:

Tables S1 to S7
Figs. S1 to S39

Supplementary Information

Supplementary Tables

Supplementary Table S1. List of gRNAs. R-66S and R-02 gRNAs target the first exon of *HBB* with varying proximity to the SCD mutation site. SD-02 gRNA introduces 13-nt HPFH deletion at high frequency in the *HBG1* and *HBG2* promoters for the reactivation of HbF. *BCL11A* gRNA targets the GATA1 binding site at the *BCL11A* erythroid enhancer for the reduction of *BCL11A* expression and induction of HbF.

gRNA	Target	gRNA sequence (PAM)	References
R-66S	HBB	**GTAACGGCAGACTTCTCCACAGG**	Park (7)
R-02	HBB	**CTTGCCCCACAGGGCAGTAACGG**	Hoban (6), DeWitt (5), Dever (4)
SD-02	HBG	**CTTGTCAAGGCTATTGGTCAAGG**	Humbert (27)
BCL11A	BCL11A	**CTAACAGTTGCTTTTATCACAGG**	Wu (28)

Supplementary Table S2. Identification of colony genotype after *HBB* gene-editing in S-HUDEP2 by S-R NGS, L-R NGS with gel shift assay and ddPCR.

Supplementary Table S3. Identification of colony genotype after R-66S RNP delivery in SCD HSPCs by S-R NGS, L-R NGS with gel shift assay and ddPCR.

Supplementary Table S4. Identification of colony genotype after R-66S RNP and ssODN delivery in SCD HSPCs by S-R NGS, L-R NGS with gel shift assay and ddPCR.

Supplementary Table S5. List of primer sequences

Supplementary Table S6. Details of large insertions in SCD HSPCs treated by R-66S RNP

Supplementary Table S7. LongAmp-seq sequencing depth and read numbers

Supplementary Figures

Figure S1

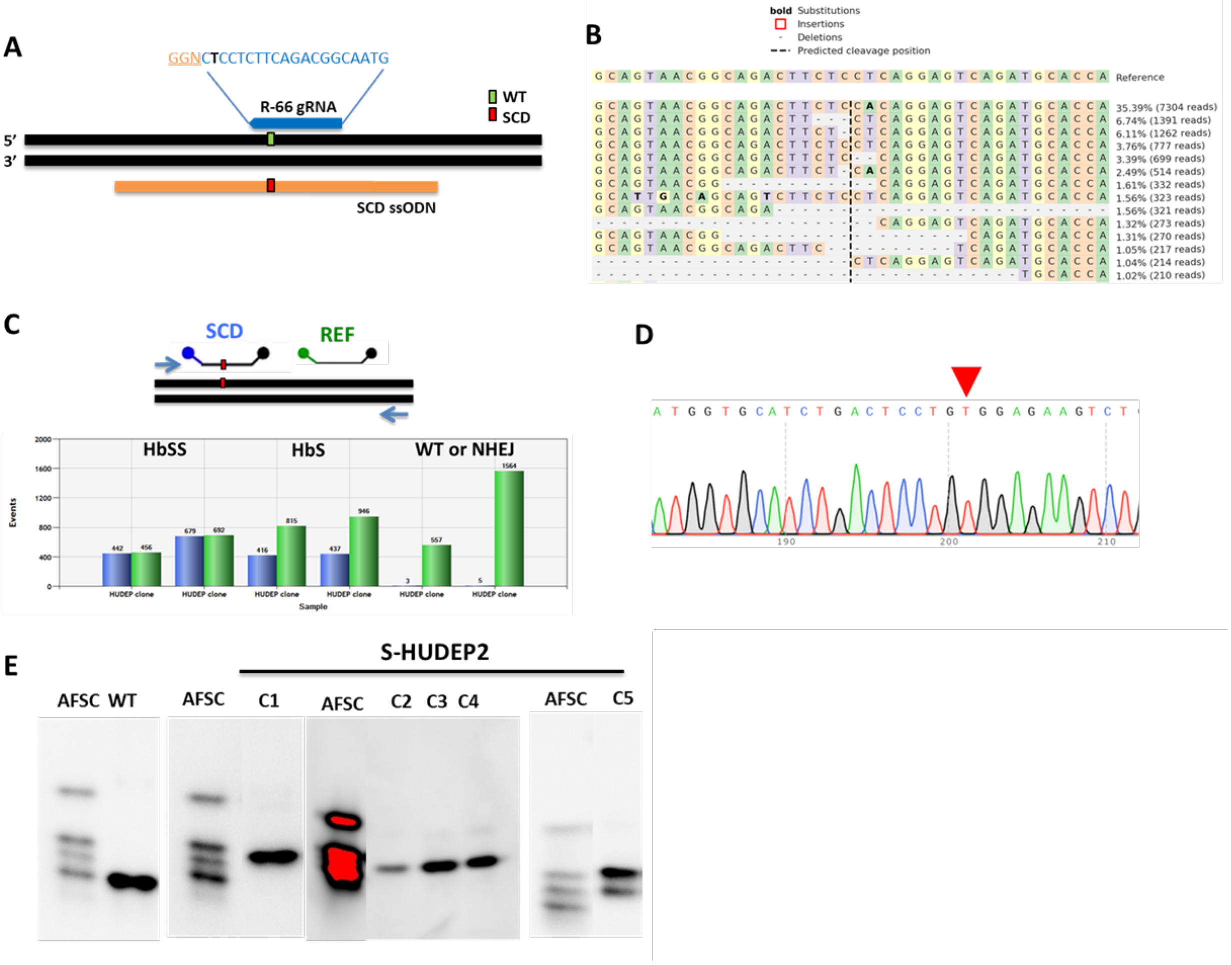

Figure S1. Generation of S-HUDEP2 cell model. (**A**) Schematic of gRNA and ssODN design near the SCD locus (with the green or red box showing the location of sickle mutation). High-Fidelity (HiFi) *Sp*Cas9 and WT *HBB*-targeting R-66 gRNA as RNP was delivered along with ssODN to introduce the sickle mutation into WT HUDEP2. The R-66 gRNA sequence is shown in blue (with the WT base T in black), and the PAM sequence is shown in orange and underlined. The SCD ssODN (shown in orange with the SCD mutation in red) is symmetric with respect to the R-66 cut site and complementary to the gRNA non-target strand. (**B**) Visualization of the frequent allele changes (>1%) around the Cas9 cut site in HiFi *Sp*Cas9/R-66 gRNA and SCD ssODN treated WT HUDEP2. Substitutions are shown in bold font. Inserted sequences are highlighted with red rectangles, and deletions are indicated as horizontal dashed lines. A vertical dashed line indicates the predicted cleavage position of R-66 gRNA. Deep sequencing data were analyzed by CRISPResso2 (30). (**C**) Design of probe-based ddPCR assay and representative ddPCR event counts for different clonal genotypes. The probe-based ddPCR assay consists of a primer pair and three probes, a *HEX* reference (REF) probe binding distant from the target site but still within the amplicon, and a FAM SCD probe binding to modified sickle alleles (GtG). Droplets containing signals from both REF and SCD probes represent sickle alleles, and droplets containing only the REF probe signal represent WT or NHEJ allele. Homozygous SCD clones have the same number of HEX and FAM events. (**D**) Sanger sequencing chromatogram of the S-HUDEP2 clone. The SCD mutation is indicated with a red arrow. (**E**) Representative hemoglobin

native PAGE gel and HPLC showing HbA production in WT HUDEP2 and HbS production in S-HUDEP2 clones. S-HUDEP2 clones with exclusive HbS production. Hemoglobin AFSC control contains mix of HbA, HbF, HbS and HbC (bottom-up).

Figure S2

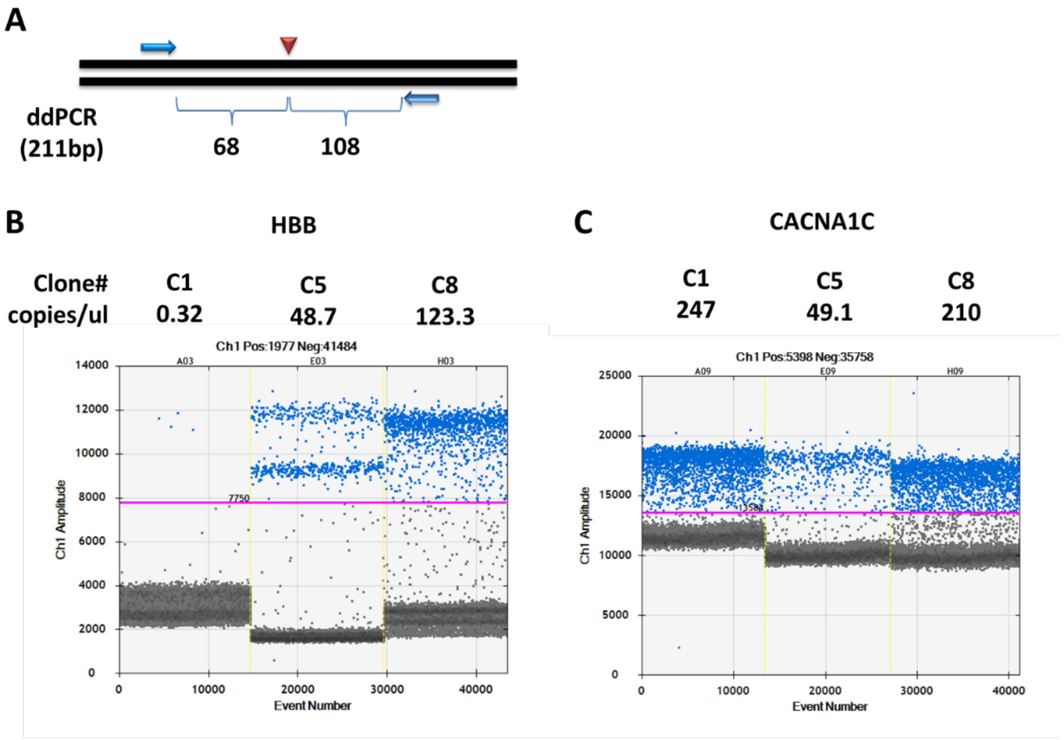

D

	HBB copies/ul	CACNA1C copies/ul	%HBB/REF	%LD	Genotype
C1	0.32	247	0.1	100	LD/LD
C5	48.7	49.1	99.2	0	SI/SI
C8	123.3	210	58.7	50	SI/LD

Figure S2. Design and validation of ddPCR assay for the quantification of the *HBB* copy number. The EvaGreen-based ddPCR *HBB* copy number assay consists of two separate PCR reactions: a primer pair targeting *HBB* on chr11 and a primer pair targeting reference gene on chr12. Alleles containing large deletions or chromosomal rearrangements that remove primer binding site/s cannot produce *HBB* signals. Therefore, we checked the copy number of *HBB* relative to a reference gene (*CACNA1C*). (**A**) An *HBB* forward primer binds 68bp upstream of the cut-site, and a reverse primer binds 100bp downstream of the cut-site. (**B**) ddPCR amplitude plot for 3 representative clones showing droplets containing signals from *HBB* amplification. (**C**) ddPCR amplitude plot for 3 representative clones showing droplets containing signals from *CACNA1C* amplification. (**D**) The copy number of *HBB* was normalized by the copy number of *CACNA1C* to figure out the number of alleles (0, 1 or 2) carrying large deletion (LD) in each clone. For clone 1, the absence of the *HBB* copy number indicates two LD alleles. For clone 5, 100% of *HBB* copy number indicates the absence of LD. For clone 8, a 50% *HBB* copy number indicates one LD and one small INDEL (SI).

Figure S3

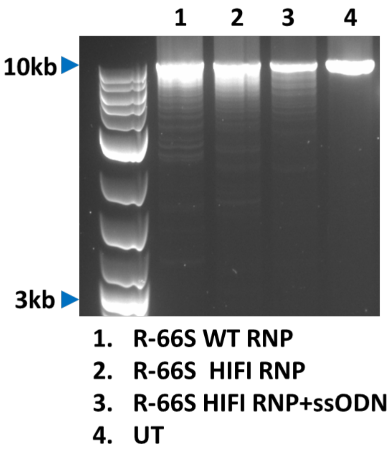

Figure S3. Size-shifted bands in 10kb L-R PCR. Alleles containing LD generate smaller long-range PCR (L-R PCR) products as shown in size shifted bands in 1. R-66S WT-Cas9 RNP, 2. R-66S HiFi-Cas9 RNP and 3. R-66S HiFi-Cas9 RNP + ssODN treated samples, compared to 4. Untreated.

Figure S4

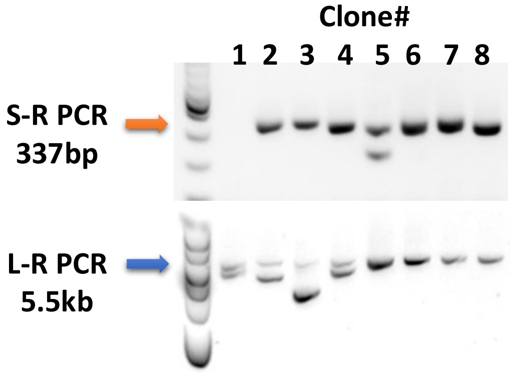

Figure S4. Agarose gel image showing the S-R PCR and L-R PCR bands of 8 representative single-cell clones. The short-range PCR (S-R PCR) amplify the 300 bp region spanning the cut-site, and alleles containing LD would fail to generate PCR product. The L-R PCR amplify the 5.5 kb region spanning the cut-site, and alleles containing a LD within the long-range PCR primer binding sites generate smaller PCR products.

Figure S5

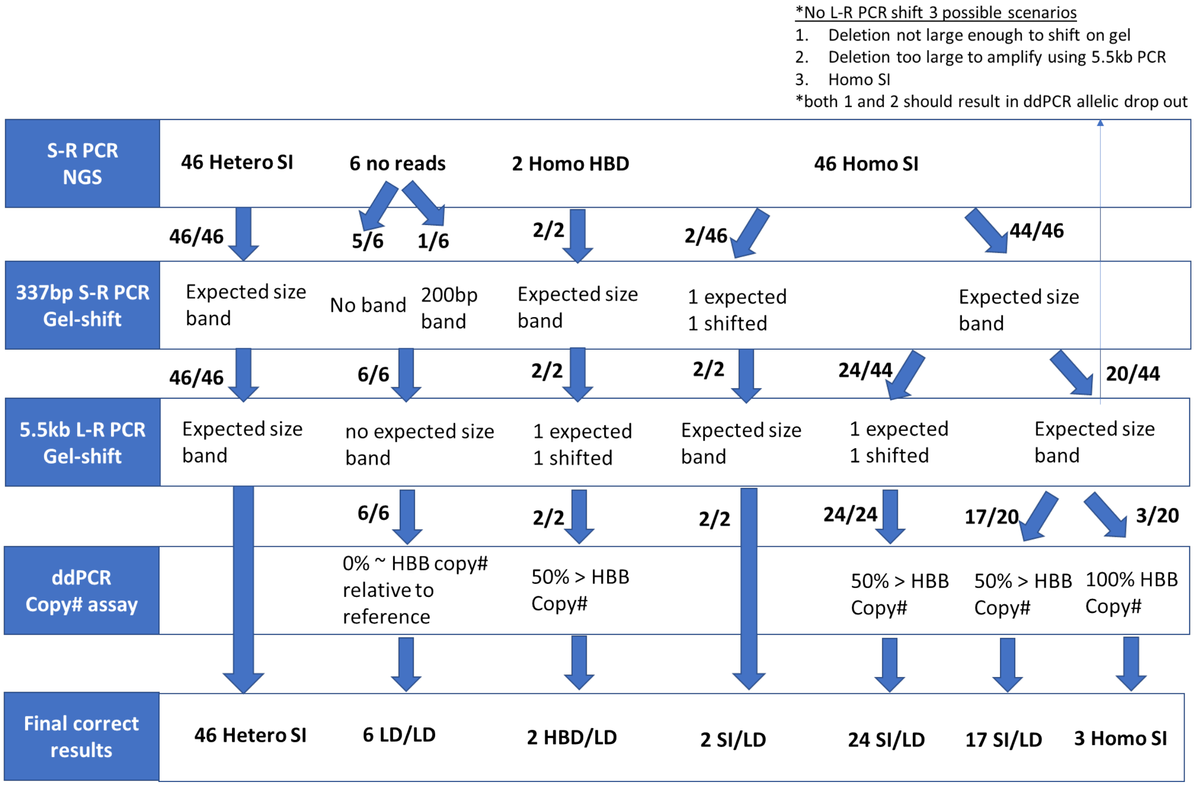

Figure S5. The schematic showing how the colony genotype was determined based on NGS, L-R PCR gel shift, and ddPCR copy number assay.

Figure S6

SI=Small INDEL
LD=Large Deletion

Clone#	Genotype
1	LD/LD
2	SI/LD
3	SI/LD
4	SI/LD
5	SI/SI
6	SI/SI
7	SI/SI
8	SI/LD

Figure S6. Three assays determined the clonal genotype of 8 clones shown in Fig. S4. For clone 1, the absence of S-R PCR band and two size-shifted L-R PCR bands suggest LD/LD genotype. For clones 2-4, one expected sized band and one downward shifted L-R band suggests SI/LD genotype. Although clone 8 does not have a size-shifted L-R PCR band, this clone has SI/LD genotype based on the ddPCR allelic drop-off assay.

Figure S7

A

Genotype	Hetero SI	Homo SI	SI/LD	LD/LD	HBD/ HBD	HBD/ LD	No band	Total clones
# of clones by S-R NGS	46	46	-	-	2	-	6	100
# of clones by 3 assays	46	4	42	6	0	2	0	

B

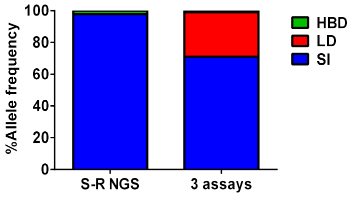

Figure S7. The comparison of genotype results obtained using S-R NGS and combination of three assays respectively for 100 single-cell clones from R-66S RNP treated S-HUDEP2 cells. The genotype of each single-cell clone was identified by S-R NGS and two additional complementary detection methods to account for the drop-out of large deletion alleles: Long-Range (L-R) PCR followed by gel shift assay and ddPCR-based *HBB* copy number quantification. (**A**) With S-R NGS, 46 clones were found to have heterozygous small INDEL (SI) and 46 clones with homozygous SI (**Supplementary Table S2**). We previously showed that Cas9 cutting induced DNA double-strand breaks (DSB) in *HBB* could be repaired using the homologous sequences from the δ-globin gene (*HBD*) as an endogenous template, resulting in SCD mutation correction. We found that two clones had homozygous SCD mutation correction mediated by *HBD* gene conversion, and six clones failed to amplify S-R PCR products. As expected, no genotype with large deletion (LD) could be identified by S-R PCR. In contrast, the combination of three assays (S-R NGS, L-R PCR and ddPCR) gave different genotypes of the single-cell clones: out of the 46 clones identified as homozygous SI genotype by S-R NGS, only 4 clones were indeed homozygous SI while 42 clones carried LD. We found that the six clones that failed to amplify S-R PCR product all had LD/LD genotype, and the two clones with *HBD* conversion had LD. As shown in (**A**), 28% large deletion alleles (i.e., 56 alleles) occurred in 50% of clones (50 clones), which is consistent with the Hardy-Weinberg predictions (48%). Therefore, the use of S-R NGS significantly overestimated the percentage of SI alleles (97.8%) compared with that identified using the combination of three assays (71%). More significantly, our results showed that 50% of the single-cell clones had LD in at least one allele, which caused a significant reduction of *HBB* copy numbers in gene-edited S-HUDEP2 cells as quantified by ddPCR. (**B**) S-R NGS significantly overestimated the percentage of SI alleles; 97.8% SI and 2.1% *HBD* alleles were incorrectly identified by S-R NGS compared to 71% SI, 28% LD, and 1% *HBD* alleles identified by the combination of three essays.

Figure S8

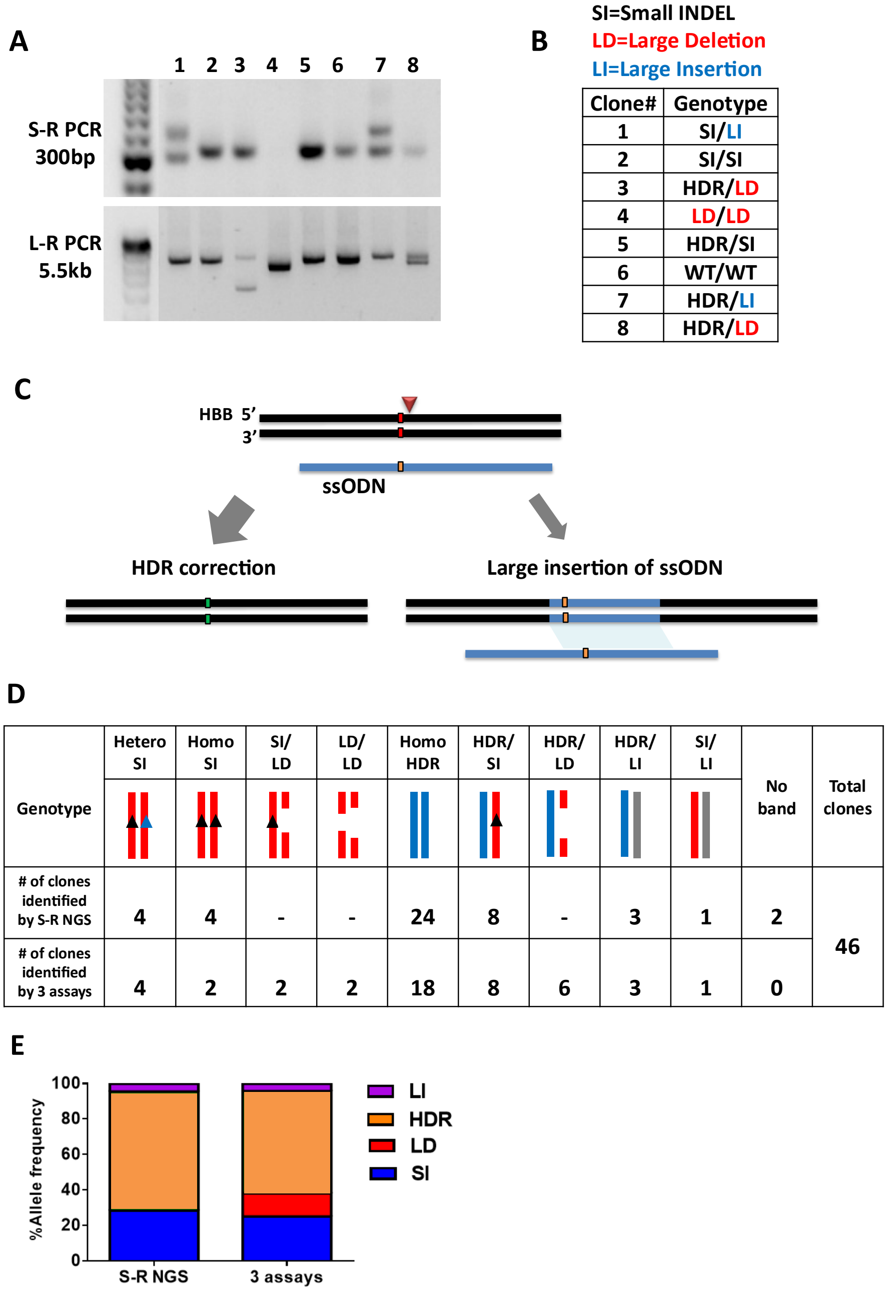

Figure S8. Clonal genotypes of R-66S RNP and corrective ssODN donor treated S-HUDEP2. S-HUDEP2 treated with R-66S HiFi RNP and corrective ssODN was assayed as single cell clones using Illumina short-read sequencing (NGS), L-R PCR, and ddPCR copy number assays for clonal genotypes. (**A**) Representative agarose gel image showing the S-R PCR and L-R PCR products of 8 clones. Clone 1 contains a combination of an upward size-shifted and a downward size-shifted banding pattern, suggesting an approximately 100bp large insertion and a small INDEL (LI/SI genotype). Although the insertion allele was amplified by S-R PCR, the NGS analysis failed to detect this particular allele, providing a genotype of near 100% 26bp deletion. In order to capture this large insertion, the alignment threshold was reduced from >80% to >70%. NGS analysis under the adjusted alignment conditions indicated 35% of reads corresponded to a

66 bp insertion and the remaining 65% of reads the previously detected -26 bp deletion. While this clonal genotype of heterozygotes should yield a 50/50 representation of each allele, the +66 bp/-26 bp size differences between these alleles create a significant PCR bias towards smaller amplicons. The insertion alleles for clone 7 were similarly lost during the standard alignment step and regained upon reducing the reference alignment threshold. This clone was then genotyped at 45% large insertion (+90 bp) to 55% HDR (LI/HDR genotype). For clone 4, the absence of S-R PCR band and size-shifted L-R PCR bands suggest two large deletion events (LD/LD genotype). (**B**) Table of genotype for 8 clones based on (**A**). (**C**) Out of 8 clones carrying large insertion, 7 clones harbor partial ssODN insertion, suggesting incorporation of ssODN at the DSBs instead of HDR-mediated precise SCD mutation correction. (**D**) The combination of three assays showed the correct genotype results: out of 24 clones previously identified as homozygous HDR genotype by S-R NGS, 18 clones were homozygous HDR while 8 clones carried large deletion. Two clones that failed to amplify S-R PCR products have LD/LD genotype. Two clones with homozygous SI during S-R PCR were, in fact, carriers for large deletion. (**E**) 64% HDR incorrectly identified by S-R NGS compared to 54% HDR alleles identified by three assays.

Figure S9

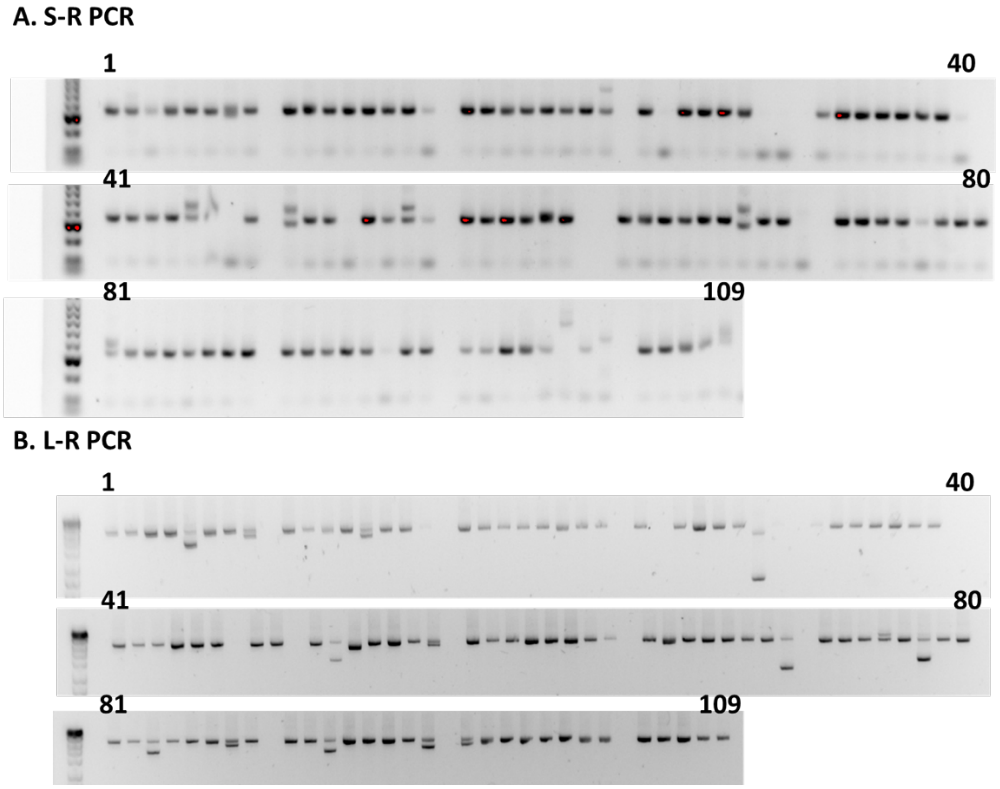

Figure S9. R-66S RNP and ssODN treated S-HUDEP2 clonal genotype gel images. (**A**) 300 bp S-R PCR amplicons from 109 clones. (**B**) 5.5kb L-R PCR amplicons from 109 clones.

Figure S10

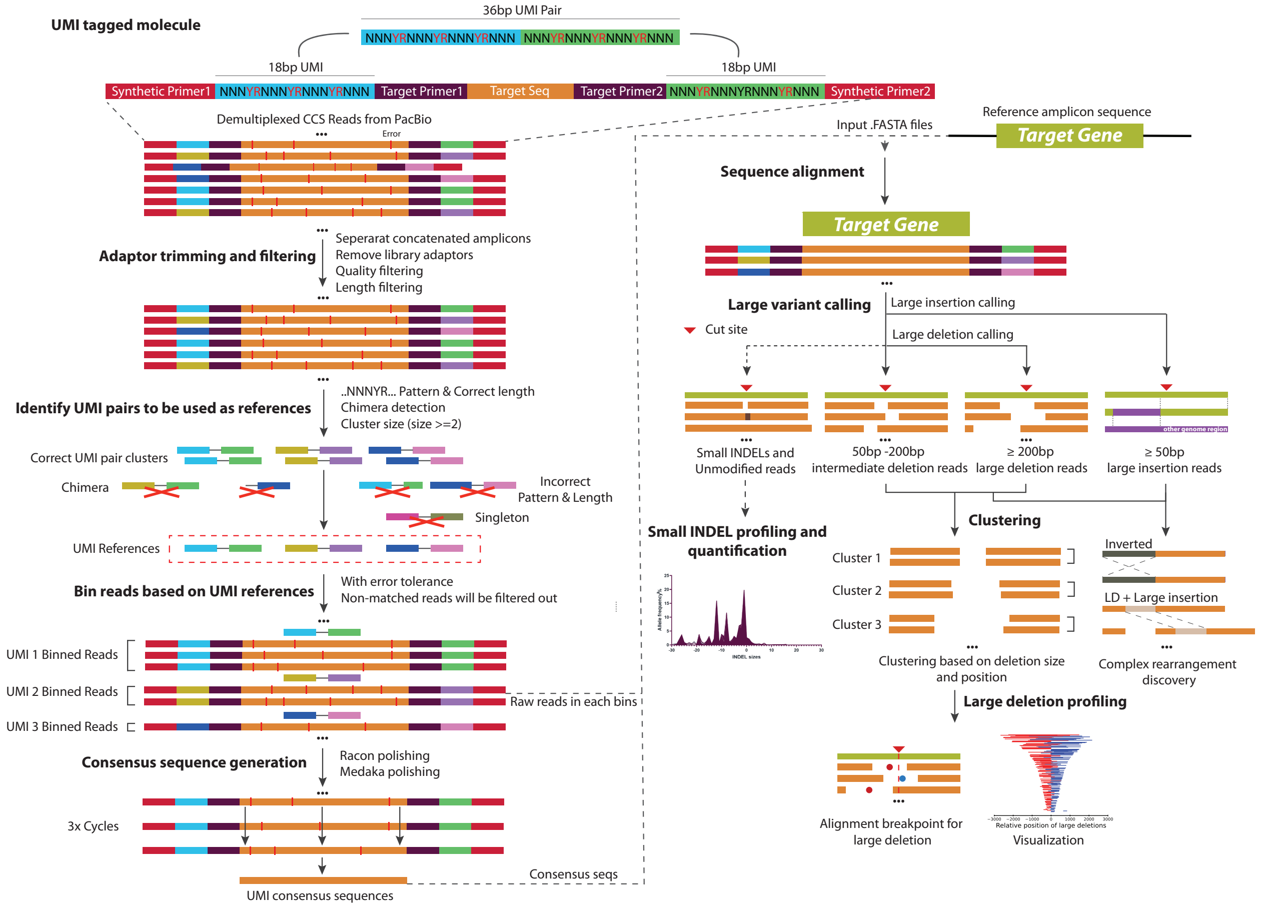

Figure S10. Schematics of SMRT-seq with UMI data processing. (A) longread_umi pipeline (26). The demultiplexed HiFi CCS reads from SMRT-seq were trimmed and filtered with quality control (≤15% expected error) and length control. UMI pairs were extracted and filtered based on the designated UMI pattern (NNNYRNNNYRNNNYRNNN) and length (=18 bp) for each UMI. Identical and highly matched (with some tolerance for UMI PCR/sequencing error) UMI pairs were clustered, and CCS reads with one UMI (singleton) were discarded. Raw CCS reads were binned together based on alignment with each clustered UMI pair. After binning, UMI consensus sequences were generated with Racon polishing and Medaka polishing. (B) Large variants caller pipeline (LV_caller). UMI consensus sequences were taken as the input data set, and the sequence of the target gene (e.g. *HBB*) was taken as the reference sequence (all the file formats are in fasta). Minimap2 was used to align UMI consensus sequences to the reference sequence. The UMI consensus sequences were then categorized into four groups: (i) Unmodified alleles and those with small INDELs, (ii) intermediate deletions of 50-200 bp, (iii) LDs of >= 200 bp, and (iv) large insertions of >= 50 bp (including those simultaneously have intermediate deletions or large deletions). Small INDELs were profiled with INDEL size and allele frequency distribution; large deletions were profiled based on their alignment breakpoint and complex local rearrangements; large insertions were aligned against hg19 using BLAT and annotated chromosome mapping. To further profile deletions patterns, large deletions with similar sizes (+/- 10 bp) and starting positions (+/- 10 bp) were clustered.

Figure S11

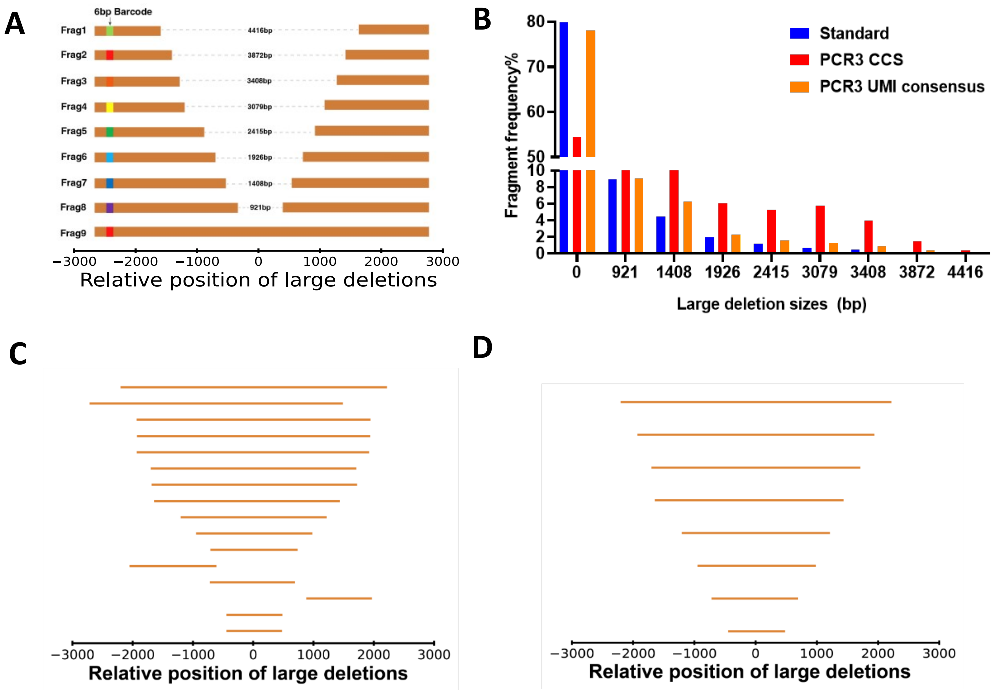

Figure S11. Benchmark the SMRT-seq with UMI using known mixtures of allelic variants. (**A**) We constructed a synthetic DNA standard consisting of a wildtype *HBB* sequence of 5490 bp (Template 9) and Templates 1-8 with artificial LDs of 8 different sizes (4416 bp, 3872 bp, 3408 bp, 3079 bp, 2415 bp, 1926 bp, 1408 bp and 921 bp, respectively). Each DNA template was assigned a 6 bp allele-specific barcode at the 5' end to verify the accuracy of LD variant calling. The nine plasmid templates were linearized and pooled with specific molar ratios, with 80% Template 9 and 20% of Templates 1-8 combined. (**B**) The relative percentages of Templates 1-9 in the pooled plasmid standard were quantified by duplex probe-based ddPCR using barcode-specific primers and reference primers. The synthetic DNA library was then used as the standard for a 3-step L-R PCR to generate UMI-tagged and barcoded PCR3 products, sequenced using SMRT-seq to quantify the percentages of Templates 1-9. Based on the aligned CCS reads, Template 9 in PCR3 product was 54.38%, significantly decreased from 79.9% quantified by ddPCR in the original sample as standard, largely due to PCR errors. (**C**) Using the aligned CCS reads, LDs of the same start position and size were clustered together to identify unique LD patterns, and each large deletion pattern was mapped relative to the Cas9 cut-site. The CCS reads contain false-positive LDs different from that in Templates 1-8. (**D**) LDs identified using UMI consensus reads were mapped relative to the Cas9 cut-site. Only Templates 1-8 were identified, demonstrating SMRT-seq with UMI can accurately quantify LDs without false positives.

Figure S12

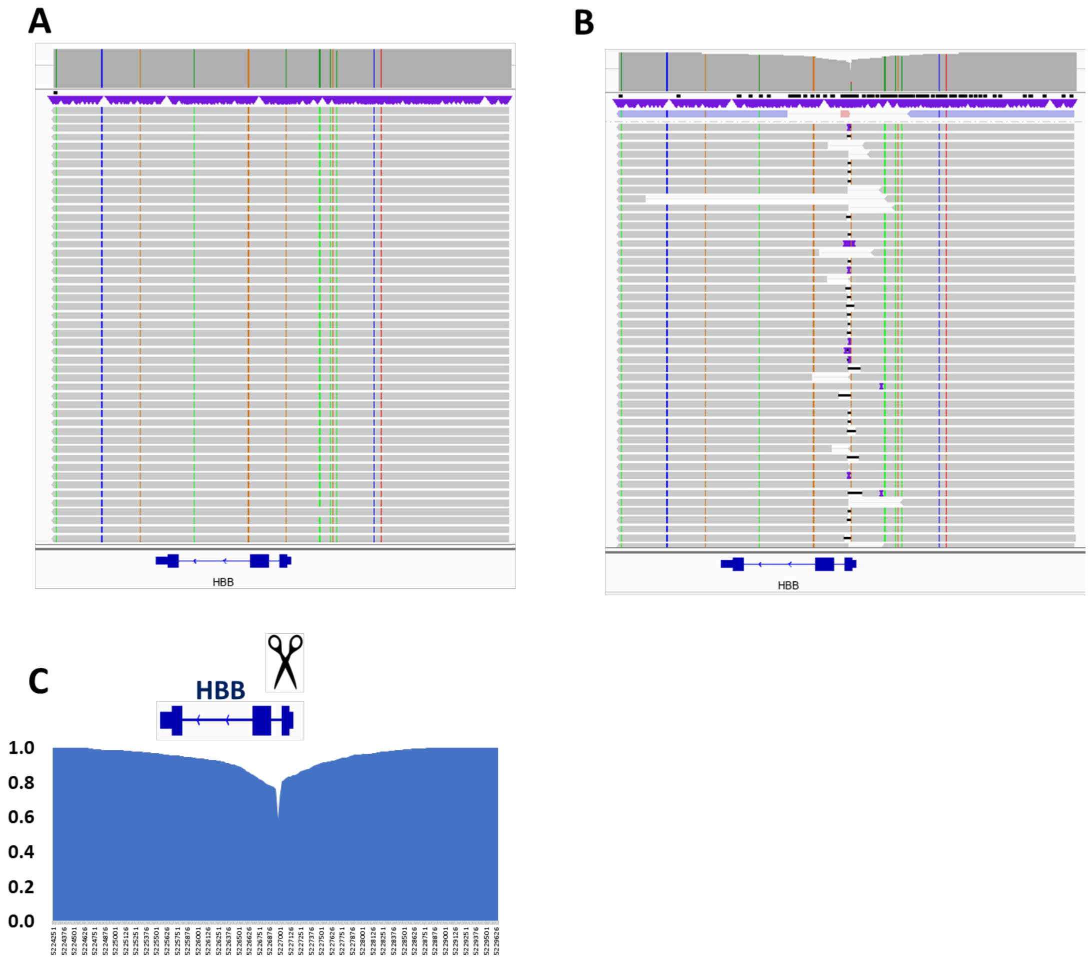

Figure S12. SMRT-seq UMI consensus read alignment. IGV alignment of UMI consensus reads to *HBB* for (**A**) untreated, and (**B**) R-66S RNP treated SCD HSPCs from Donor#1 showing read coverage depletion pattern around the R-66S cut site only in RNP sample from reads containing small INDELs and LDs. Vertical colored lines indicate SCD mutation, and 11 additional common SNPs found within the amplicon in the individual patient donor compared to the reference genome. (**C**) The read coverage depletion pattern of the RNP-treated SCD HSPCs, normalized by that of the untreated sample showing asymmetric pattern.

Figure S13

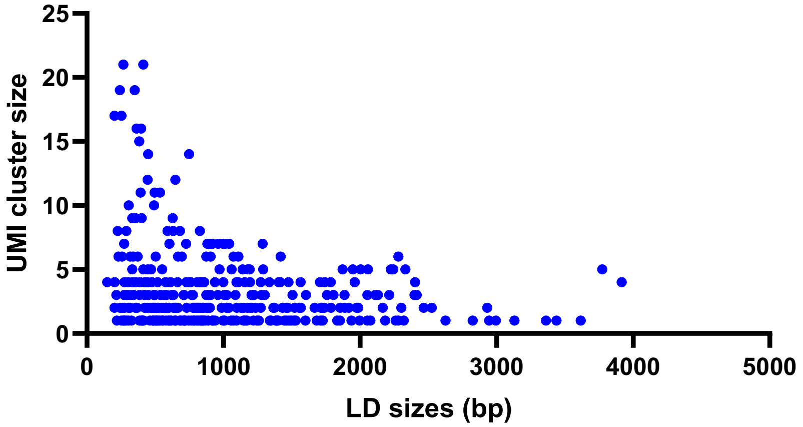

Figure S13. UMI cluster size for each LD pattern. In R-66S RNP treated SCD HSPC from Donor#1, we found 35.4% of LDs (>=200 bp). From 3473 UMI consensus sequences, we identified 1229 LD-containing sequences that form 381 unique LD patterns, demonstrating a diverse range of LDs. Of the 381 unique LD patterns, 130 were captured by one UMI consensus sequence, 90 by two UMI consensus sequences, 46 by three UMI consensus sequences, *etc*. Note that 21 UMI consensus sequences have the LD of the same size (267bp) and start position, accounting for 0.6% of the total UMI consensus sequences.

Figure S14

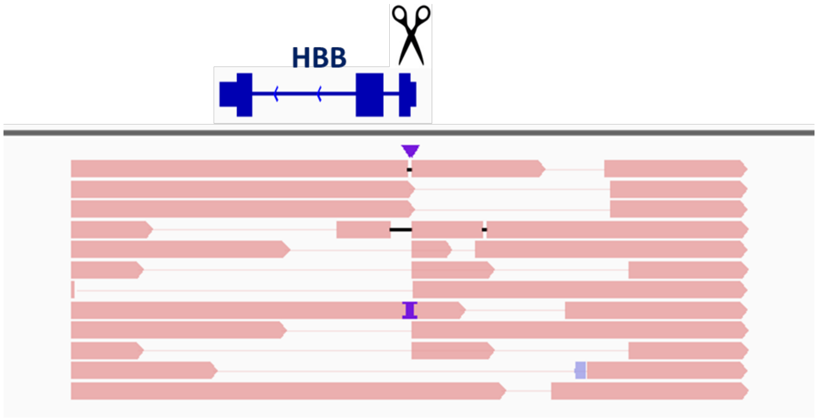

Figure S14. SMRT-seq identified different types of LD. An example of read alignment to *HBB* showing different types of LDs including asymmetric LDs spanning the cut-site, LDs away from the cut-site, and multiple LDs on the same allele. Integrative Genomics Viewer (IGV) was used for the visual exploration of genomic data.

Figure S15

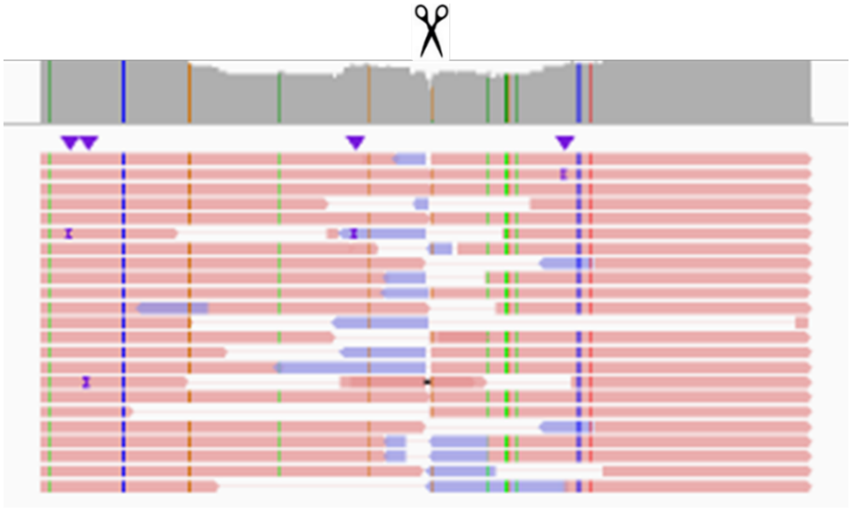

Figure S15. SMRT-seq identified different types of local complex rearrangements. IGV visualization of aligned of UMI consensus sequences containing large insertions (>= 50 bp) at the on-target cut-site. Some of the insertions were accompanied by small INDELs, intermediate deletions or LDs. Most of the inserted sequences mapped to sequences within the *HBB* near the cut-site, demonstrating local complex chromosomal rearrangements. The rest of the inserted sequences are mapped to the other chromosomal location in the human genome (hg19).

Figure S16

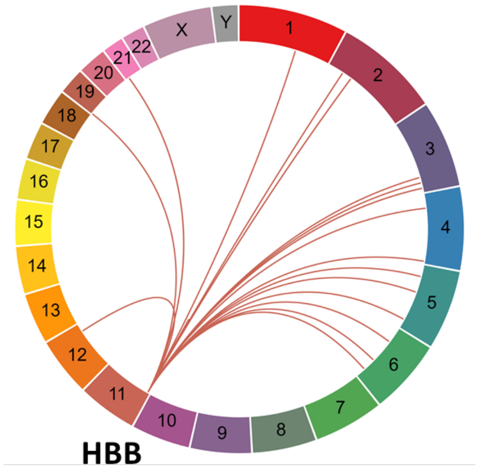

Figure S16. Circos plot showing insertion donating site on Hg19 in R-66S RNP treated SCD HSPCs. About 66% of the inserted sequences are homologous to those at or close to the *HBB* cut-site, demonstrating local complex chromosomal rearrangements within the β-globin locus. The rest of the inserted sequences are mapped to the other chromosomal locations in the human genome without known association with the sequence at the on-target cut-site.

Figure S17

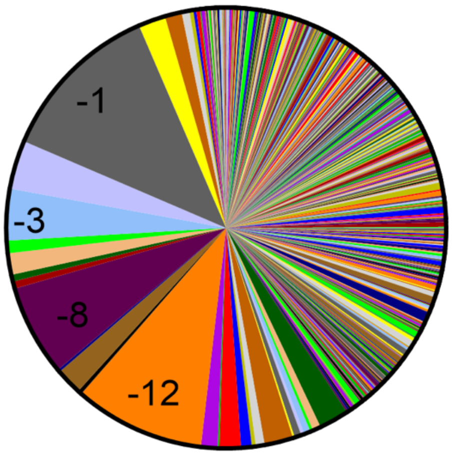

Figure S17. Pieplot showing broad spectrum of gene-editing outcomes. Of the 3478 UMI consensus sequences, a total of 536 unique mutations were identified, including 67 small INDELs, 44 intermediate deletions, 381 LDs, and 44 large insertions. The SMRT-seq identified allelic diversity, including large modifications (536 unique gene modification patterns), is >8-fold higher than characterized based on small INDELs (67 small INDEL patterns).

Figure S18

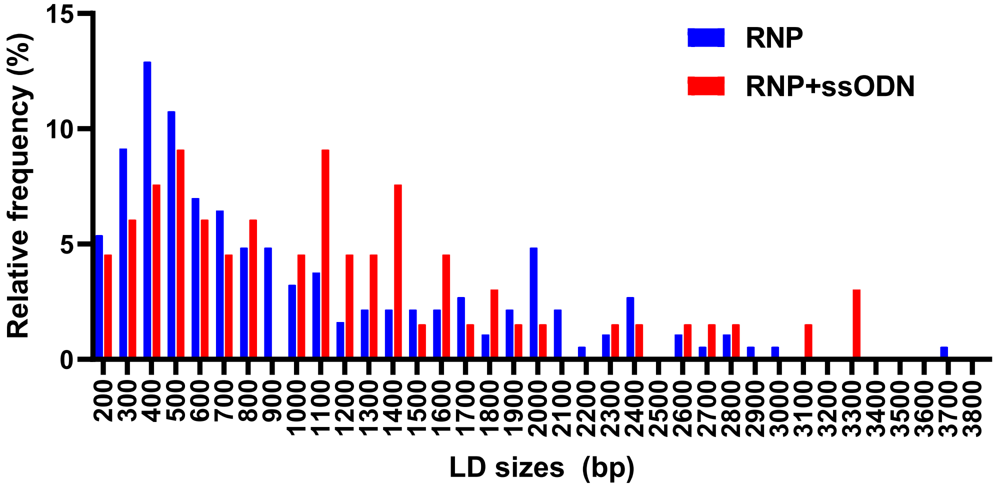

Figure S18. Relative LD frequency in RNP compared to RNP and ssODN treated SCD HSPCs. In the presence of ssODN, the size of LD distribution is skewed, with lower rates for shorter LDs and higher rates for longer LDs.

Figure S19

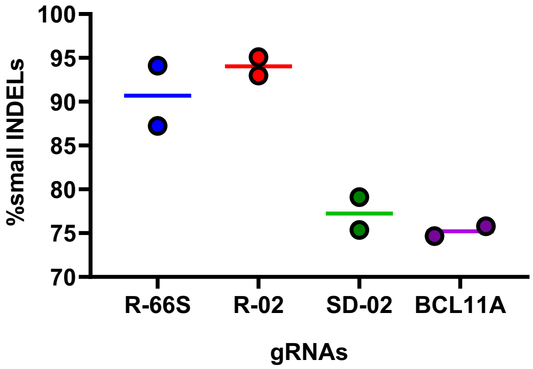

Figure S19. High level of on-target small INDELs measured by S-R NGS. R-66S, R-02, SD-02 and BCL11A gRNAs were complexed with HiFi *Sp*Cas9 and delivered as RNP to SCD HSPCs from Donor #1. All gRNAs showed high on-target small INDEL rates measured by S-R NGS, similar to that previously reported.

Figure S20

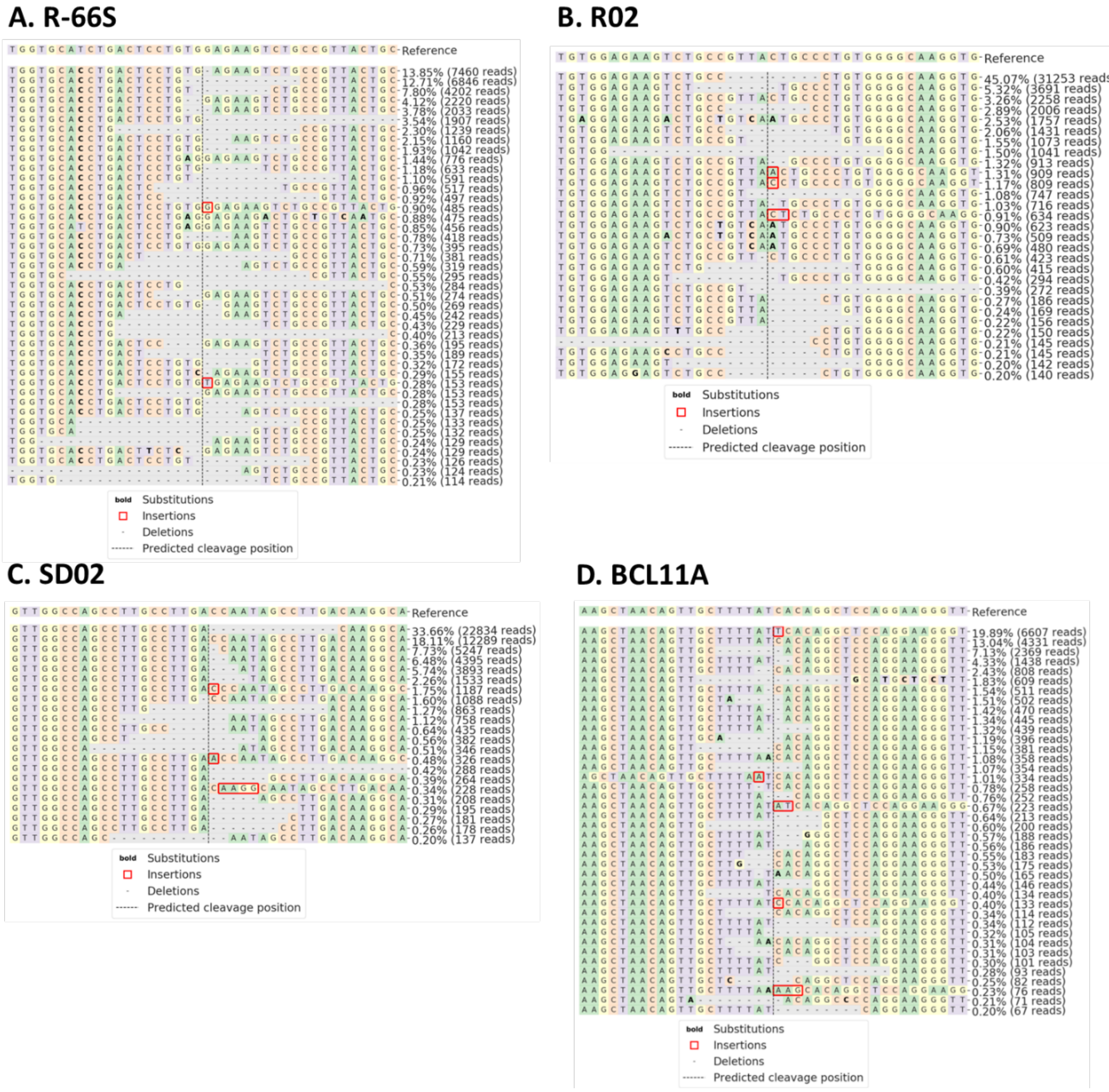

Figure S20. Distribution of the most frequent allele modifications identified with CRISPResso2 around cleavage sites in SCD HSPCs. (**A**) R-66S, (**B**) R-02, (**C**) SD-02 and (**D**) BCL11A gRNAs were complexed with HiFi *Sp*Cas9 and delivered as RNP to SCD HSPCs from Donor #1. All distributions showed high rates of on-target small INDELs profile by S-R NGS as previously reported.

Figure S21

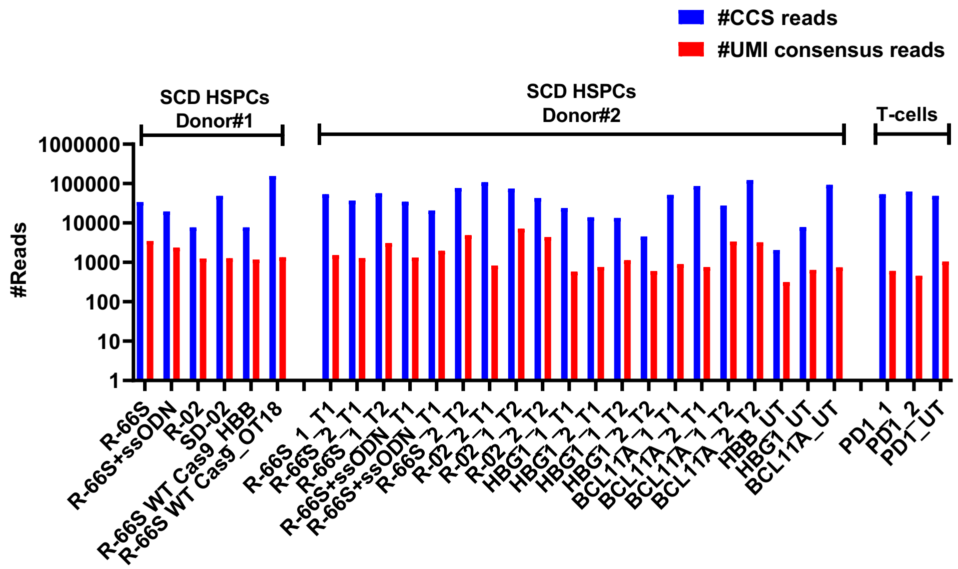

Figure S21. SMRT-seq with UMI read number after UMI processing. Number of Q20 CCS reads and UMI consensus sequences aligned to the reference for each sample. Samples from SCD HSPCs Donor#1 edited with R-66S±ssODN and R-02 at *HBB*, SD-02 at *HBG1* and R-66S gRNA complexed with WT Cas9 (R-66S WT Cas9) at *HBB* and *OT18*. SCD HSPCs Donor#2 edited with R-66S±ssODN, R-02, SD-02, BCL11A and analyzed on day-4 post-delivery (T1), and after 14 days erythroid differentiation (on day-17 post-delivery) (T2). T-cells edited with PD-1.

Figure S22

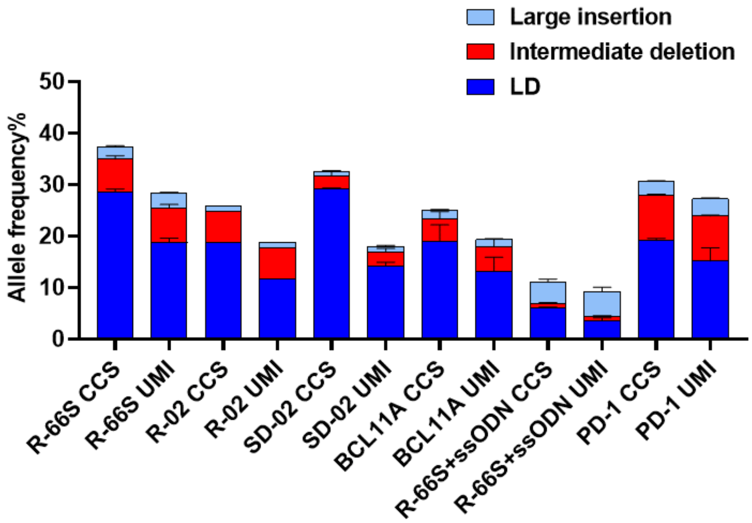

Figure S22. Mitigation of PCR bias in UMI consensus reads. We compared frequencies of LD, intermediate deletion, and large insertion quantified using UMI consensus reads compared to CCS reads and found that correction of PCR bias consistently led to decreased rates of LDs in SCD HSPCs from Donor#2 edited with R-66S, R-66S+ssODN, R-02, SD-02, BCL11A, and PD-1.

Figure S23

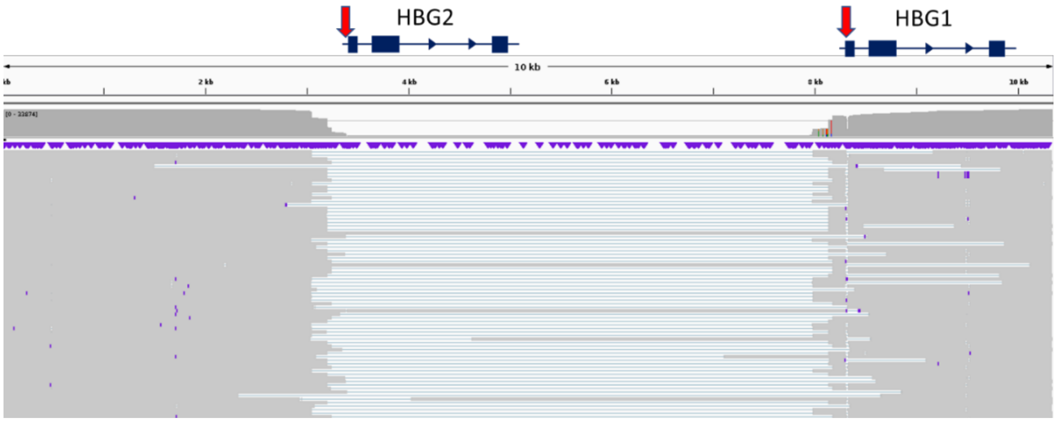

Figure S23. Schematics of *HBG* locus. Previously, considerable LD levels upon simultaneous cleavage have been reported removing the entire *HBG2* gene and part of the *HBG1* promoter creating *HBG-HBG2* fusion allele. *HBG1*-specific L-R PCR with 6.4 kb amplicon size could be misleading in the presence of another on-target cut site on *HBG2*, 4.9 kb upstream of the cut site on *HBG1*. To understand the types of large intergenic modification missed by *HBG1*-specific sequencing, we amplified and sequenced the 10 kbp region, including *HBG1* and *HBG2*. We observed intergenic LD extending further upstream of the cut site on *HBG2* and/or downstream of the cut site on *HBG1*, removing both *HBG1* and *HBG2*.

Figure S24

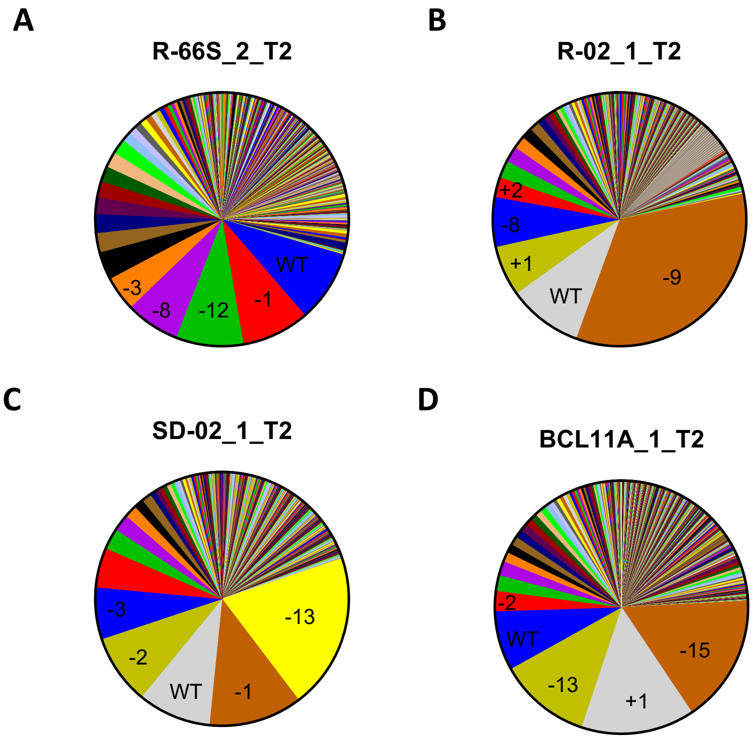

Figure S24. Pieplot showing broad spectrum of gene-editing outcomes in SCD HSPCs from Donor #2. The SMRT-seq results revealed a broad spectrum of unintended large modifications at or near the Cas9 cut-site in R-66S, R-02, SD-02 and BCL11A RNP treated SCD HSPCs. (**A**) In the R-66S_2_T2 sample, of the 4897 UMI consensus sequences, a total of 843 unique gene modification patterns were identified, including 86 small INDEL, 65 intermediate deletions, 567 LDs, and 125 large insertions. The SMRT-seq identified allelic diversity including large modifications (843 unique gene modification patterns) is >9.8-fold higher than characterized based on small INDELs (86 small INDEL patterns). (**B**) In the R-02_1_T2 sample, of the 7154 UMI consensus sequences, a total of 718 unique gene modification patterns were identified, including 83 small INDEL, 74 intermediate deletions, 495 LDs, and 86 large insertions. The SMRT-seq identified allelic diversity including large modifications (718 unique gene modification patterns) is >8.7-fold higher than characterized based on small INDELs (83 small INDEL patterns). (**C**) In the SD-02_1_T2 sample, of the 1130 UMI consensus sequences, a total of 236 unique gene modification patterns were identified, including 54 small INDEL, 18 intermediate deletions, 158 LDs, and 7 large insertions. The SMRT-seq identified allelic diversity including large modifications (236 unique gene modification patterns) is >4.4-fold higher than characterized based on small INDELs (54 small INDEL patterns). (**D**) In the BCL11A_1_T2 sample, of the 3375 UMI consensus sequences, a total of 528 unique gene modification patterns were identified, including 80 small INDEL, 49 intermediate deletions, 364 LDs, and 49 large insertions. The SMRT-seq identified allelic diversity including large modifications (528 unique gene modification patterns) is >6.6-fold higher than characterized based on small INDELs (80 small INDEL patterns).

Figure S25

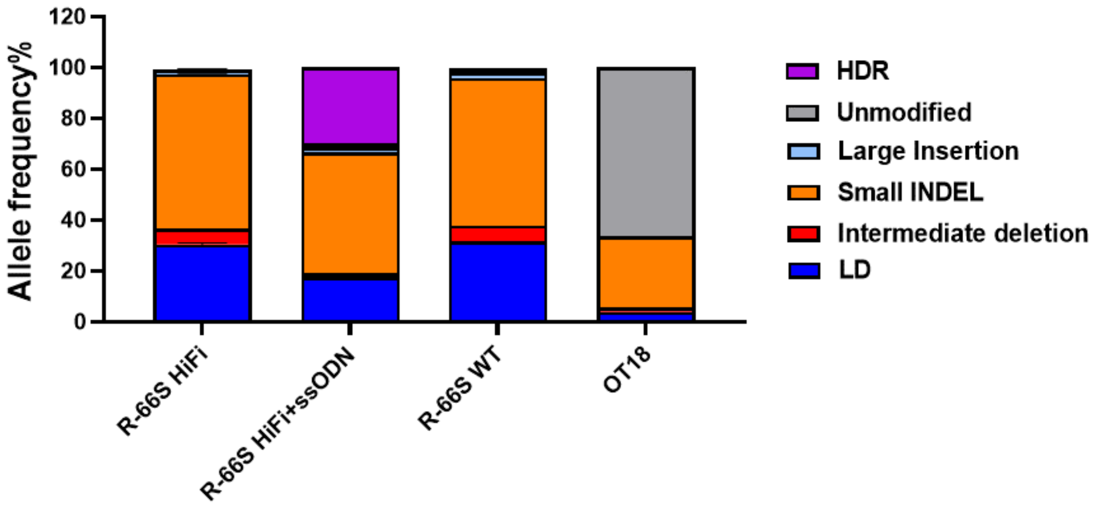

Figure S25. Gene modifications induced by HiFi Cas9 and WT Cas9. We delivered R-66S gRNA complexed with HiFi Cas9 and WT Cas9, respectively, into SCD HSPCs from Donor#1 and compared gene modification rates at the HBB on-target site as well as the known off-target site OT18. HiFi Cas9 and WT Cas9 treated samples showed similar LD rates (30.3% vs. 31.5%) and intermediate deletion rates (6.2 vs. 6.3%) quantified by SMRT-seq with UMI. WT Cas9 showed a higher rate of large insertion than HiFi Cas9 (2.2% vs. 1.6%). The LD rate at the OT18 in the WT Cas9 treated sample was 3.9%.

Figure S26

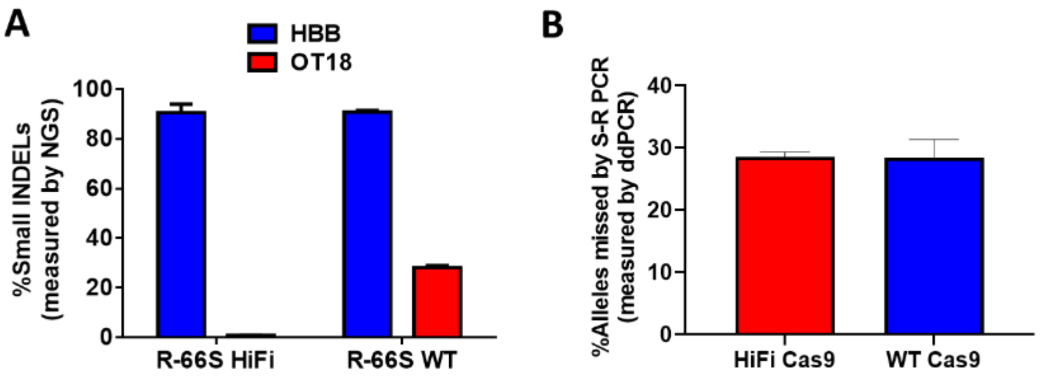

Figure S26. Small INDELs and LDs induced by HiFi Cas9 and WT Cas9. We delivered R-66S gRNA complexed with HiFi Cas9 or WT Cas9 and quantified the small INDELs by S-R NGS and LD by ddPCR. (**A**) The HiFi Cas9 gave similar small INDELs rates compared with WT Cas9 at *HBB*, but significantly reduced the small INDELs rates at the most active off-target site (OT18). (**B**) HiFi Cas9 and WT Cas9 treated samples showed similar LD frequencies at *HBB* measured by ddPCR allelic drop-off assay similar to reported by SMRT-seq with UMI in **Figure S26**.

Figure S27

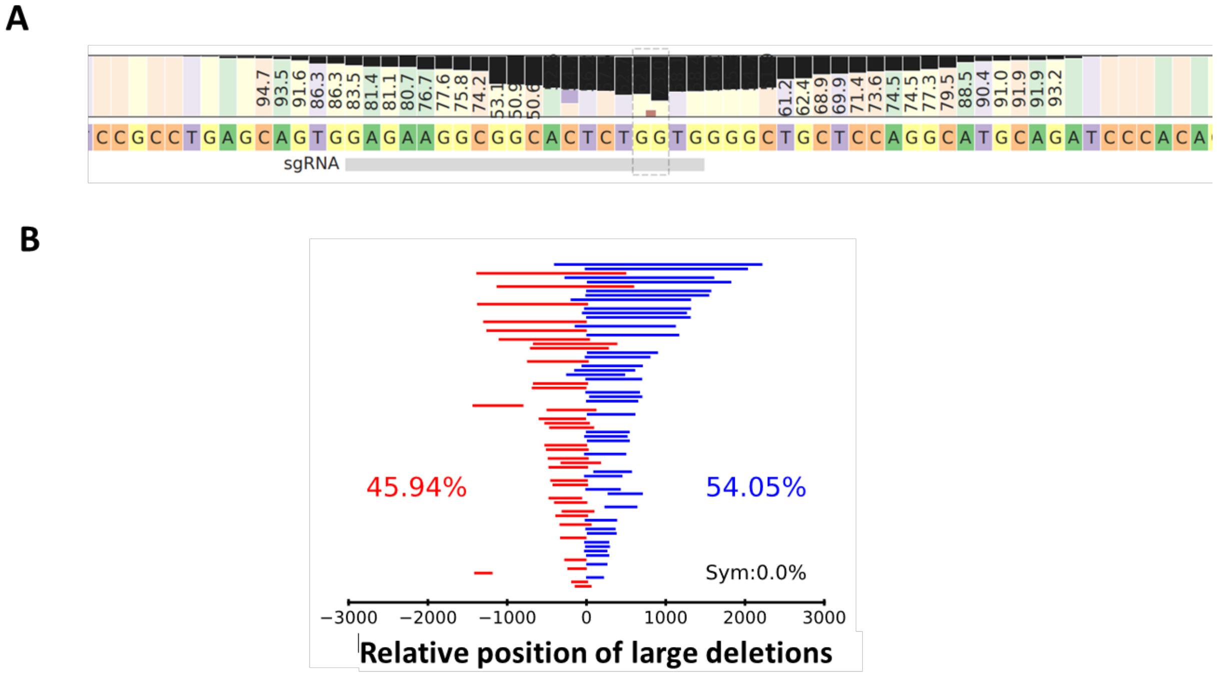

Figure S27. Large deletions in PD-1 targeted primary T-cells. We performed SMRT-seq with UMI for the gRNA targeting a PD-1 locus in T cells (**A**) Nucleotide distribution around the PD-1 gRNA generated by CRISPResso2 (30). At each base in the reference amplicon, the percentage of each base as observed in sequencing reads is shown (A = green; C = orange; G = yellow; T =

purple). Black bars show the percentage of reads for which that base was deleted. (**B**) LD patterns were mapped relative to the Cas9 cut-site to show deletion size and location.

Figure S28

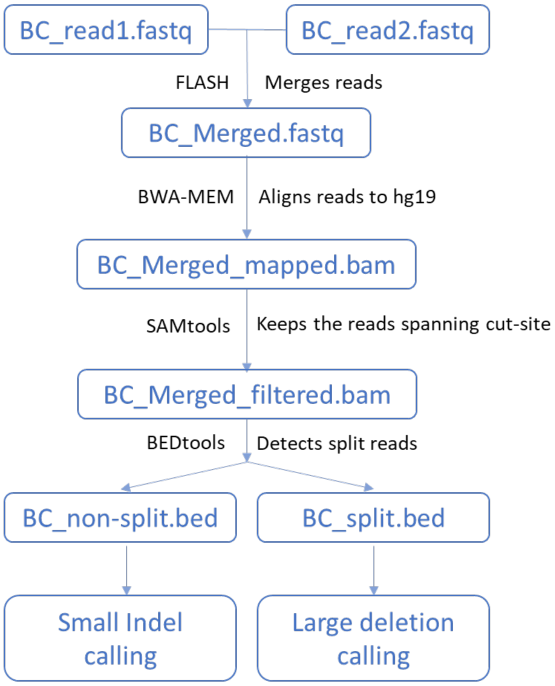

Figure S28. LongAmp-seq bioinformatics pipeline for calling small INDELs and LDs. The raw sequencing data from Illumina MiSeq were demultiplexed by bcl2fastq from Illumina and merged using FLASH (52). Merged reads were aligned to reference genome hg19 using BWA-MEM (53), and the reads that were not spanning the cut site were filtered out with SAMtools (55). The split reads were identified using BEDtools (56) and further processed to break-point based variant calling, while the small INDEL patterns were generated by CRISPResso2 (30) using the unsplit reads.

Figure S29

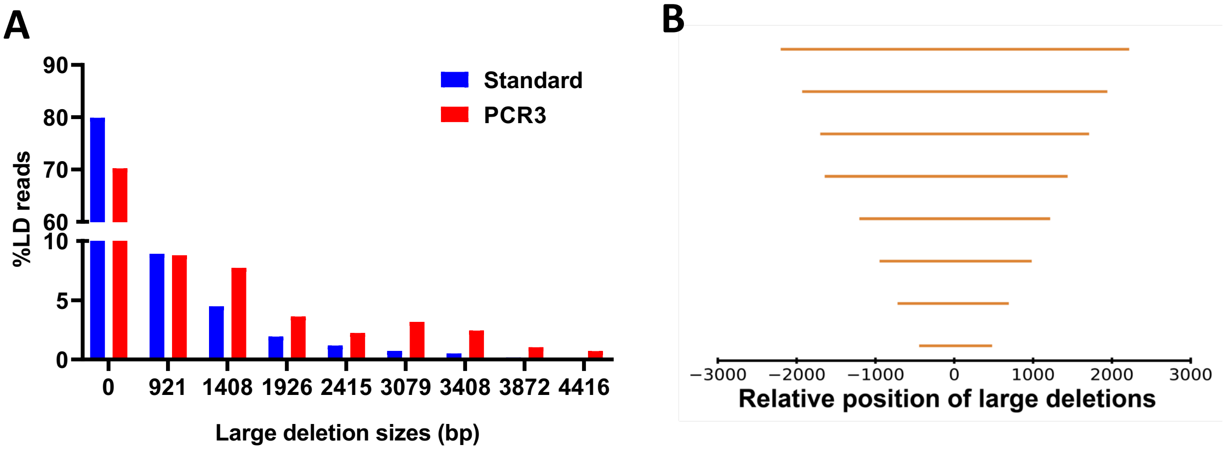

Figure S29. Benchmark the LongAmp-seq using known mixtures of allelic variants. The same DNA template standard with pre-determined allele frequency and PCR3 product analyzed by SMRT-seq in Supplementary Figure S11 were processed by LongAmp-seq. (**A**) LongAmp-seq measured 70% Template 9 (corresponding to unmodified HBB) while the frequency of Template 9 in the original DNA standard used for the library prep was 79.9%, showing underestimate of the unmodified allele and overestimate of the LD-containing alleles by LongAmp-seq, largely due to having more PCR duplicates of Templates 1-8 compared to that of Template 9. (**B**) LongAmp-seq identified LDs (≥200 bp) were clustered based on their sizes and locations to identify unique LDs. Instead of UMI cluster size-based filtering used in SMRT-seq, LD patterns with below 0.01% read number of total aligned reads were considered background noise and filtered out, which removed the false-positive LDs. Each unique LD was mapped relative to the Cas9 on-target cut-site. LongAmp-seq was able to identify the correct alleles (Templates 1-9) presented in the original DNA standard.

Figure S30

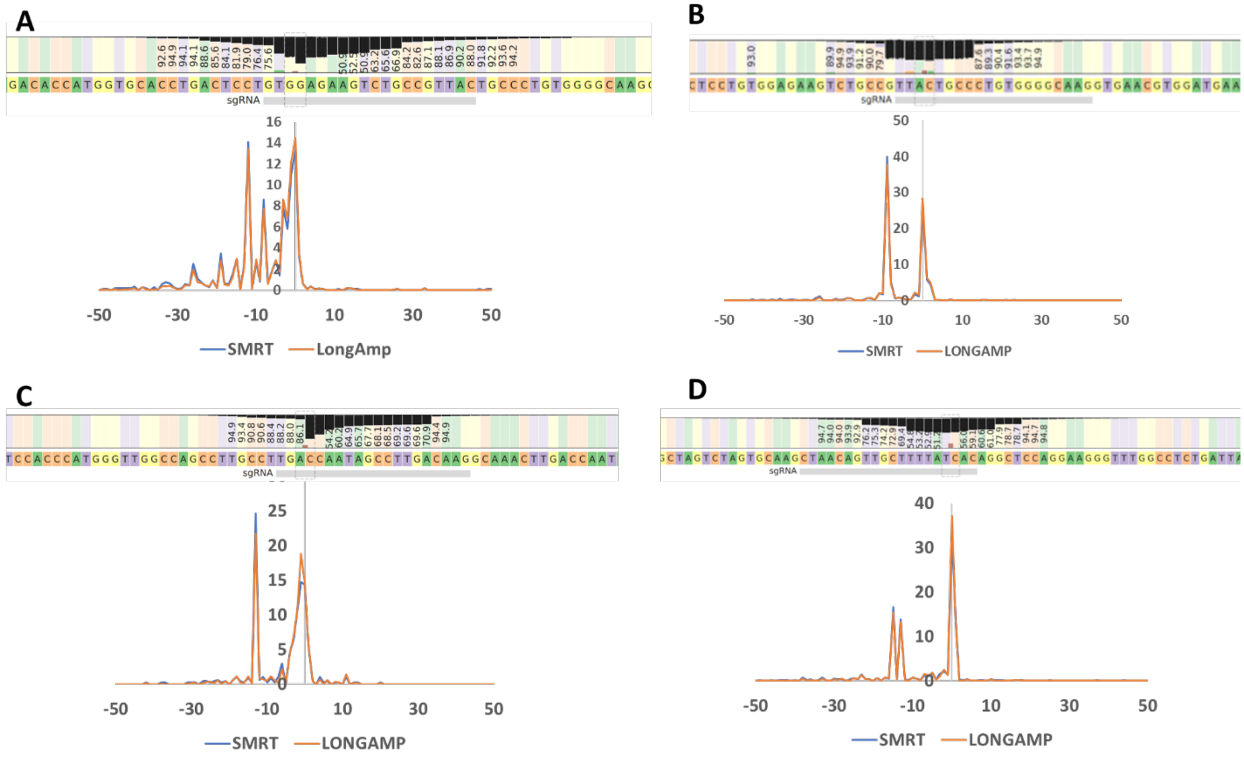

Figure S30. Small INDEL profiles by SMRT-seq and LongAmp-seq. The small INDELs at the (A) R-66S, (B) R-02, (C) SD-02 and (D) BCL11A RNP treated SCD HSPCs from Donor #2 were detected by SMRT-seq and LongAmp-seq assays respectively. SMRT-seq and LongAmp-seq reads without large gene modifications were processed by CRISPResso2 and showed overlapping small INDEL signatures and excellent correlation of the small INDEL rates.

Figure S31

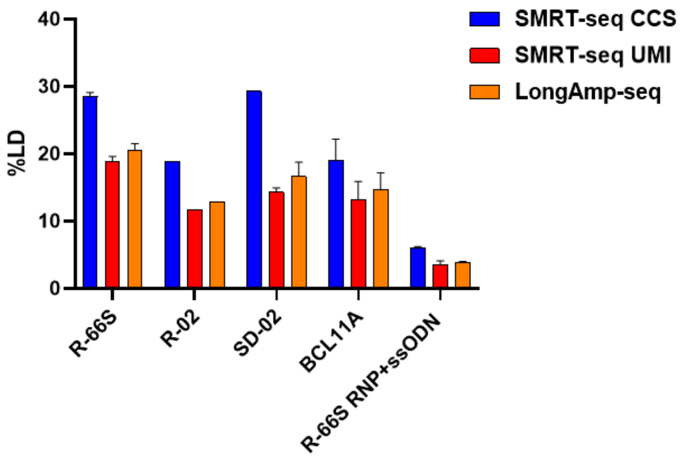

Figure S31. LongAmp-seq and SMRT-seq quantified LDs in SCD HSPCs. We sequenced the same set of samples by SMRT-seq and LongAmp-seq and compared SMRT-seq raw CCS (SMRT CCS), SMRT-seq with UMI filtered and consolidated (SMRT UMI) and LongAmp-seq. LD rates were consistently highest by SMRT-seq without filtering for UMI, followed by LongAmp-seq and SMRT-seq with UMI. The percentage of LDs obtained using LongAmp-seq (quantified as the number of reads containing unique LDs divided by the total reads) was compared to the LD allele frequency quantified by SMRT-seq using UMI consensus reads and showed excellent correlation, although without UMI-based correction of PCR bias and error, LongAmp-seq gave slightly higher LD rates.

Figure S32

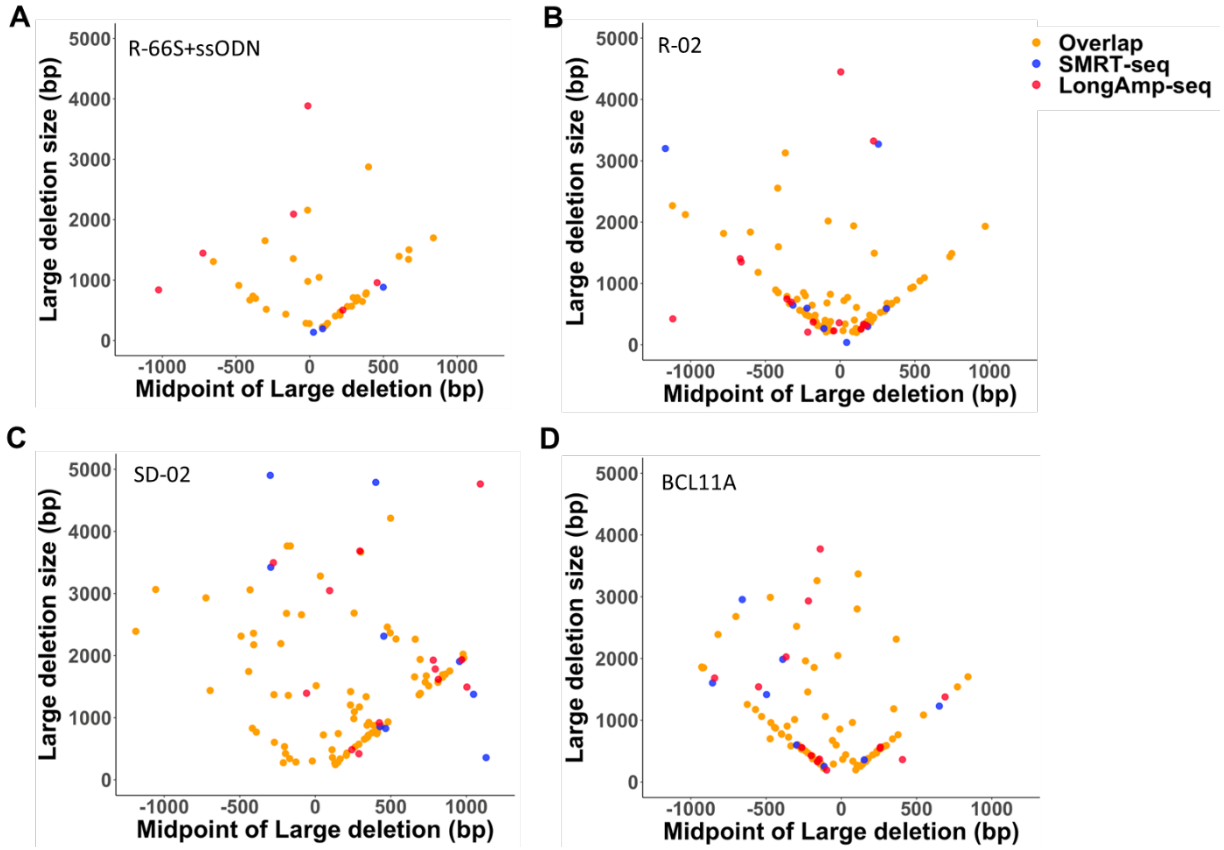

Figure S32. Overlap between LongAmp-seq and SMRT-seq identified LD patterns in SCD HSPCs. The LD patterns identified by SMRT-seq and LongAmp-seq were plotted based on the location of the midpoint of LD (x-axis) and LD size (y-axis). There is a high level of overlap between LongAmp-seq identified LDs and that identified by SMRT-seq. (**A**) R-66S RNP with ssODN, (**B**) R-02 RNP, (**C**) SD-02 RNP and (**D**) BCL11A RNP treated SCD HSPCs from Donor#2.

Figure S33

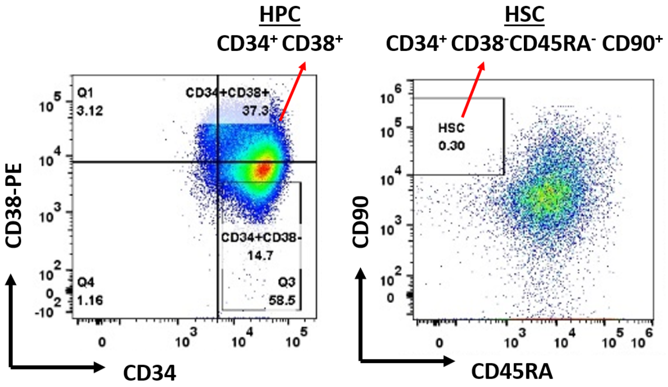

Figure S33. SCD HSPC immunophenotyping and sorting. After R-66S RNP, R-66S RNP and ssODN or mock electroporation, treated SCD HSPCs were recovered in the expansion media for 2 hours before the staining using fluorescently labeled antibodies (CD34, CD38, CD45RA, CD90) for HSPC subset analysis and FACS. The percentage of hematopoietic stem cells (HSCs, (CD34+CD38-CD45RA-CD90+) was 0.3% and the percentage of hematopoietic progenitor cells (HPCs , CD34+CD38+) was 38.3%. Sorted cells were cultured for additional days to expand and gDNA was harvested for analysis by S-R NGS and LongAmp-seq.

Figure S34

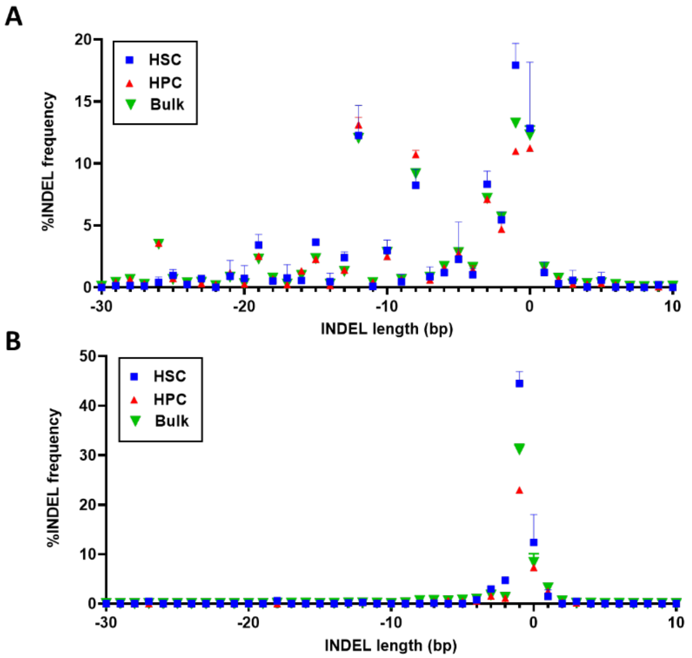

Figure S34. Small INDEL profile in HSC, HPC and bulk HSPCs. (A) R-66S RNP edited and sorted cells were analyzed by S-R NGS. (B) R-66S RNP and ssODN treated and sorted cells were analyzed by S-R NGS. We observed enrichment -1bp deletion produced by NHEJ accompanied by depletion MMEJ-repaired INDELs (notably, -26bp deletion with CCTGTG 5bp microhomologies) in HSCs.

Figure S35

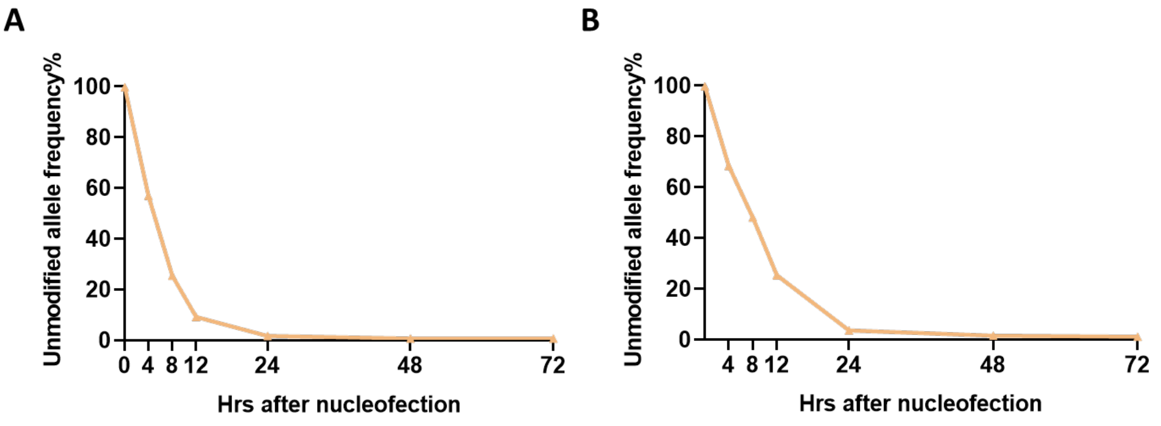

Figure S35. Frequency of unmodified alleles in edited K562 at different time points. (A) R-66WT RNP was electroporated into K562, harvested gDNAs at different time points over 3 days post-delivery, and analyzed the rates of LDs, NHEJ-led small INDELs (**Figure 5D**), and unmodified alleles by LongAmp-seq. (B) R-66WT RNP and ssODN were electroporated into K562, harvested gDNAs at different time points over 3 days post-delivery, and analyzed the rates of LDs, NHEJ-led small INDELs, HDR-mediated ssODN (**Figure 5E**), and unmodified alleles by LongAmp-seq.

Figure S36

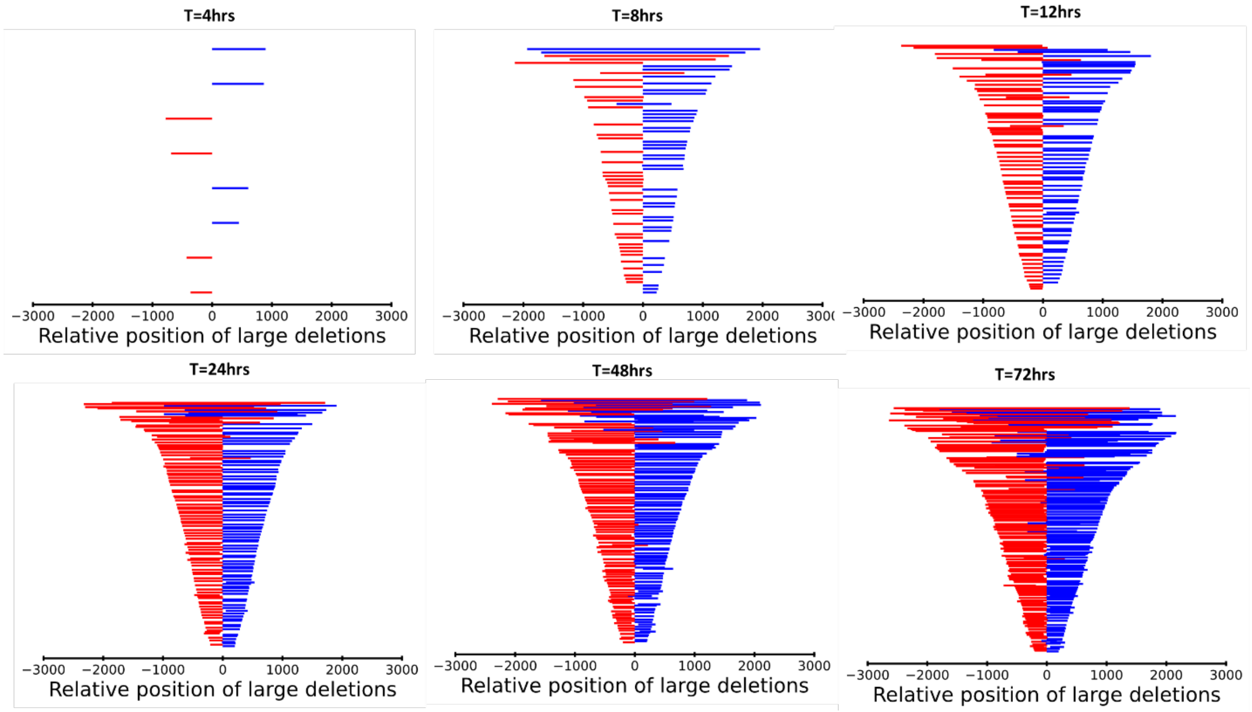

Figure S36. Sizes and distributions of LDs in K562 cells at different time points. The R-66WT RNP with sickle ssODN were delivered into the K562 erythroleukemia cell line and harvested gDNAs at different time points over 3 days post-delivery, and analyzed by LongAmp-seq. We compared the size distribution of LDs over time and found that the repair of longer LDs was slower than shorter LDs.

Figure S37

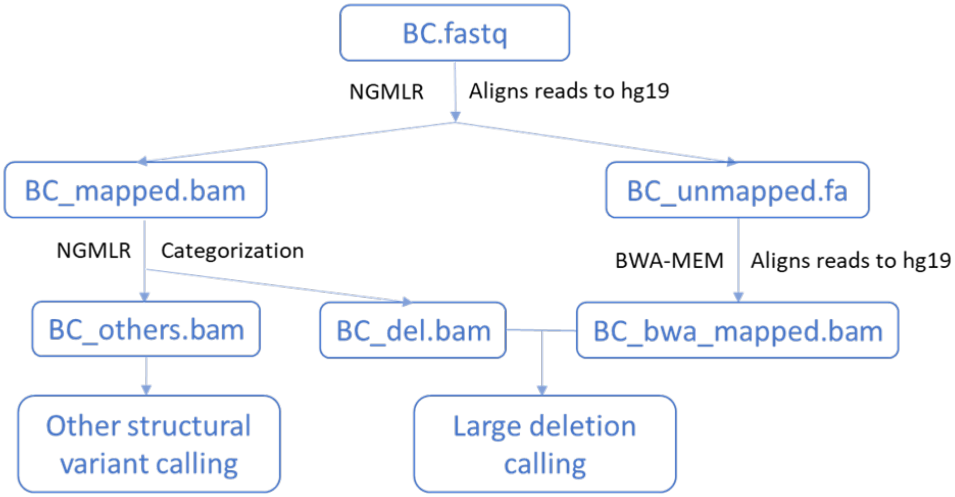

Figure S37. Nanopore long-read sequencing bioinformatics pipeline for detecting structural variants. FASTQ sequence files were processed into FASTQ using Guppy Basecaller. We first used NGMLR (57) to map all long reads reference human genome hg19., and the reads that mapped to the *HBB* region were analyzed for deletions and insertions calling. The reads that could not be mapped by NGMLR were further aligned by BWA-MEM (53) and filtered by SAMtools to include the chimeric reads carrying potential large deletions. The insertion profile was from NGMLR calling, and the large deletion profile included both NGMLR called reads, and BWA-MEM identified reads.

Figure S38

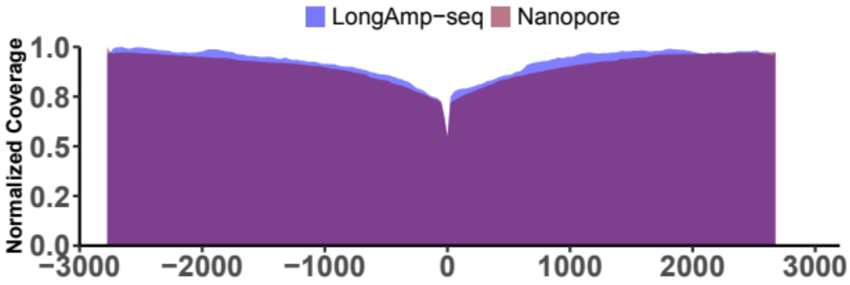

Figure S38. Comparable read coverage depletion pattern of the RNP treated sample by LongAmp-Seq and Nanopore long-reads sequencing. We used gDNA from 10,000 unique input alleles for L-R PCR to enrich the 5.5kb amplicons surrounding the *HBB* cut-site from R-66S HiFi RNP treated or untreated samples. L-R PCR products were processed by LongAmp-seq and Nanopore long-read sequencer in parallel with the total aligned read number for an average coverage over 70x of each allele. We observed a comparable read coverage depletion pattern of the RNP treated sample in both short- and long-reads sequencing, demonstrating that LongAmp-seq library preparation barely introduced extra bias.

Figure S39

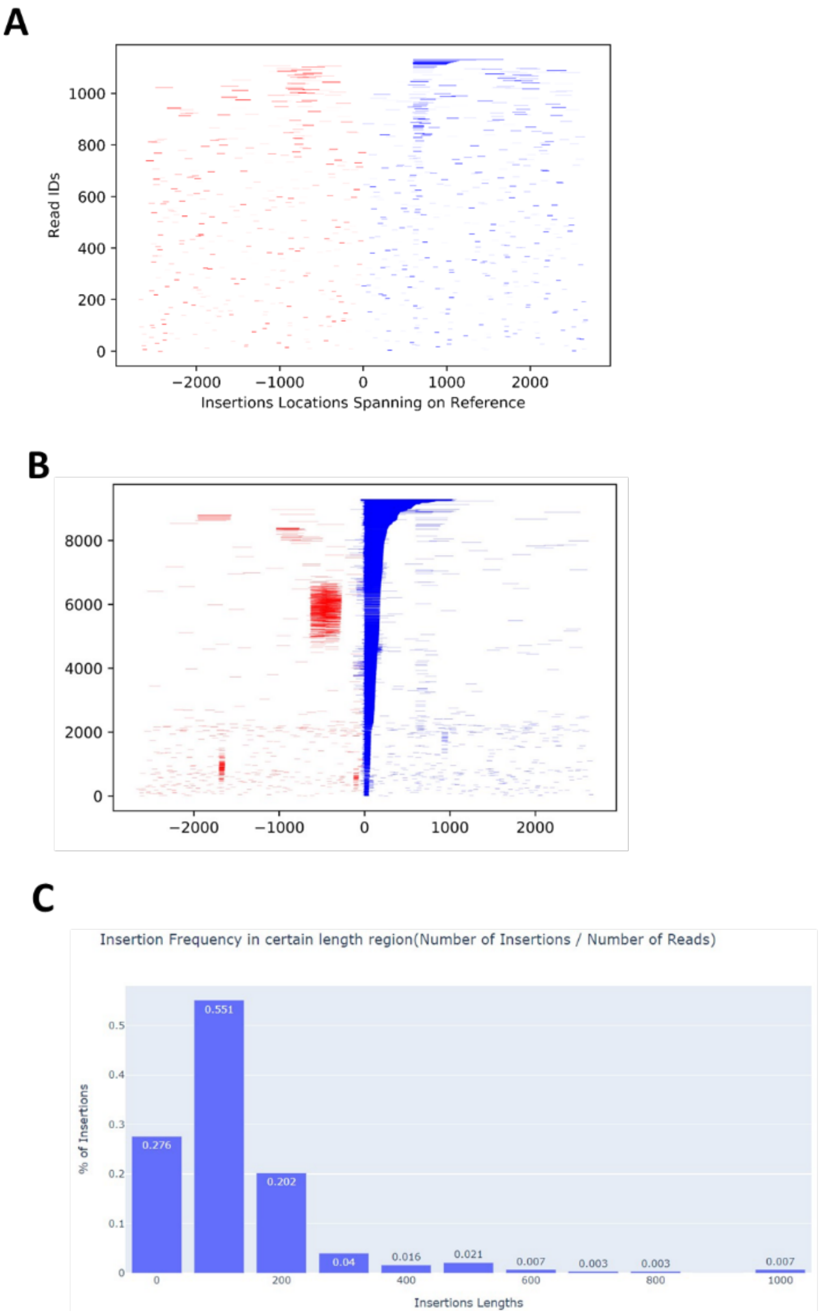

Figure S39. Large insertion and complex repair outcomes detected by Nanopore long-read sequencing. (**A**) Large insertion distributions in Untreated reads. The overall number of insertions we detected in untreated reads is 1133, from 893,629 reads in total. (**B**) Large insertion distributions in R-66S HiFi RNP treated reads. The overall number of insertions we detected in R-66 HiFi RNP treated reads is 9295, from 715,997 reads in total. (**C**) Insertion frequency among all Nanopore reads, the x-axis is the length of the insertion while the y-axis is the frequency that the insertions happen.

Supplementary Table S2. Colony genotype in S-HUDEP2 cells

	S-R NGS				PCR gel shift		ddPCR copy number			
CLONE#	NGS read#	allele1	allele2	NGS results	S-R PCR (337bp)	L-R PCR (5.5kb)	HBB copy#	REF copy#	%HBB	Correct genotype based on 3-assays
C2	1004	-12	-26	Hetero SI	expected	expected				Hetero SI
C3	923	-10	-3	Hetero SI	expected	expected				Hetero SI
C7	811	-15	-8	Hetero SI	expected	expected				Hetero SI
C8	595	-12	-1	Hetero SI	expected	expected				Hetero SI
C9	1258	-1	-9	Hetero SI	expected	expected				Hetero SI
C11	1122	-34	-3	Hetero SI	expected	expected				Hetero SI
C12	163	-2	-1	Hetero SI	expected	expected				Hetero SI
C16	879	-12	1	Hetero SI	expected	expected				Hetero SI
C20	732	-8	-4	Hetero SI	expected	expected				Hetero SI
C21	779	-21	-6	Hetero SI	expected	expected				Hetero SI
C22	780	-21	-5	Hetero SI	expected	expected				Hetero SI
C25	943	1	-12	Hetero SI	expected	expected				Hetero SI
C26	1467	-19	-12	Hetero SI	expected	expected				Hetero SI
C29	1137	-8	-6	Hetero SI	expected	expected				Hetero SI
C31	857	-18	-1	Hetero SI	expected	expected				Hetero SI
C35	877	-26	-19	Hetero SI	expected	expected				Hetero SI
C39	751	-2	-1	Hetero SI	expected	expected				Hetero SI
C40	627	-21	-8	Hetero SI	expected	expected				Hetero SI
C42	806	-10	-1	Hetero SI	expected	expected				Hetero SI
C43	1135	-36	-12	Hetero SI	expected	expected				Hetero SI
C44	2024	1	109	Hetero SI	expected	expected				Hetero SI
C46	620	-8	-1	Hetero SI	expected	expected				Hetero SI
C47	1012	-43	-15	Hetero SI	expected	expected				Hetero SI
C48	817	-2	-1	Hetero SI	expected	expected				Hetero SI
C49	663	-8	2	Hetero SI	expected	expected				Hetero SI
C50	578	-12	-1	Hetero SI	expected	expected				Hetero SI
C51	629	-3	-12	Hetero SI	expected	expected				Hetero SI
C54	67	-7	-5	Hetero SI	expected	expected				Hetero SI
C57	659	-1	-5	Hetero SI	expected	expected				Hetero SI
C58	530	-2	-1	Hetero SI	expected	expected				Hetero SI
C61	1041	-1	-26	Hetero SI	expected	expected				Hetero SI
C62	937	-6	-15	Hetero SI	expected	expected				Hetero SI
C70	984	-19	-8	Hetero SI	expected	expected				Hetero SI
C71	994	-8	-3	Hetero SI	expected	expected				Hetero SI
C73	847	-3	-1	Hetero SI	expected	expected				Hetero SI
C74	1019	-10	-19	Hetero SI	expected	expected				Hetero SI
C75	1161	-8	-18	Hetero SI	expected	expected				Hetero SI
C77	985	-18	-5	Hetero SI	expected	expected				Hetero SI
C78	1162	-12	-8	Hetero SI	expected	expected				Hetero SI
C92	778	-12	-4	Hetero SI	expected	expected				Hetero SI
C93	809	-26	-2	Hetero SI	expected	expected				Hetero SI
C94	951	-4	34	Hetero SI	expected	expected				Hetero SI
C96	823	-1	1	Hetero SI	expected	expected				Hetero SI
C98	797	-8	-3	Hetero SI	expected	expected				Hetero SI
C99	1279	-8	-5	Hetero SI	expected	expected				Hetero SI
C102	912	-15	-1	Hetero SI	expected	expected		NA		Hetero SI
C6				no reads	no band	1 expected/1 smaller	0	88	0	LD/LD
C17				no reads	no band	0 expected/2 smaller	0	125	0	LD/LD
C19				no reads	no band	no band	1	139	1	LD/LD
C28				no reads	1 expected/1 smaller	1 expected/1 smaller	0	139	0	LD/LD
C64				no reads	no band	0 expected/2 smaller	7	298	2	LD/LD
C65		NA		no reads	no band	0 expected/2 smaller	1	247	0	LD/LD
C53	449	HBD		Homo HBD	expected	1 expected/1 smaller	50	140	35	HBD/LD
C59	1305	HBD		Homo HBD	expected	1 expected/1 smaller	59	258	23	HBD/LD
C1	457	1		Homo SI	expected	1 expected/1 smaller	69	178	39	SI/LD
C4	1002	-1		Homo SI	expected	1 expected/1 smaller	67	188	36	SI/LD
C5	860	-1		Homo SI	expected	1 expected/1 smaller	88	194	45	SI/LD
C10	960	-1		Homo SI	expected	expected	42	139	30	SI/LD
C13	1091	-19		Homo SI	expected	1 expected/1 smaller	103	280	37	SI/LD
C14	830	-26		Homo SI	expected	expected	74	232	32	SI/LD
C15	1176	-21		Homo SI	expected	expected	38	36	107	Homo SI
C18	1000	-8		Homo SI	expected	1 expected/1 smaller	35	86	41	SI/LD
C23	811	-1		Homo SI	expected	expected	55	161	34	SI/LD
C24	344	-8		Homo SI	1 expected/1 smaller	expected				SI/LD
C27	884	-8		Homo SI	expected	1 expected/1 smaller	49	120	41	SI/LD
C30	780	-8		Homo SI	expected	expected	42	48	88	Homo SI
C32	959	-12		Homo SI	expected	1 expected/1 smaller	48	137	35	SI/LD
C33	651	-9		Homo SI	expected	1 expected/1 smaller	60	161	37	SI/LD
C34	936	-1		Homo SI	expected	expected	97	255	38	SI/LD
C36	859	-9		Homo SI	expected	1 expected/1 smaller	55	159	35	SI/LD
C37	793	-12		Homo SI	expected	1 expected/1 smaller	66	198	34	SI/LD
C38	807	-15		Homo SI	expected	expected	83	238	35	SI/LD
C41	872	-19		Homo SI	expected	expected	103	230	45	SI/LD
C45	723	-12		Homo SI	expected	expected	90	228	40	SI/LD
C52	701	-1		Homo SI	expected	expected	32	184	17	SI/LD
C55	655	-1		Homo SI	expected	expected	62	249	25	SI/LD
C60	957	-1		Homo SI	expected	1 expected/1 smaller	56	194	29	SI/LD
C63	1161	-19		Homo SI	expected	expected	86	312	28	SI/LD
C66	1286	1		Homo SI	expected	1 expected/1 smaller	60	242	25	SI/LD
C67	1287	1		Homo SI	expected	1 expected/1 smaller	40	182	22	SI/LD
C68	908	-9		Homo SI	expected	1 expected/1 smaller	58	199	29	SI/LD
C69	705	-19		Homo SI	1 expected/1 smaller	expected				SI/LD
C72	953	-12		Homo SI	expected	expected	130	227	57	SI/LD
C76	848	-2		Homo SI	expected	1 expected/1 smaller	53	191	28	SI/LD
C79	1034	-12		Homo SI	expected	1 expected/1 smaller	62	185	34	SI/LD
C80	1180	-12		Homo SI	expected	expected	86	183	47	SI/LD
C81	1009	-1		Homo SI	expected	1 expected/1 smaller	59	158	38	SI/LD
C82	1051	-12		Homo SI	expected	expected	135	271	50	SI/LD
C83	1523	-3		Homo SI	expected	expected	40	124	32	SI/LD
C84	1556	-21		Homo SI	expected	1 expected/1 smaller	70	231	30	SI/LD
C85	1022	-10		Homo SI	expected	expected	55	168	32	SI/LD
C86	871	-19		Homo SI	expected	1 expected/1 smaller	88	182	49	SI/LD
C87	1029	-46		Homo SI	expected	1 expected/1 smaller	20	38	52	SI/LD
C88	919	-1		Homo SI	expected	expected	26	29	88	Homo SI
C89	666	-5		Homo SI	expected	expected	11	23	45	SI/LD
C90	874	-2		Homo SI	expected	1 expected/1 smaller	22	54	40	SI/LD
C91	831	-1		Homo SI	expected	expected	18	55	33	SI/LD
C95	783	-1		Homo SI	expected	1 expected/1 smaller	58	107	54	SI/LD
C97	1066	-19		Homo SI	expected	1 expected/1 smaller				SI/LD
C100	2101	-8		Homo SI	expected	1 expected/1 smaller				SI/LD

Supplementary Table S3. Identification of colony genotype in SCD HSPCs with R-66S RNP delivery

Sample	Clone#	read#	%SCD(GtG)	%SI	%HDR(Gaa)	%HBD(GaG)	allele1	allele2	S-R NGS genotype	L-R PCR	HBB	RPP30	%HBB	Correct genotype
		S-R NGS									ddPCR			
Biological replicate 1	clone1	3021	0	99	1	0	-12		homo SI		257	180	43	SI/LD
	clone2	3935	0	99	0	0	-1	-3	hetero SI		788	472	67	hetero SI
	clone3	3927	0	99	1	0	-2	-8	hetero SI		707	401	76	hetero SI
	clone5	3184	0	11	2	86	HBD		HBD/HBD	SHIFT	197	141	40	HBD/LD
	clone6	2401	0	87	2	10	-6		homo SI	SHIFT	321	232	38	SI/LD
	clone7	5431	0	99	1	0	-1		homo SI	SHIFT	500	347	44	SI/LD
	clone8	5879	0	99	0	0	-12		homo SI		329	167	97	homo SI
	clone9	6186	0	100	0	0	-48		homo SI	SHIFT	441	306	44	SI/LD
	clone10	NA							no band		558	557	0	LD/LD
	clone11	4645	0	99	1	0	-4	-10	hetero SI		538	297	81	hetero SI
	clone13	5356	0	67	1	32	-1	HBD	SI/HBD		1357	794	71	SI/HBD
	clone14	2587	0	98	2	0	-1	-12	hetero SI		177	93	90	hetero SI
	clone15	5314	0	99	1	0	-1	-12	hetero SI		616	321	92	hetero SI
	clone16	4975	0	99	1	0	-12		homo SI	SHIFT	178	119	50	SI/LD
	clone17	1341	0	97	3	0	-8		homo SI		653	450	45	SI/LD
	clone18	8328	0	99	1	0	-8	-15	hetero SI		1417	717	98	hetero SI
	clone19	4185	0	99	1	0	-10		homo SI	SHIFT	1005	665	51	SI/LD
	clone20	2750	0	99	1	0	-19		homo SI	SHIFT	768	525	46	SI/LD
	clone21	3940	0	99	1	0	-12		homo SI		568	385	48	SI/LD
	clone23	6141	0	100	0	0	-5	-12	hetero SI		431	216	100	hetero SI
	clone24	6758	0	99	1	0	-1	-2	hetero SI		692	361	92	hetero SI
	clone25	5667	0	100	0	0	-1	-2	hetero SI		550	285	93	hetero SI
	clone26	NA							no band		685	677	1	LD/LD
	clone27	3009	0	99	1	0	-1		homo SI	SHIFT	345	239	44	SI/LD
	clone28	3394	0	99	1	0	-8	1	hetero SI		685	357	92	hetero SI
	clone29	4023	0	100	0	0	-15	16	hetero SI		525	282	86	hetero SI
	clone30	3643	0	99	1	0	-12	-3	hetero SI		406	201	102	hetero SI
	clone31	4930	0	99	1	0	-33		homo SI	SHIFT	465	308	51	SI/LD
	clone32	6986	0	100	0	0	-15		homo SI	SHIFT	362	235	54	SI/LD
	clone34	4231	0	100	0	0	-15	-8	hetero SI		777	404	92	hetero SI
	clone35	3998	0	99	1	0	-1		homo SI	SHIFT	559	393	42	SI/LD
	clone36	3563	0	99	1	0	-12	1	hetero SI		447	234	91	hetero SI
	clone37	6509	0	100	0	0	-12	-8	hetero SI		660	338	95	hetero SI
	clone38	3338	0	98	2	0	-26	-1	hetero SI		731	443	65	hetero SI
	clone39	4604	0	100	0	0	-12		homo SI	SHIFT	254	175	45	SI/LD
	clone40	5153	0	99	0	0	-1	-5	hetero SI		1073	590	82	hetero SI
	clone41	3838	0	99	1	0	-1	-12	hetero SI		288	155	86	hetero SI
	clone42	3831	0	99	1	0	-12	-26	hetero SI		523	270	94	hetero SI
	clone43	3449	0	99	1	0	-12	-1	hetero SI		525	265	98	hetero SI
	clone44	2682	0	29	1	70	-1	HBD	SI/HBD		623	390	60	SI/HBD
	clone46	4710	0	99	1	0	-1	-8	hetero SI		528	273	93	hetero SI
	clone47	3218	0	99	1	0	-15	10	hetero SI		417	216	93	hetero SI
	clone48	NA							no band		242	230	5	LD/LD
	clone49	4607	0	99	1	0	-1		homo SI		786	394	99	homo SI
	clone50	5620	0	99	1	0	-3		homo SI		494	326	52	SI/LD
	clone51	3236	0	99	0	0	-36	-13	hetero SI		610	298	105	hetero SI
	clone52	3691	0	99	0	0	-1		homo SI		388	197	97	homo SI
	clone53	3796	0	98	2	0	-3		homo SI		202	138	46	SI/LD
	clone54	3092	0	99	1	0	-1		homo SI	SHIFT	1043	697	50	SI/LD
	clone55	5342	0	100	0	0	-1		homo SI		798	405	97	homo SI
	clone56	6179	0	99	1	0	-51	-1	hetero SI		343	163	110	hetero SI
	clone57	3275	0	99	1	0	-12		homo SI	SHIFT	431	317	36	SI/LD
	clone58	4758	0	99	1	0	-15	-8	hetero SI		446	243	84	hetero SI
	clone59	3570	0	99	1	0	-16	-12	hetero SI		511	240	113	hetero SI
	clone61	3965	0	99	1	0	-15	-8	hetero SI		215	109	97	hetero SI
	clone62	4307	0	98	2	0	3		homo SI	SHIFT	476	324	47	SI/LD
	clone63	5212	2	98	1	0	-8		homo SI	SHIFT	156	105	49	SI/LD
	clone67	3649	0	99	1	0	-19	-16	hetero SI		211	108	95	hetero SI
	clone68	6227	0	50	1	48	-1	HBD	SI/HBD		351	183	92	SI/HBD
	clone70	NA							no band		328	320	3	LD/LD
	clone71	3783	0	98	2	0	-15	-1	hetero SI		65.8	30	119	hetero SI
	clone72	4894	0	98	2	0	-28	-16	hetero SI		104	45.6	128	hetero SI
	clone73	2950	0	98	1	0	-12	-5	hetero SI		83.8	44	90	hetero SI
	clone74	3440	0	98	2	0	-12		homo SI	SHIFT	117	73.4	59	SI/LD
	clone75	5387	0	99	1	0	-7		homo SI	SHIFT	918	636	44	SI/LD
	clone76								no band	SHIFT	180	181	-1	LD/LD
	clone77	5399	0	99	1	0	-12	-3	hetero SI		281	135	108	hetero SI
	clone80	7112	0	99	1	0	-1		homo SI		157	76.4	105	homo SI
	clone81	3326	1	5	2	92	HBD		HBD/HBD		119	79.8	49	HBD/LD
	clone82	NA							no band		24.4	23.4	4	LD/LD
	clone83	5198	0	99	1	0	-45	-16	hetero SI		160	80.1	100	hetero SI
	clone84	6018	0	100	0	0	-38	-26	hetero SI		520	262	98	hetero SI
	clone85	1515	0	99	1	0	-1		homo SI		154	104	48	SI/LD
	clone87	5667	1	98	1	0	-28		hetero SI		62	30.9	101	hetero SI
	clone88	8280	0	99	1	0	-26	-28	hetero SI		149	67.8	120	hetero SI
	clone90	3774	0	58	1	41	-3	HBD	SI/HBD		324	160	103	SI/HBD
	clone91	3900	0	99	1	0	-15	-1	hetero SI		150	76.4	96	hetero SI
	clone92	5093	0	99	1	0	-10	2	hetero SI		518	275	88	hetero SI
	clone93	3652	0	99	1	0	-15	-8	hetero SI		178	94	89	hetero SI
	clone95	2156	2	92	6	0	-12		homo SI	SHIFT	47.1	33.5	41	SI/LD
	clone96	5720	0	98	2	0	-51	-1	hetero SI		102	47.4	115	hetero SI
Biological replicate 2	clone2	4810	0	99	0	0	-12	-66	hetero SI		58.7	31.8	85	hetero SI
	clone3	2901	0	98	2	0	-12		homo SI	SHIFT	73.7	52.5	40	SI/LD
	clone4	NA							no band		244	251	-3	LD/LD
	clone5	12375	0	100	0	0	-1		homo SI		649	462	40	SI/LD
	clone7	10503	0	100	0	0	-8		homo SI		486	374	30	SI/LD
	clone8	14646	0	100	0	0	-52	-52	homo SI		481	355	35	SI/LD
	clone9	1485	0	93	7	0	-1		homo SI		58.6	31.5	86	homo SI
	clone10	1274	0	98	2	0	9		homo SI		66	49.7	33	SI/LD
	clone11	3968	1	54	3	42	-1	HBD	SI/HBD		73.4	41.6	76	SI/HBD
	clone12	11823	0	99	0	1	-26	-1	hetero SI		848	498	70	hetero SI
	clone13	8722	0	99	0	0	-12	2	hetero SI		269	152	77	hetero SI
	clone14	14029	0	99	0	0	-5	-21	hetero SI		549	360	53	hetero SI
	clone15	7886	0	6	1	92	HBD		HBD/HBD	SHIFT	236	151	56	HBD/LD
	clone17	4348	0	99	1	0	-8	-12	hetero SI		177	91.8	93	hetero SI
	clone18	6735	0	99	0	0	-3		homo SI		444	332	34	SI/LD
	clone19	3951	0	99	0	0	-16		homo SI		686	479	43	SI/LD
	clone20	4062	0	58	1	41	HBD	-18	SI/HBD		139	75.5	84	SI/HBD
	clone21	10810	0	3	1	96	HBD		HBD/HBD	NO BAND	594	436	36	HBD/LD
	clone22	8750	0	47	1	52	-1	HBD	SI/HBD		157	80.7	95	SI/HBD
	clone23	4749	0	100	0	0	-1	-12	hetero SI		809	444	82	hetero SI
	clone24	4563	0	99	0	0	-9	-9	homo SI		500	272	84	homo SI
	clone27	6099	0	99	0	0	-1	-5	hetero SI		393	201	96	hetero SI
	clone29	3395	1	98	1	0	-12	1	hetero SI		15	6.9	117	hetero SI
	clone30	6382	0	100	0	0	-15	-3	hetero SI		569	304	87	hetero SI
	clone31	6289	0	98	0	2	-2	1	hetero SI		418	225	86	hetero SI
	clone33	1655	0	99	1	0	-7		homo SI	SHIFT	362	252	44	SI/LD
	clone34	5984	0	99	0	0	-21		homo SI		231	124	86	homo SI
	clone35	4625	0	99	1	0	-2		homo SI	SHIFT	425	296	44	SI/LD
	clone36	9555	0	100	0	0	-16	-1	hetero SI		281	142	98	hetero SI
	clone37	8520	0	100	0	0	-42	-23	hetero SI		683	379	80	hetero SI
	clone38	2407	0	100	0	0	-10	-1	hetero SI		629	356	77	hetero SI
	clone39	6116	0	62	1	37	-39	HBD	SI/HBD		430	219	96	SI/HBD
	clone40	6341	0	99	0	0	-5		homo SI		103.4	69.3	49	SI/LD
	clone41	5234	0	99	1	0	-2	-12	hetero SI		371	204	82	hetero SI
	clone42	4607	0	99	1	0	-8		homo SI		134.9	91.7	47	SI/LD
	clone43	4767	0	100	0	0	-12		homo SI	SHIFT	461	316	46	SI/LD
	clone44	6878	0	100	0	0	-15		homo SI		135.7	93.9	45	SI/LD
	clone45	7225	0	100	0	0	-12	-8	hetero SI		244	124	97	hetero SI
	clone46	10316	0	100	0	0	-8	-1	hetero SI		331	173	91	hetero SI
	clone47	NA							no band		331	334	-1	LD/LD
	clone49	4609	0	99	0	0	-8	-1	hetero SI		270	139	94	hetero SI
	clone50	5594	0	100	0	0	-15		homo SI	SHIFT	411	280	47	SI/LD
	clone51	4590	0	100	0	0	-1	3	hetero SI		291	150	94	hetero SI
	clone53	5278	0	100	0	0	-12		homo SI		398	216	84	homo SI
	clone54	6535	0	100	0	0	-10	-1	hetero SI		379	211	80	hetero SI
	clone55	5860	0	100	0	0	-15	-21	hetero SI		460	241	91	hetero SI
	clone56	7781	0	100	0	0	-2	1	hetero SI		1007	538	87	hetero SI
	clone57	5975	0	100	0	0	-4	-8	hetero SI		604	323	87	hetero SI
	clone58	5954	0	100	0	0	-10	-12	hetero SI		444	225	97	hetero SI
	clone59	5437	0	100	0	0	-12	-3	hetero SI		483	231	109	hetero SI
	clone60	NA							no band		230	228	1	LD/LD
	clone61	6106	0	99	0	1	-26	-1	hetero SI		420	216	94	hetero SI
	clone63	5297	0	100	0	0	-12	-1	hetero SI		436	240	82	hetero SI
	clone64	NA							no band		367	365	1	LD/LD
	clone65	3834	0	99	1	0	-12		homo SI		227	153	48	SI/LD
	clone66	3240	0	98	0	1	-3		homo SI	SHIFT	247	158	56	SI/LD
	clone68	4400	0	100	0	0	-19	-24	hetero SI		132.9	64.2	107	hetero SI
	clone69	4559	0	99	0	0	-1	4	hetero SI		282	149	89	hetero SI
	clone70	4283	0	100	0	0	-55	-12	hetero SI		186	95.2	95	hetero SI
	clone72	4364	0	99	0	1	-1		homo SI		371	251	48	SI/LD
	clone73	5256	0	99	1	0	-1		homo SI		328	228	44	SI/LD
	clone74	4475	0	99	0	0	-8		homo SI	SHIFT	580	400	45	SI/LD
	clone75	4018	0	100	0	0	-3		homo SI		358	247	45	SI/LD
	clone76	4451	0	100	0	0	-12	-19	hetero SI		211	112	88	hetero SI

Supplementary Table S4. Identification of colony genotype in SCD HSPCs with R-66S RNP and ssODN delivery

			S-R NGS								ddPCR			
Sample	clone#	read#	%SCD(GtG)	%SI	%HDR(Gaa)	%HBD(GaG)	allele1	allele2	S-R NGS genotype	L-R PCR	HBB	RRP30	%HBB	Correct genotype
Biological replicate1	clone1	4678	0	1	3	96	HBD		HBD/HBD		1974	1613	22	HBD/LD
	clone2	43318	0	0	100	0	HDR		HDR/HDR		1117	634	76	HDR/HDR
	clone9	23978	0	1	99	0	HDR		HDR/HDR		1153	611	89	HDR/HDR
	clone17	17484	0	100	0	0	-1		homo SI	SHIFT	1412	932	52	SI/LD
	clone18	18629	0	99	1	0	-12		homo SI		845	459	84	homo SI
	clone19	16592	0	52	48	0	HDR	-1	HDR/SI		1212	619	96	HDR/SI
	clone20	10831	0	100	0	0	-7	-12	hetero SI		1226	607	102	hetero SI
	clone21	13113	0	57	42	0	HDR	-12	HDR/SI		962	468	106	HDR/SI
	clone22	13899	0	0	99	0	HDR		HDR/HDR	SHIFT	962	650	48	HDR/LD
	clone23	15510	0	0	99	0	HDR		HDR/HDR		1079	758	42	HDR/LD
	clone24	13779	0	95	5	0	-8	-15	hetero SI		2175	1263	72	hetero SI
	clone25	15957	0	0	99	0	HDR		HDR/HDR		1823	982	86	HDR/HDR
	clone26	9414	0	3	97	0	HDR		HDR/HDR		853	432	97	HDR/HDR
	clone27	15743	0	52	48	0	HDR	-1	HDR/SI		1479	771	92	HDR/SI
	clone28	21795	0	0	100	0	HDR		HDR/HDR		3610	1872	93	HDR/HDR
	clone30	16217	0	0	100	0	HDR		HDR/HDR	SHIFT	1154	783	47	HDR/LD
	clone31	14441	0	99	1	0	-52	42	hetero SI		1568	834	88	hetero SI
	clone32	17513	0	0	100	0	HDR		HDR/HDR		2134	1177	81	HDR/HDR
	clone33	20830	0	0	100	0	HDR		HDR/HDR		1428	738	93	HDR/HDR
	clone34	20971	0	1	99	0	HDR		HDR/HDR		2431	1283	89	HDR/HDR
	clone35	17292	0	1	98	0	HDR		HDR/HDR		2342	1273	84	HDR/HDR
	clone37	13459	0	1	98	0	HDR		HDR/HDR		1049	530	98	HDR/HDR
	clone38	14719	0	99	1	0	-1	-19	hetero SI		661	329	101	hetero SI
	clone39	12416	0	0	99	0	HDR		HDR/HDR		581	382	52	HDR/LD
	clone40	13418	0	54	46	0	HDR	-9	HDR/SI		2639	1412	87	HDR/SI
	clone41	4389	0	2	98	0	HDR		HDR/HDR		1320	654	102	HDR/HDR
	clone42	16738	2	0	97	0	HDR		HDR/HDR		1746	915	91	HDR/HDR
	clone44	12710	0	0	100	0	HDR		HDR/HDR	SHIFT	774	522	48	HDR/LD
	clone45	7900	0	2	98	0	HDR		HDR/HDR		196	126	56	HDR/LD
	clone46	10436	0	5	95	0	HDR		HDR/HDR		839	432	94	HDR/HDR
	clone47	11672	0	93	6	0	-28		homo SI		1330	916	45	SI/LD
	clone48	8387	0	68	32	0	HDR	-61	HDR/SI		795	411	93	HDR/SI
	clone49	13987	0	1	99	0	HDR		HDR/HDR		1076	546	97	HDR/HDR
	clone50	10612	0	1	99	0	HDR		HDR/HDR	SHIFT	535	378	42	HDR/LD
	clone51	7583	0	1	99	0	HDR		HDR/HDR		517	246	110	HDR/HDR
	clone53	12049	0	1	99	0	HDR		HDR/HDR		451	231	95	HDR/HDR
	clone54	11111	0	53	47	0	HDR	-2	HDR/SI		2426	1265	92	HDR/SI
	clone55	11541	0	1	99	0	HDR		HDR/HDR		1713	936	83	HDR/HDR
	clone56	3099	0	98	2	0	-2		homo SI		1256	639	97	homo SI
	clone57	10484	0	52	48	0	HDR	1	HDR/SI		794	407	95	HDR/SI
	clone58	5366	0	98	2	0	2		homo SI		204	96	113	homo SI
	clone59	7093	0	1	99	0	HDR		HDR/HDR	SHIFT	775	514	51	HDR/LD
	clone60	13089	0	1	99	0	HDR		HDR/HDR		983	470	109	HDR/HDR
	clone61	10092	0	98	1	0	-3	-5	hetero SI		294	144	104	hetero SI
	clone62	12408	0	1	98	0	HDR		HDR/HDR		2319	1131	105	HDR/HDR
	clone63	7031	0	48	52	0	HDR	-1	HDR/SI		239	114	110	HDR/SI
	clone65	8278	0	99	1	0	-8	-62	hetero SI		1654	854	94	hetero SI
	clone66	8905	0	1	98	0	HDR		HDR/HDR		1540	777	98	HDR/HDR
	clone68	12715	0	99	1	0	-2		homo SI		1025	517	98	homo SI
	clone69	12538	0	100	0	0	-4		homo SI	SHIFT	1172	801	46	SI/LD
	clone70	9709	0	99	1	0	-4		homo SI	SHIFT	923	618	49	SI/LD
	clone71	4519	0	55	45	0	HDR	-21	HDR/SI					HDR/SI
	clone72	6002	0	51	49	0	HDR	-19	HDR/SI		738	370	99	HDR/SI
	clone73	8647	0	99	1	0	-1		homo SI		1086	560	94	homo SI
	clone74	6705	0	99	1	0	-1	-21	hetero SI		1524	772	97	hetero SI
	clone75	8822	0	1	99	0	HDR		HDR/HDR		627	318	97	HDR/HDR
	clone76	12438	0	100	0	0	-18	-1	hetero SI		1152	579	99	hetero SI
	clone77	9828	0	49	51	0	HDR	-1	HDR/SI		1497	711	111	HDR/SI
	clone78	7747	0	50	49	0	HDR	-2	HDR/SI		419	205	104	HDR/SI
	clone79	10265	0	97	3	0	-21	-1	hetero SI		1263	624	102	hetero SI
	clone80	8690	0	99	1	0	-1		homo SI	SHIFT	1060	705	50	SI/LD
	clone81	4173	0	98	2	0	-2		homo SI		630	342	84	homo SI
	clone82	25713	0	0	100	0	HDR		HDR/HDR		654	335	95	HDR/HDR
	clone83	10676	39	59	2	0	WT	-24	WT/SI		993	509	95	WT/SI
	clone84	7652	0	48	52	0	HDR	-1	HDR/SI		261	132	98	HDR/SI
	clone85	12217	0	52	48	0	HDR	-1	HDR/SI		765	381	101	HDR/SI
	clone86	7839	0	1	99	0	HDR		HDR/HDR		236	110	115	HDR/HDR
	clone87	13074	0	0	99	0	HDR		HDR/HDR		566	293	93	HDR/HDR
	clone88	11531	0	100	0	0	-1	-20	hetero SI		467	240	95	hetero SI
	clone89	6844	0	99	1	0	2		homo SI	SHIFT	647	422	53	SI/LD
	clone90	10077	0	51	49	0	HDR	-1	HDR/SI		205	111	85	HDR/SI
	clone91	6232	0	52	48	0	HDR	1	HDR/SI		606	312	94	HDR/SI
	clone92	8967	0	4	96	0	HDR		HDR/HDR		407	210	94	HDR/HDR
	clone93	8896	0	50	50	0	HDR	-2	HDR/SI		174	84.8	105	HDR/SI
	clone95	6726	0	57	43	0	HDR	-21	HDR/SI		235	122	93	HDR/SI
	clone96	1902	0	95	5	0	-25		homo SI		299	189	58	SI/LD
	clone97	12906	0	2	98	0	HDR		HDR/HDR		166	87	90	HDR/HDR
	clone98	11782	0	93	7	0	-2		homo SI		615	332	85	homo SI
	clone99	11906	0	3	97	0	HDR		HDR/HDR		314	161	95	HDR/HDR
	clone100	11592	0	45	55	0	HDR	-1	HDR/SI		417	200	109	HDR/SI
	clone101	7816	0	98	2	0	-2		homo SI		1783	958	86	homo SI
	clone102	8987	0	99	1	0	-10	-3	hetero SI		163	87.2	87	hetero SI
	clone103	5321	0	98	2	0	-9	-2	hetero SI		195	105	86	hetero SI
	clone104	7530	0	3	97	0	HDR		HDR/HDR		586	308	90	HDR/HDR
Biological replicate2	clone1	11072	0	54	46	0	HDR	-26	HDR/SI		477	262	82	HDR/SI
	clone2	9896	0	2	98	0	HDR		HDR/HDR	SHIFT	161	96	68	HDR/LD
	clone4	12325	0	1	99	0	HDR		HDR/HDR		585	396	48	HDR/LD
	clone5	17107	0	45	55	0	HDR	-5	HDR/SI		451	241	87	HDR/SI
	clone6	21903	0	45	55	0	HDR	-1	HDR/SI		784	419	87	HDR/SI
	clone7	11926	0	47	52	1	HDR	-1	HDR/SI		406	235	73	HDR/SI
	clone8	17301	0	2	98	0	HDR		HDR/HDR		194	107	81	HDR/HDR
	clone9	5846	0	80	4	15	HDR	-19	HDR/SI		324	182	78	HDR/SI
	clone10	5942	0	10	6	84	HBD		HBD/HBD	SHIFT	348	268	30	HBD/LD
	clone11	20263	0	97	2	0	-21	-21	homo SI		1576	836	89	homo SI
	clone12	18744	0	2	98	0	HDR		HDR/HDR		970	510	90	HDR/HDR
	clone13	13626	0	0	99	0	HDR		HDR/HDR		218	116	88	HDR/HDR
	clone14	22482	0	20	76	3	HDR	-19	HDR/SI		652	349	87	HDR/SI
	clone16	17298	0	53	47	0	HDR	-7	HDR/SI		844	457	85	HDR/SI
	clone17	14390	0	99	1	0	-12	-2	hetero SI		1310	691	90	hetero SI
	clone18	10952	0	92	1	6	-6		homo SI		752	412	83	homo SI
	clone19	17471	0	99	1	0	-1	-2	hetero SI		1462	793	84	hetero SI
	clone20	16319	0	55	45	0	HDR	-26	HDR/SI		2084	1142	82	HDR/SI
	clone21	12192	0	1	93	6	HDR		HDR/HDR		3190	1627	96	HDR/HDR
	clone22	9107	0	2	97	0	HDR		HDR/HDR		2016	1102	83	HDR/HDR
	clone23	18434	0	1	99	0	HDR		HDR/HDR		1333	728	83	HDR/HDR
	clone24	18429	0	99	1	0	1		homo SI	SHIFT	835	550	52	SI/LD
	clone25	14567	0	98	2	0	1		homo SI	SHIFT	613	400	53	SI/LD
	clone26	14684	0	1	99	0	HDR		HDR/HDR		509	266	91	HDR/HDR
	clone27	12926	0	50	50	0	HDR	-2	HDR/SI		782	408	92	HDR/SI
	clone29	14915	0	99	1	0	-12		homo SI	SHIFT	691	463	49	SI/LD
	clone30	23070	97	1	1	0	WT		WT/WT		1004	521	93	WT/WT
	clone31	15924	0	50	50	0	HDR	-1	HDR/SI		686	370	85	HDR/SI
	clone32	12332	0	99	1	0	-3		homo SI		394	270	46	SI/LD
	clone33	10604	0	98	2	0	-18	-1	hetero SI		68.3	31.6	116	hetero SI
	clone34	14311	97	2	1	0	WT		WT/WT		358	190	88	WT/WT
	clone35	13327	0	42	57	1	HDR	18	HDR/SI		797	422	89	HDR/SI
	clone36	7946	0	2	98	0	HDR		HDR/HDR		680	368	85	HDR/HDR
	clone37	18761	0	0	99	0	HDR		HDR/HDR		881	468	88	HDR/HDR
	clone38	16002	1	50	49	0	HDR	-1	HDR/SI		1220	642	90	HDR/SI
	clone39	14267	0	99	1	0	-19	-1	hetero SI		902	456	98	hetero SI
	clone40	16338	0	50	50	0	HDR	-1	HDR/SI		1745	885	97	HDR/SI
	clone41	12396	0	99	1	0	-1	-2	hetero SI		195	99	97	hetero SI
	clone42	17344	0	1	99	0	HDR		HDR/HDR		664	347	91	HDR/HDR
	clone43	14808	0	50	49	0	HDR	-2	HDR/SI		2151	1088	98	HDR/SI
	clone44	16073	0	1	99	0	HDR		HDR/HDR		484	240	102	HDR/HDR
	clone45	20565	0	99	1	0	-21		homo SI		1296	618	110	homo SI
	clone46	13772	0	98	2	0	-1		homo SI		1095	608	80	homo SI
	clone47	12353	0	52	46	1	HDR	-3	HDR/SI		799	426	88	HDR/SI
	clone48	10637	0	98	2	0	-1	-12	hetero SI		301	169	78	hetero SI
	clone50	11455	0	17	83	0	HDR	33	HDR/SI		482	249	94	HDR/SI
	clone51	18042	0	1	99	0	HDR		HDR/HDR	NO BAND	713	357	100	HDR/HDR
	clone52	11818	0	51	49	0	HDR	-3	HDR/SI		433	233	86	HDR/SI
	clone53	16574	0	0	100	0	HDR		HDR/HDR		257	175	47	HDR/LD
	clone54	14224	91	8	1	0	WT		WT/WT		951	486	96	WT/WT
	clone55	14206	0	55	45	0	HDR	-8	HDR/SI		1293	666	94	HDR/SI
	clone56	12857	0	96	4	0	-2		homo SI		1168	620	88	homo SI
	clone58	14047	0	52	48	0	HDR	-1	HDR/SI		251	131	92	HDR/SI
	clone59	14104	0	1	98	0	HDR		HDR/HDR		784	373	110	HDR/HDR
	clone60	19411	0	99	1	0	-1		homo SI		544	350	55	SI/LD
	clone62	11498	0	51	49	0	HDR	1	HDR/SI		438	218	101	HDR/SI
	clone63	16758	0	100	0	0	-25		homo SI		1321	900	47	SI/LD
	clone64	13129	0	50	50	0	HDR	-1	HDR/SI		1898	957	98	HDR/SI
	clone65	9502	0	5	94	0	HDR		HDR/HDR		152.1	81.1	88	HDR/HDR
	clone66	11975	0	1	99	0	HDR		HDR/HDR		699	364	92	HDR/HDR
	clone67	11945	94	5	1	0	WT		WT/WT		425	222	91	WT/WT
	clone68	12080	0	96	3	0	-19	-1	hetero SI		844	425	99	hetero SI
	clone69	14608	0	55	45	0	HDR	-1	HDR/SI		199	97	105	HDR/SI
	clone70	29444	0	52	47	0	HDR	-1	HDR/SI		2228	1158	92	HDR/SI
	clone71	18735	0	52	48	0	HDR	-2	HDR/SI		274	144	90	HDR/SI
	clone73	15227	0	50	50	0	HDR	-4	HDR/SI		310	162	91	HDR/SI
	clone74	9173	0	98	2	0	-2		homo SI	SHIFT	128.6	86	50	SI/LD
	clone75	20346	1	98	0	1	-19	-1	hetero SI		1827	962	90	hetero SI
	clone76	15580	0	1	98	0	HDR		HDR/HDR		929	557	67	HDR/LD
	clone77	14963	0	99	1	0	-36	-10	hetero SI		904	448	102	hetero SI
	clone78	18455	0	52	48	0	HDR	-1	HDR/SI		656	339	94	HDR/SI
	clone79	19878	0	1	99	0	HDR		HDR/HDR		322	172	87	HDR/HDR
	clone80	14052	0	99	1	0	-11	10	hetero SI		528	276	91	hetero SI

Table S5. List of primer sequences

	Target	Assay	Amplicon size (bp)	Sequences
HBB_NGS_F				TCT ACA GTC CGA CGA TCA CCC AAG AGT CTT CTC TGT CTC C
HBB_NGS_R	HBB	NGS	300	GAC GTG TGC TCT TCC GAT CGT TGG CCA ATC TAC TCC CAG G
OT18_NGS_F				TCT ACA GTC CGA CGA TCAGTCCCTCAACATTTGCTTATCTGAGAAGGATT
OT18_NGS_R	R66S OT18	NGS	300	GAC GTG TGC TCT TCC GAT CCCTTGCTAGAGAGGACAACAGTCA
HBG_NGS_F				TCT ACA GTC CGA CGA TCA TCG GAA CAA GGC AAA GGC T
HBG_NGS_R	HBG	NGS	300	GAC GTG TGC TCT TCC GAT CAG ACG TTC CAG AAG CGA GTG
BCL11A_NGS_F				TCT ACA GTC CGA CGA TCG CTG ATT CCA GTG CAA AGT CC
BCL11A_NGS_R	BCL11A	NGS	300	GAC GTG TGC TCT TCC GAT CCA CCA AGA GAG CCT TCC GAA
HBB_long_F				AGTGGGGCTGGAATAAAAGTAGAAT
HBB_long_R	HBB	LongAmp-Seq	5490	TTTTTCCTTTTGTTGCCTTTGCTTC
OT18_long_F				ATG TGG AGT CTT GGC AGA GC
OT18_long_R	R66S OT18	LongAmp-Seq	5046	CCA CTA AGC CAT GTG CAG GA
HBG1_long_F				ACG GCA TCT GGC TTT TCT CA
HBG1_long_R	HBG1	LongAmp-Seq	6718	GCG TGA AAA GCA ATT GCA GC
HBG_10kb_F				GGT TCC CCA GTG AGG ATG TG
HBG_10kb_R	HBG1&2	LongAmp-Seq	10,339	CCC CAG GTC TTC ACT GAA CC
BCL11A_long_F				TCA ATT CAG GAG CGG CAG TT
BCL11A_long_R	BCL11A	LongAmp-Seq	4268	GTT GCC ATG TGG GTT GTG AC
GFPBFP inout_F3				GCCAGGCATCTTGAGGTTCT
GFPBFP inout_R3	GFPBFP	LongAmp-Seq	9423	CCACCTCCCCCAGAGTCTTA
HBB_211bp_F				GCAGAGCCATCTATTGCTTACA
HBB_211bp_R	HBB	ddPCR	211	CTCCACATGCCCAGTTTCTATT
HBG_ddPCR_F2				GCC CCT TCC CCA CAC TAT CT
HBG_ddPCR_R2	HBG	ddPCR	225	AGA CGT TCC AGA AGC GAG TG
RPP30_F				GAT TTG GAC CTG CGA GCG
RPP30_R	RPP30	ddPCR	62	GCG GCT GTC TCC ACA AGT
CACNA1C_ref_F				CTCTCCTATAGCTGCATGCACACCC
CACNA1C_ref_R	CACNA1C	ddPCR	293	GAGAGTGCCTGGTCTTTACTGCAGG

Table S6: Details of large insertions in SCD HSPCs treated by R-66S RNP

UMI ID	Chr	Start position	End position	Match	Mismatch	Strand	nearest gene	Other mutation on the allele	Feature
umi4269	chr11	4494366	5248397	330	19	-	HBB	Intermediate deletion	
umi2381	chr11	4761870	5248396	315	10	-	HBB	Intermediate deletion	
umi6451	chr11	5247913	5248396	431	18	-	HBB	Intermediate deletion	
umi334	chr11	5245393	5245874	479	2	+	HBB	LD	
umi53	chr11	5247543	5248223	677	3	+	HBB	LD	
umi26804	chr11	5247543	5248223	676	4	+	HBB	LD	
umi1162	chr11	5247598	5248225	625	1	+	HBB	LD	
umi393	chr11	5247598	5248225	625	1	+	HBB	LD	
umi136	chr11	5247618	5248228	606	4	+	HBB	LD	
umi3148	chr11	5247755	5248203	447	1	+	HBB	LD	
umi1509	chr11	5247918	5248220	302	0	+	HBB	LD	
umi604	chr11	5247918	5248220	302	0	+	HBB	LD	
umi573	chr11	5247918	5248220	302	0	+	HBB	LD	
umi511	chr11	5247918	5248219	301	0	+	HBB	LD	
umi23398	chr11	5248024	5248219	195	0	+	HBB	LD	
umi464	chr11	5248231	5248604	367	1	+	HBB	LD	
umi407	chr11	5248231	5248604	368	0	+	HBB	LD	
umi1575	chr11	5248232	5249240	1000	4	+	HBB	LD	
umi124	chr11	5248232	5249240	1000	7	+	HBB	LD	
umi21495	chr11	5248232	5249240	999	5	+	HBB	LD	
umi14733	chr11	5248232	5248716	482	2	+	HBB	LD	
umi469	chr11	5248232	5248716	482	2	+	HBB	LD	
umi468	chr11	5248232	5248716	482	2	+	HBB	LD	
umi24156	chr11	5248232	5248716	482	2	+	HBB	LD	
umi27345	chr11	5248232	5248530	297	1	+	HBB	LD	
umi21585	chr11	5248233	5248990	749	7	+	HBB	LD	
umi1131	chr11	5248235	5248902	449	2	+	HBB	LD	
umi5	chr11	5248235	5248902	449	2	+	HBB	LD	
umi21336	chr11	5248237	5248582	344	1	+	HBB	LD	
umi4647	chr11	5248482	5248556	74	0	-	HBB	LD	
umi21538	chr11	5248592	5249070	470	5	+	HBB	LD	
umi231	chr11	5248592	5249070	470	5	+	HBB	LD	
umi240	chr11	5248595	5248745	149	1	+	HBB	LD	
umi512	chr11	5248984	5250810	495	2	-	HBB	LD	
umi2908	chr11	5247926	5248230	304	0	-	HBB	Small INDEL or unmodified	
umi1361	chr11	5247926	5248230	304	0	-	HBB	Small INDEL or unmodified	
umi670	chr11	5248036	5248381	195	0	-	HBB	Small INDEL or unmodified	
umi3677	chr11	5248038	5248226	188	0	-	HBB	Small INDEL or unmodified	
umi4454	chr11	5248089	5248212	123	0	-	HBB	Small INDEL or unmodified	
umi24215	chr11	5248240	5248397	156	1	-	HBB	Small INDEL or unmodified	
umi29106	chr11	5248241	5248398	156	1	-	HBB	Small INDEL or unmodified	
umi350	chr11	5248243	5248408	165	0	-	HBB	Small INDEL or unmodified	
umi5376	chr11	5248258	5248396	138	0	-	HBB	Small INDEL or unmodified	
umi1930	chr11	5248464	5248961	491	5	-	HBB	Small INDEL or unmodified	
umi324	chr11	5248541	5248595	54	0	+	HBB	Small INDEL or unmodified	
umi24482	chr11	5255654	5255724	70	0	-	HBB	Small INDEL or unmodified	
umi24256	chr1	156274244	156274482	238	0	-	CCT3	Intermediate deletion	
umi15339	chr1	156274244	156274482	238	0	-	CCT3	Intermediate deletion	
umi8842	chr1	249167824	249167901	77	0	+	ZNF692	LD	telomeric end
umi4008	chr1	249167824	249167901	77	0	+	ZNF692	LD	telomeric end
umi114	chr1	249167824	249167901	77	0	+	ZNF692	LD	telomeric end
umi96	chr1	249167824	249167901	77	0	+	ZNF692	LD	telomeric end
umi4543	chr2	209079878	209080191	311	2	+	IDH1	Intermediate deletion	
umi28409	chr2	43294306	43294511	205	0	+	ZFP36L2	Small INDEL or unmodified	
umi18570	chr3	195360569	195360868	299	0	-	APOD	Intermediate deletion	
umi29378	chr3	186594586	186594761	175	0	+	ADIPOQ	Small INDEL or unmodified	
umi2367	chr3	186594586	186594761	175	0	+	ADIPOQ	Small INDEL or unmodified	
umi5112	chr4	79549296	79549509	213	0	+	ANXA3	Small INDEL or unmodified	
umi384	chr6	145112099	145112419	319	1	-	UTRN	Intermediate deletion	
umi5260	chr6	44013226	44013364	138	0	+	MRPL14	Small INDEL or unmodified	
umi1625	chr11	50534706	50534911	205	0	-	OR4C45	LD	repetitive region
umi423	chr11	50534706	50534911	205	0	-	OR4C45	LD	repetitive region
umi4649	chr15	83768216	83768439	223	0	+	TM6SF1	Small INDEL or unmodified	
umi23013	chr19	17911596	18068193	289	0	-	B3GNT3	Intermediate deletion	
umi4405	chr19	649811	649953	142	0	-	RNF126	Small INDEL or unmodified	repetitive region
umi2383	chr19	649811	649953	142	0	-	RNF126	Small INDEL or unmodified	repetitive region
umi19487	chr19	649811	649953	142	0	-	RNF126	Small INDEL or unmodified	repetitive region

Table S7: LongAmp-seq sequencing depth and read numbers

Sample	Amplicon size	#raw reads	#merged reads	%merged	#reads spanning cutsite	%reads spanning cutsite	#unsplit reads	#split reads
R-66S_RNP1	5189	662665	343775	51.88	26532	7.72	19570	6962
R-66S_RNP2	5189	560531	263200	46.96	19188	7.29	14225	4963
R-66S_RNP+ssODN1	5189	592468	321981	54.35	23996	7.45	22462	1534
R-66S_RNP+ssODN2	5189	282153	129842	46.02	9703	7.47	9143	560
R-02_RNP1	5189	847726	440590	51.97	32414	7.36	26306	6108
R-02_RNP2	5189	851877	457074	53.65	34116	7.46	28182	5934
SD-02_RNP1	6578	446223	202732	45.43	11741	5.79	9473	2268
SD-02_RNP2	6578	585425	255978	43.73	13462	5.26	11062	2400
BCL11A_RNP1	4351	543214	280898	51.71	24211	8.62	20334	3877
BCL11A_RNP2	4351	746080	382749	51.30	33449	8.74	26517	6932
